# An embedding-based framework enables statistical testing of gene-set function hypotheses inferred by large language models

**DOI:** 10.1038/s41467-026-75972-z

**Published:** 2026-07-30

**Authors:** Yanhao Tan, Li-Ju Wang, Tianyuzhou Liang, Ying-Ju Lai, Chien-Hung Shih, Yibing Guo, Tyler M. Yasaka, George C. Tseng, Yu-Chiao Chiu

**Affiliations:** 1 UPMC Hillman Cancer Center, University of Pittsburgh School of Medicine, Pittsburgh, PA USA; 2 Department of Medicine, University of Pittsburgh School of Medicine, Pittsburgh, PA USA; 3 Integrative Systems Biology Program, University of Pittsburgh School of Medicine, Pittsburgh, PA USA; 4 Department of Biostatistics and Health Data Science, University of Pittsburgh, Pittsburgh, PA USA; 5 Department of Human Genetics, School of Public Health, University of Pittsburgh, Pittsburgh, PA USA; 6 Medical Scientist Training Program, University of Pittsburgh School of Medicine, Pittsburgh, PA USA; 7 Department of Computational and Systems Biology, University of Pittsburgh School of Medicine, Pittsburgh, PA USA

**Keywords:** Functional clustering, Machine learning, Gene ontology

## Abstract

Emerging large language models (LLMs) can infer gene functions directly from gene lists, enabling hypothesis generation without predefined gene sets. However, these LLM-derived predictions are qualitative, and principled statistical validation is lacking. Here, we develop an embedding-based statistical framework that transforms gene and function descriptions into vector representations, enabling statistical testing of gene-gene and gene-function relationships and quantitative prioritization of de novo functional hypotheses inferred by LLMs. We benchmark seven state-of-the-art embedding models using curated and retrieval-augmented literature-derived gene descriptions across diverse biological contexts. OpenAI’s text-embedding-3-large and Google’s gemini-embedding-001 perform best, capturing gene-gene functional relationships in 88.7-92.5% of Gene Ontology biological processes and approximately 98.6% of canonical pathways. In gene-function association analyses, these models achieve high sensitivity (95.2-98.4%) and specificity (72.7-84.3%). Through contamination analysis and evaluation using experimentally informed protein assembly gene sets, our framework distinguishes biologically meaningful LLM-inferred hypotheses from noise, outperforming confidence-based inference and conventional enrichment analysis. We further develop the open-source R package DEGEmbedR and demonstrate its utility for interpreting a drug perturbation-derived differentially expressed gene (DEG) signature lacking significant conventional enrichment results. Together, these results establish LLM-derived embeddings as a quantitative foundation for functional genomics and the statistical validation of LLM-based gene function inference.

## Abstract (web summary)

Whilst large language models can infer gene functions from gene lists, these predictions lack validation. Here the authors develop a framework to transform gene and function descriptions into vector representations, enabling statistical testing of gene-gene and gene-function relationships.

## Introduction

Understanding gene function is fundamental to elucidating molecular mechanisms, identifying biomarkers, and developing targeted therapies^1^. In functional genomics, gene function is often interrogated through high-throughput experiments that yield gene lists or ranked signatures. Interpreting these results requires quantitative statistical testing to determine whether an observed gene list supports a specific biological mechanism. Traditionally, this is performed using gene set enrichment methods such as overrepresentation analysis (ORA) and Gene Set Enrichment Analysis (GSEA)^2^ with curated annotations from resources such as the Molecular Signatures Database (MSigDB)^3^. While widely adopted and effective, these approaches rely on manually curated gene sets that are often incomplete, lag behind emerging biology, and cannot flexibly represent context-dependent or newly described functions in the literature^4–7^. These limitations motivate scalable approaches that expand functional interpretation beyond existing annotation catalogs.

Recent advances in large language models (LLMs) have enabled gene-set-free functional inference, allowing shared biological themes to be inferred directly from gene lists and expressed as free-text mechanistic hypotheses^5,6^. This capability can generate de novo functions that are not yet represented in curated databases. In addition, retrieval-augmented generation (RAG) can strengthen LLM inference by grounding predictions in recent biomedical literature, offering a scalable strategy to overcome the static nature of manual curation and incorporate emerging discoveries^8,9^. However, these predictions remain largely qualitative, often accompanied by heuristic confidence scores that depend on prompting strategies and are not calibrated for statistical decision-making. Consequently, even when an LLM proposes a plausible gene-set function, there is typically no principled way to statistically test whether it is supported by the input gene list or to distinguish signal from noise. Filling this missing hypothesis-testing layer is essential to make LLM-generated gene-set functions reliable and actionable in functional genomics.

One promising route toward quantitative testing is to use LLM-derived semantic embeddings, which map highly complex biological text into continuous vector representations that capture contextual and relational information in a deterministic manner. Embeddings have been used to uncover functional relationships such as gene-gene interactions and context-specific gene roles^10,11^, suggesting that they can provide a quantitative substrate for statistical analysis. However, the extent to which embeddings consistently represent functional structure across diverse biological contexts, and whether they support rigorous hypothesis testing for both curated and de novo functions, remains insufficiently characterized. Systematic evaluation is therefore needed to determine whether embedding-based similarity can reliably encode gene-gene and gene-function relationships with adequate sensitivity and specificity for downstream inference.

**Fig. 1:**
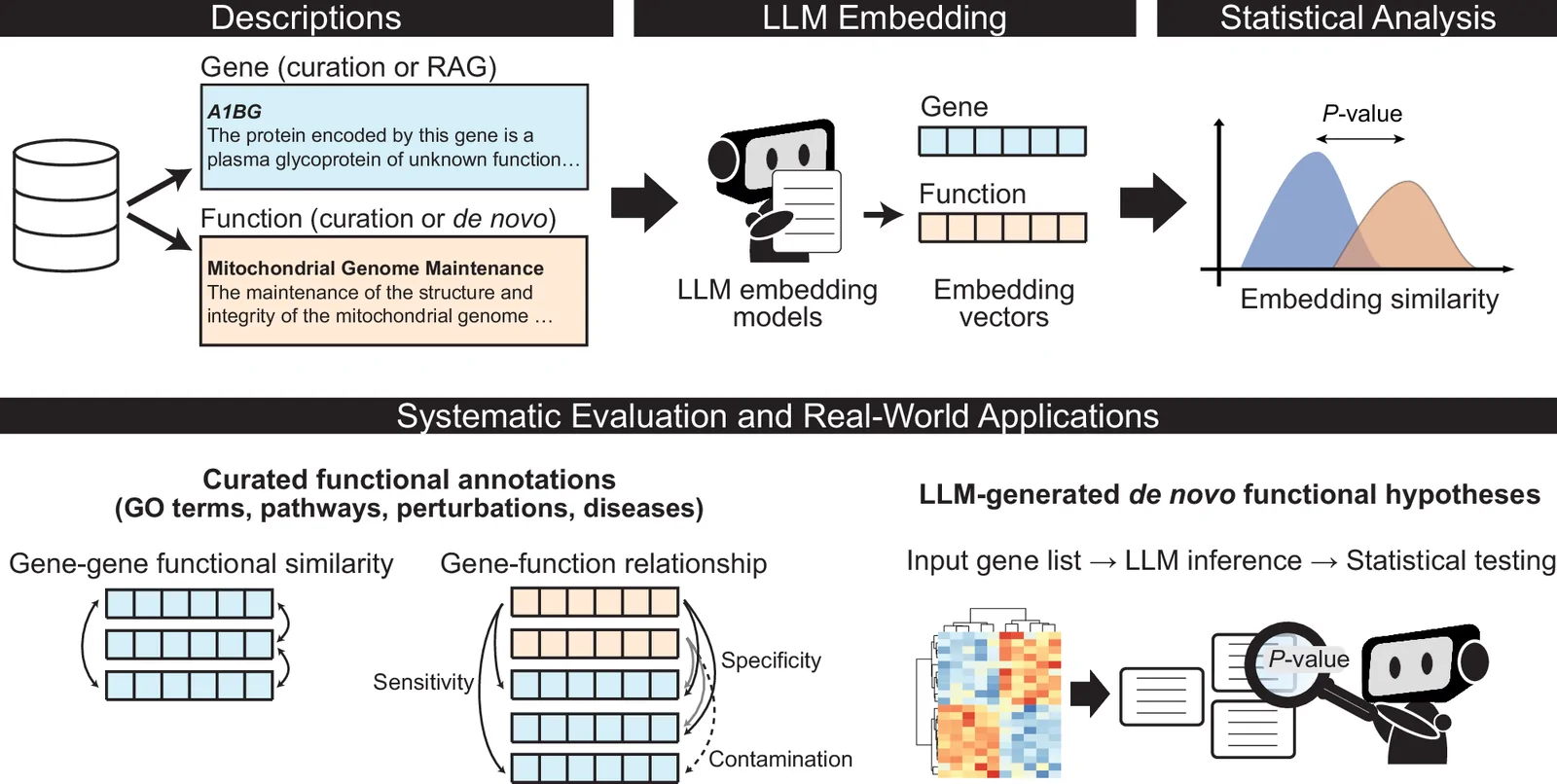
Computational framework for statistical evaluation of LLM-inferred de novo functional hypotheses using LLM-derived embeddings. The framework consists of three core stages: (1) extraction of textual descriptions for genes and biological functions from curated databases, retrieval-augmented generation (RAG), or LLM de novo generation; (2) transformation of these descriptions into embedding vectors using LLM embedding models; and (3) computation of cosine similarities between gene-gene and gene-function pairs, followed by statistical testing. This design enables systematic evaluation of gene-gene functional similarity and gene-function relationships, including sensitivity, specificity, and robustness under contamination. Importantly, the framework supports both curated functional annotations and LLM-generated de novo functional hypotheses, allowing inferred functions from input gene lists to be converted into quantitatively testable statistical hypotheses. A detailed flowchart is provided in Supplementary Fig. 3.

Here, we address the central question of whether LLM-derived embeddings can provide a generalizable quantitative foundation for statistically testing gene-set functions inferred from gene lists. As illustrated in Fig. 1, we develop a statistical framework that enables hypothesis testing of gene-gene and gene-function relationships using embedding similarity distributions. We benchmark seven state-of-the-art embedding models, including proprietary and open-source LLMs (Supplementary Table 1), together with a non-LLM baseline. To reflect diverse biological contexts, we evaluate gene embeddings generated using multiple sources of gene descriptions, including curated summaries from NCBI and literature-derived descriptions produced using RAG. Using curated biological process and canonical pathway annotations as controlled references, we assess whether embeddings capture gene-gene functional relationships at scale and quantify the sensitivity and specificity of gene-function associations across diverse biological contexts. Through systematic contamination analyses, we further examine the robustness of the framework and its ability to distinguish biologically meaningful gene lists from noise, a critical requirement for high-throughput functional genomics applications. We further extend beyond curated annotations by applying the framework to experimentally informed protein assembly gene sets, thereby enabling statistical evaluation of de novo functional hypotheses generated by an LLM-based inference method. The resulting framework enables quantitative prioritization and statistical testing of de novo functional hypotheses in practical functional genomics workflows. We additionally develop the open-source R package DEGEmbedR and demonstrate its application in a drug perturbation-derived differentially expressed gene (DEG) signature lacking significant conventional enrichment results. Together, our results establish embedding-based statistical testing as a general validation layer for emerging LLM-driven gene function inference, enabling quantitative and scalable hypothesis assessment in functional genomics.

## Results

### Evaluation of gene embeddings across curated and retrieval-generated gene descriptions

**Fig. 2:**
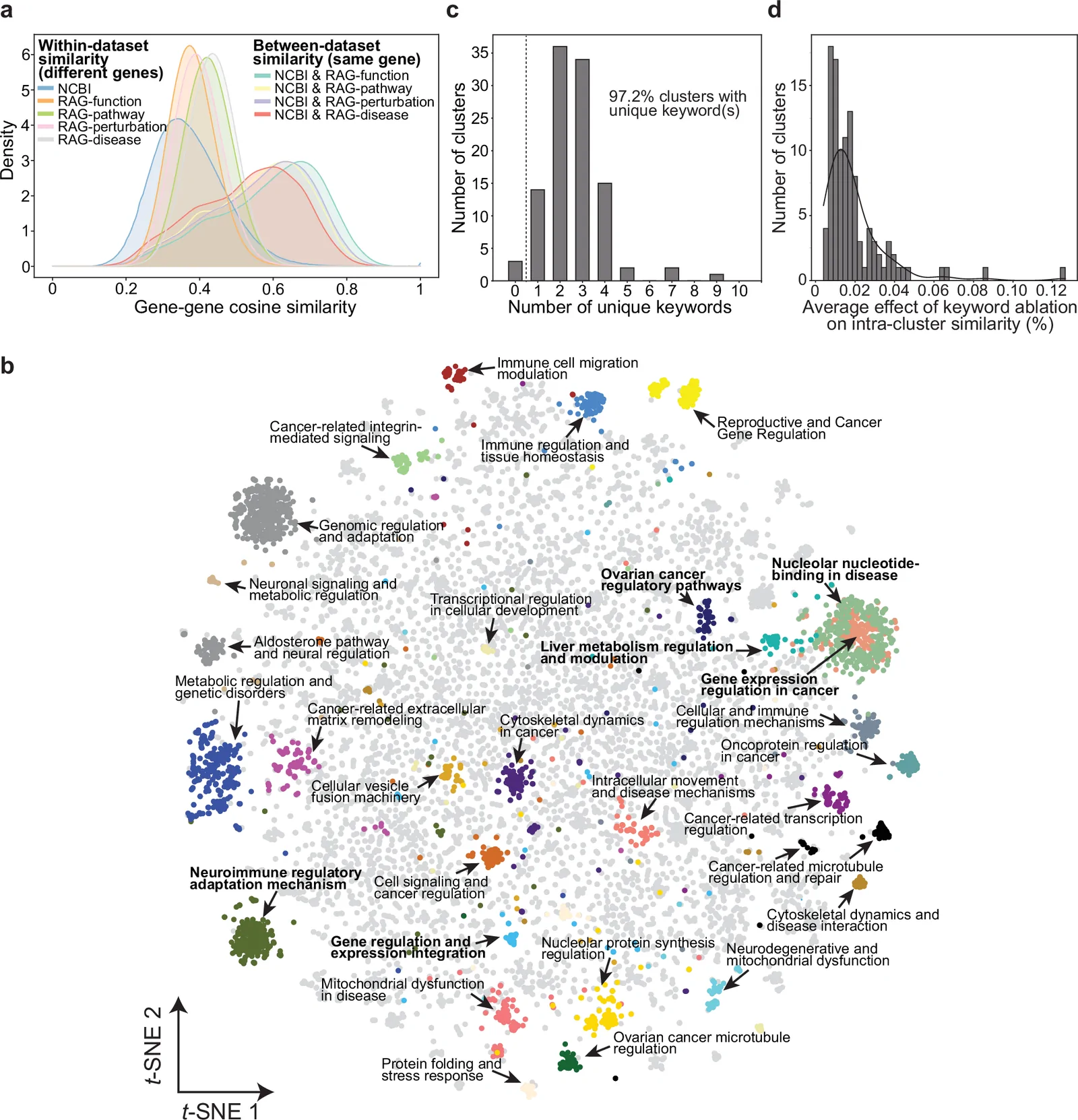
Overview and interpretation of LLM-derived gene embeddings. **a** Density distributions of gene-gene cosine similarity comparing same-gene similarity for overlapping genes between pairs of description sources (NCBI and RAG-derived descriptions including function, pathway, perturbation, and disease contexts) with different-gene similarity within each source. Density plots were generated using Gaussian kernel density estimation implemented in SciPy. **b** *t*-SNE visualization of 18,723 gene embeddings generated by OpenAI-TE3 using RAG-function descriptions and clustered into 107 groups in the original 3072-dimensional embedding space. The two-dimensional *t*-SNE projection is shown for visualization only and does not reflect exact high-dimensional distances. The 30 most compact clusters are annotated using representative biological terms derived from named entity recognition and frequency analysis of gene descriptions. Clusters lacking significant overlap with GO biological process terms are indicated in bold. Complete cluster annotations are provided in Supplementary Data 1. **c** Distribution of the number of cluster-specific keywords among the top 10 representative terms per cluster. **d** Distribution of average changes in mean intra-cluster cosine similarity following keyword ablation. For each cluster, each of its 10 most representative terms was removed from all member gene descriptions, embeddings were regenerated, and the resulting change in mean intra-cluster cosine similarity was calculated. Source data are provided as a Source Data file.

LLM-based embeddings represent the semantic and contextual information of unstructured text in continuous vector spaces. To establish an initial characterization of these embeddings, we evaluated the 3072-dimensional embeddings generated by text-embedding-3-large (OpenAI-TE3), selected for the reported superior performance of OpenAI LLMs and embeddings in prior functional studies^5,6,10^. We generated gene embeddings from summary descriptions of human protein-coding genes obtained from two sources: curated summaries from the NCBI Gene database and de novo descriptions generated using RAG (18,753 and 18,288–18,723 genes, respectively; Supplementary Table 2). For RAG, we synthesized four complementary descriptions per gene using sentences retrieved from PubMed that capture functional roles, pathways, perturbations, and disease associations (RAG-function, RAG-pathway, RAG-perturbation, and RAG-disease; see “Methods”). This was implemented using a framework we previously established and quantitatively evaluated and optimized for therapeutic mechanism inference, including systematic assessment and mitigation of potential hallucinations and other generation errors^9^. To evaluate consistency across sources, we computed cosine similarity, a widely used similarity metric in embedding analysis^12,13^, for each gene across its representations. Although the descriptions emphasize different functional aspects, embeddings of the same gene between NCBI and each RAG strategy were substantially more similar (median, 0.55–0.61; Fig. 2a) than embeddings of different genes within each source (median, 0.36–0.43). Comparisons with additional curated databases, including Alliance of Genome Resources and UniProt, showed comparable trends (Supplementary Fig. 1). Moreover, embeddings of the same gene between different RAG-generated descriptions exhibited moderately high similarity (median, 0.85–0.91; Supplementary Fig. 1), indicating stable semantic grounding while capturing complementary functional perspectives. Based on these findings, we focused on RAG-generated descriptions for subsequent analyses, as they provide functionally diverse yet semantically consistent gene representations while enabling scalable integration of up-to-date biomedical knowledge.

### Identification and interpretation of gene embedding clusters

Using the RAG-function gene embeddings, we grouped genes into 107 clusters using *k*-means clustering with the elbow method (cluster size, 47–420; Fig. 2b; “Methods”). Functionally coherent gene groups were robustly identified, including clusters associated with genomic regulation and adaptation, transcriptional regulation in cellular development, immune regulation and tissue homeostasis, cytoskeletal dynamics, nucleolar protein synthesis, and mitochondrial dysfunction in disease. Additional clusters captured cancer-related regulatory modules, such as gene expression regulation in cancer and oncogenic regulation in cancer, as well as specialized biological processes including cellular vesicle fusion machinery, intracellular movement and disease mechanisms, and neurodegenerative and mitochondrial dysfunction.

To assess functional relevance, we identified the top 10 representative keywords for each cluster using named entity recognition and frequency analysis (Supplementary Data 1). Notably, 104 clusters (97.2%) were characterized by at least one unique keyword (Fig. 2c), highlighting the functional specificity captured by the gene embeddings. To further examine cluster organization, we conducted an ablation analysis to quantify the influence of individual entities on intra-cluster cohesion (“Methods”). Removal of the top 10 representative terms within each cluster resulted in an average decrease of only 0.02% in intra-cluster cosine similarity (range across clusters, 0.004–0.13%; Fig. 2d; baseline distribution in Supplementary Fig. 2). This modest reduction indicates that cluster cohesion is not driven by a small number of dominant keywords, but instead reflects distributed semantic signals across multiple biological concepts embedded within gene descriptions. Such distributed effects are consistent with the inherent complexity and contextual richness of gene function narratives, supporting the use of LLM-based embeddings that can integrate nuanced, multi-concept information beyond what simpler word-level embedding models typically capture.

Interestingly, overlap analysis with Gene Ontology Biological Process (GO-BP) terms (*n* = 4146; Molecular Signatures Database [MSigDB]^3^) showed that 33 clusters (30.8%) had no significant overlap, defined as failing to meet the criteria of Fisher’s exact test with Benjamini-Hochberg correction (*p* < 0.05) and Jaccard index > 0.1, consistent with thresholds used in a recent study^5^. Using a lower Jaccard threshold of 0.05 still resulted in 20 non-overlapping clusters (18.7%). These findings suggest that a subset of embedding-derived clusters may represent functional modules not captured by existing ontology frameworks. These unique clusters were associated with diverse biological themes, including specific functions in cancer, gene regulation and expression, cellular organization and differentiation, and protein or extracellular interactions (Fig. 2b and Supplementary Data 1).

Together, these results establish that LLM-derived embeddings capture structured and meaningful functional organization of genes, providing a foundation for systematic evaluation of gene-gene and gene-function relationships.

### Systematic evaluation of gene-gene functional similarity captured by LLM embeddings across diverse annotation categories

**Fig. 3:**
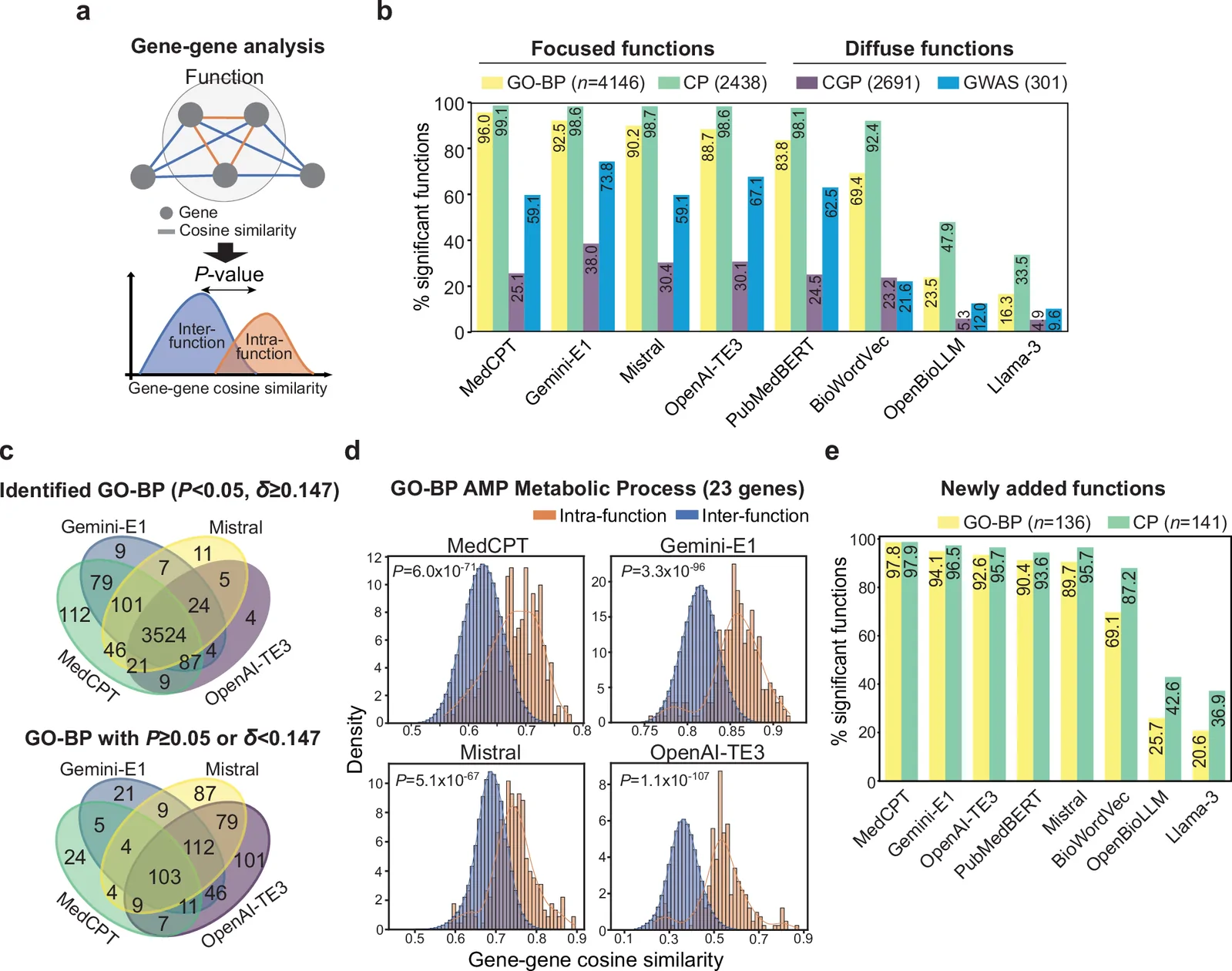
Evaluation of gene-gene functional similarity captured by LLM-derived embeddings. **a** Schematic of gene-gene similarity testing based on intra-function versus inter-function cosine similarity. Gene sets from four functional annotation databases (focused functions: GO-BP and CP; diffuse, multifactorial functions: CGP and GWAS) were analyzed using their corresponding RAG-derived descriptions (RAG-function, RAG-pathway, RAG-perturbation, and RAG-disease, respectively). Statistical significance and effect size were assessed using one-tailed Mann-Whitney U tests and Cliff’s delta *δ*, respectively. **b** Proportion of identified functions (one-tailed Mann-Whitney U test *p* < 0.05 and *δ* ≥ 0.147) captured by each embedding model. **c** Venn diagrams showing overlap of GO-BP terms identified (top; one-tailed Mann-Whitney U test *p* < 0.05 and *δ* ≥ 0.147) and not identified (bottom) by the top four models. Results of CP, CGP, and GWAS are shown in Supplementary Fig. 4. **d** Distribution of gene-gene cosine similarities for the GO-BP term “AMP Metabolic Process” across four top-performing models. Density plots were generated using kernel density estimation with Gaussian kernels. Statistical significance was assessed using one-tailed Mann-Whitney U tests. **e** Performance on GO-BP and CP functions newly added between October 2023 and January 2025, after the literature used for RAG-based gene description generation, showing the proportion of significant functions identified by each embedding model and demonstrating generalizability to recently curated biological processes. Source data are provided as a Source Data file.

We developed a statistical framework to systematically evaluate whether similarities in gene embeddings reflect underlying biological function (Fig. 1 and flowchart in Supplementary Fig. 3). To do this, we used curated functional annotations and corresponding gene sets from the MSigDB and GWAS Catalog^14^. These biological functions span diverse biological contexts across two major categories (Supplementary Table 3): two focused annotation sets, including GO-BP (*n* = 4146), which provide broad gene coverage for fundamental biological functions, and canonical pathways (CP; *n* = 2438), which represent well-annotated, mechanistically established pathways; and two diffuse, context-dependent annotation sets that capture broader, multifactorial functional involvement: chemical and genetic perturbation signatures (CGP; *n* = 2691), which often reflect multi-process cellular responses, and disease-associated genes identified from genome-wide association studies (GWAS; *n* = 301). For each function, we compared the cosine similarity of gene embeddings between functionally related gene pairs (within the same gene set) and unrelated pairs (one gene inside and one outside the set) (Fig. 3a). Statistical comparisons were performed using the Mann-Whitney U test, with significance defined as *p* < 0.05 and non-negligible effects identified by Cliff’s delta (*δ* ≥ 0.147; equivalent to Cohen’s *d* ≥ 0.2, often used to represent small effect).

Beyond OpenAI-TE3, we evaluated embeddings from six additional LLM-based models (Supplementary Table 1), including gemini-embedding-001 (Gemini-E1)^15^, general-purpose open-source LLMs (Llama-3^16^ and Mistral^17^), and biomedical models (PubMedBERT^18^, MedCPT^19^, and OpenBioLLM). As a non-LLM baseline for evaluating the semantic representations captured by LLM-based methods, we included BioWordVec^20^, a state-of-the-art word-level embedding model trained on PubMed and clinical text corpora.

Strikingly, embeddings from MedCPT, Gemini-E1, Mistral, and OpenAI-TE3 captured gene-gene functional relationships across 88.7-96.0% of GO-BP and nearly all CP sets (98.6-99.1%), showing significant separation (one-tailed Mann-Whitney U test *p* < 0.05 with Cliff’s delta *δ* ≥ 0.147; Fig. 3b, c and Supplementary Fig. 4), outperforming the other LLM-based embeddings and BioWordVec by large margins. For example, in the GO-BP term “AMP Metabolic Process,” within-set similarities were significantly higher than between-set comparisons (all *p* < 5.1 × 10^−67^; Fig. 3d). The remaining three LLMs and BioWordVec demonstrated lower performance, achieving 33.5–98.1% in CP sets and 16.3–83.8% in GO-BP sets.

In contrast, performance on the diffuse annotation sets (CGP and GWAS) was substantially lower (25.1–38.0% and 59.1–73.8% for the same four best-performing models in CGP and GWAS, respectively; Fig. 3b and Supplementary Fig. 4a), reflecting the broader and more heterogeneous nature of perturbation responses and disease pathogenesis. For CGP, gene sets often include mixed signals from stress responses, compensatory pathways, or secondary effects that can be highly context-dependent, making them less functionally cohesive than GO-BP or CP sets^21–23^. Similarly, GWAS gene sets often aggregate loci associated with complex traits rather than mechanistically unified functions, resulting in weaker semantic coherence and lower embedding-based separability. Notably, performance generally improved with smaller CGP and GWAS sets (Supplementary Fig. 5), likely because smaller sets represent perturbations and diseases with higher functional specificity. We further performed the analysis using curated gene descriptions from the NCBI Gene database. While comparable performance was observed on GO-BP, CP, and CGP sets, performance on GWAS sets was markedly lower (Supplementary Fig. 6a), supporting the effectiveness of RAG in representing the disease contexts of genes.

To further examine whether the strong performance observed for GO-BP and CP terms could be influenced by information overlap between curated annotations and gene descriptions, we performed an independent evaluation using GO-BP and CP terms added to MSigDB after the publication of the retrieved sentences used for gene description generation in our primary analyses (MSigDB versions 2023.2-2025.1; *n* = 136 and 141, respectively). These newly added terms were therefore independent of embedding generation and prior benchmarking. Applying the same gene-gene similarity framework to these annotations yielded performance comparable to that observed for the earlier datasets (Fig. 3e). These results support the robustness and generalizability of embedding-based functional capture and suggest that the observed separability is not driven solely by annotation overlap.

### Sensitivity of embeddings in capturing gene-function relationships

**Fig. 4:**
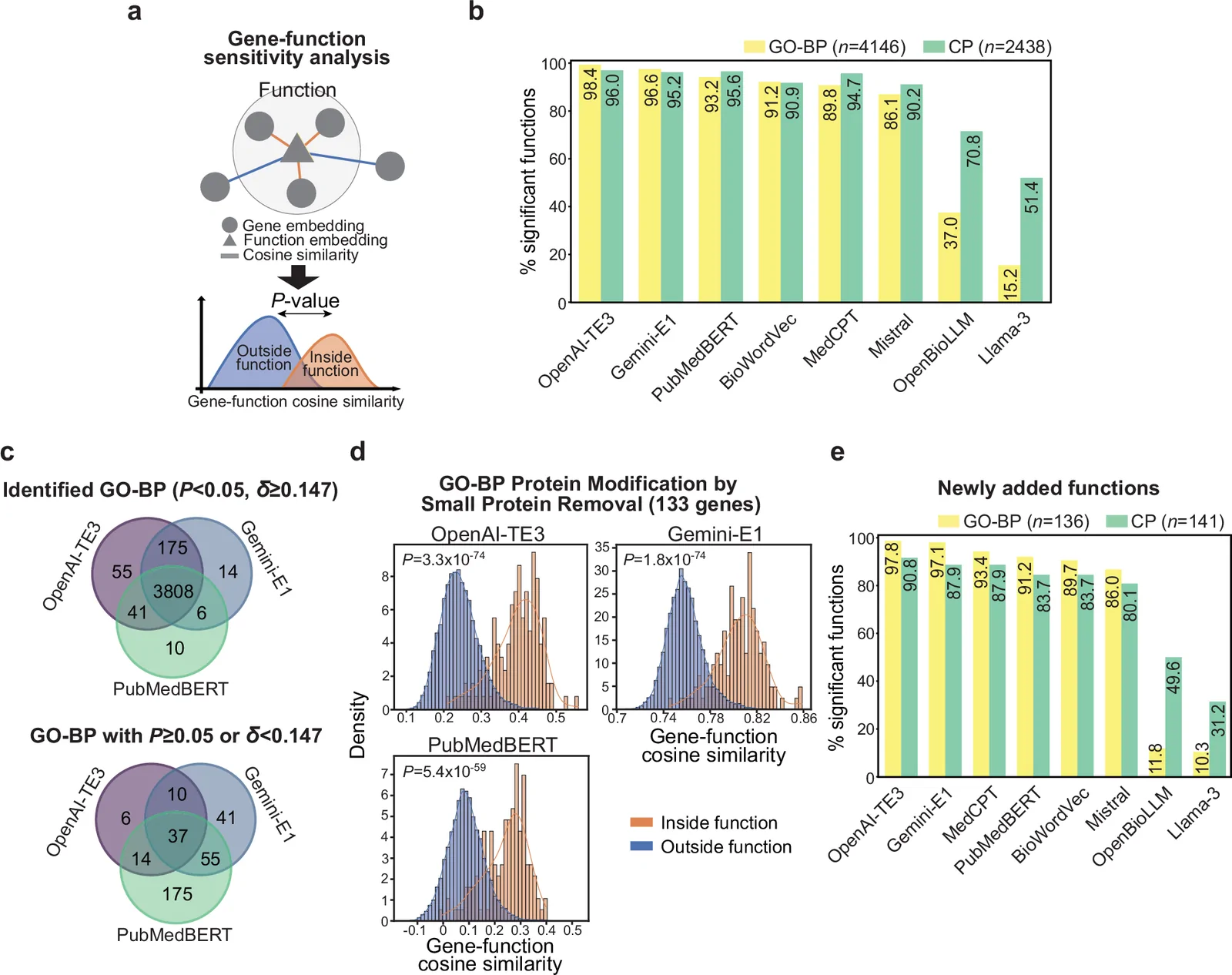
Sensitivity of LLM-derived embeddings in gene-function relationship analysis. **a** Schematic for testing gene-function sensitivity by comparing cosine similarities between gene embeddings in a gene set and the corresponding function embedding (inside function), versus genes outside the set (outside function). Gene embeddings were generated using the corresponding RAG-derived descriptions. Function embeddings were generated from the textual description of functional annotations. Statistical significance was assessed using one-tailed Mann-Whitney U tests (significance threshold: *p* < 0.05 with non-negligible Cliff’s delta *δ* ≥ 0.147). **b** Proportion of GO-BP and CP functions identified as significant (one-tailed Mann-Whitney U test *p* < 0.05 and *δ* ≥ 0.147). Results of CGP and GWAS are presented in Supplementary Fig. 7. **c** Venn diagrams showing GO-BP terms identified (top; one-tailed Mann-Whitney U test *p* < 0.05 and *δ* ≥ 0.147) and not identified (bottom) by the top three models. Results of CP, CGP, and GWAS are shown in Supplementary Fig. 4. **d** Distribution of gene-function cosine similarities for the GO-BP term “Protein Modification by Small Protein Removal” across the three models with sensitivity > 90%. Statistical significance was assessed using one-tailed Mann-Whitney U tests. **e** Performance on functions newly added between October 2023 and January 2025, after the literature used for RAG-based gene description generation. Source data are provided as a Source Data file.

After confirming that embeddings effectively capture local gene-gene functional structure, we next evaluated their ability to align genes with higher-order biological functions, an essential requirement for functional hypothesis testing. In this context, our framework statistically assesses gene-function relationships by measuring the alignment between gene embeddings and predefined function embeddings derived from textual descriptions of GO-BP, CP, CGP, and GWAS annotations (Fig. 4a and Supplementary Fig. 3; see “Methods”). This section focuses on sensitivity, and specificity is addressed in the next section.

Among the evaluated LLMs, OpenAI-TE3, Gemini-E1, and PubMedBERT achieved the highest sensitivity in both GO-BP and CP, with significant gene-function similarities (*p* < 0.05 and *δ* ≥ 0.147) observed in 98.4, 96.6, and 93.2% of GO-BP sets and 96.0, 95.2, and 95.6% of CP sets, respectively (Fig. 4b, c and Supplementary Fig. 4b). For example, in the GO-BP term “Protein Modification by Small Protein Removal,” all three models showed highly significant results (all *p* < 5.4 × 10^−59^; Fig. 4d). Gene embeddings generated from RAG descriptions outperformed those generated from NCBI gene descriptions (Supplementary Fig. 6b). Similar to the gene-gene analysis, sensitivity in the diffuse annotation sets was lower across all models (Supplementary Fig. 7). OpenAI-TE3, Gemini-E1, and PubMedBERT again performed best in CGP (24.4–36.1%) and GWAS (77.4–82.4%). This reduced performance likely reflects the greater context dependency of CGP and GWAS descriptions, which often emphasize perturbation types and experimental conditions rather than shared biological roles, as well as the multifactorial and mechanistic heterogeneity underlying disease pathogenesis. Of note, BioWordVec ranked third to fourth across all four functional categories, reflecting its ability to capture the frequent co-occurrence of genes with their functional and disease-related terms in biomedical literature and clinical text. When applied to GO-BP and CP terms newly added to MSigDB (versions 2023.2-2025.1), OpenAI-TE3 maintained comparably strong performance across these newly curated terms (97.8% for GO-BP and 90.8% for CP; Fig. 4e).

### Specificity of LLM embeddings in representing gene-function relationships

**Fig. 5:**
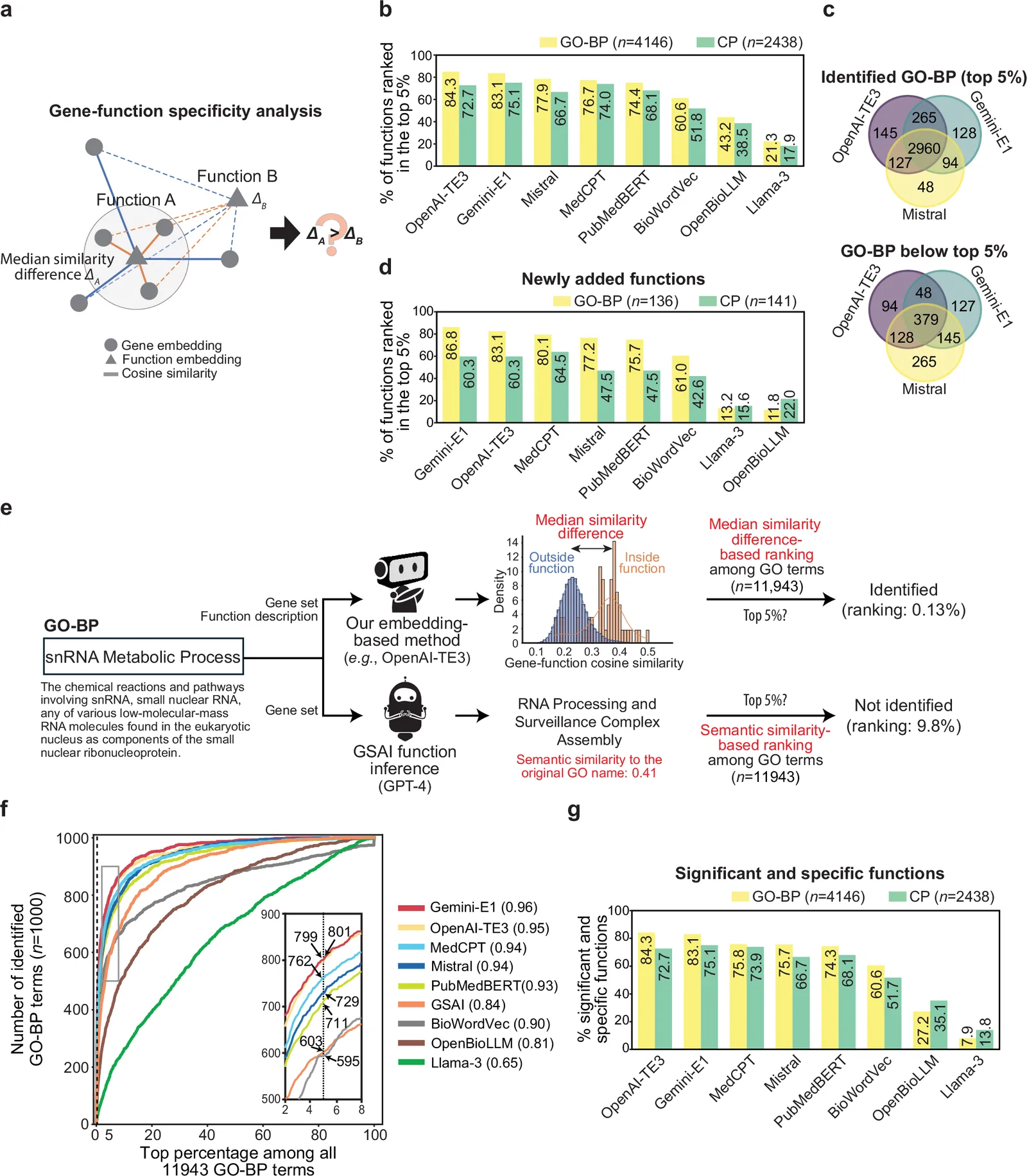
Specificity of gene-function relationships captured by LLM-derived embeddings. **a** Schematic illustrating evaluation of function specificity. For each function A and its associated gene set, we computed the median similarity difference (Δ_*A*_), defined as the difference between the median cosine similarity of genes to their corresponding function embedding and the median cosine similarity of other genes to the same function embedding. Δ_*A*_ was then compared with an analogous measure for an unrelated function B (Δ_*B*_). A gene-function relationship was considered specific if Δ_*A*_ ranked within the top 5% of all tested functions. **b** Proportion of GO-BP and CP terms identified as specific, defined by ranking within the top 5% of tested functions. Results of CGP and GWAS are presented in Supplementary Fig. 7. **c** Venn diagrams of GO-BP terms identified (top 5%) or not identified (below top 5%) by the top three models. Results for CP, CGP, and GWAS are shown in Supplementary Fig. 4. **d** Performance on GO-BP and CP functions added between October 2023 and January 2025, after the literature used for RAG-based gene description generation. **e** Comparison with the baseline model GSAI, which infers a function name for a query gene set using GPT-4 and ranks all 11,943 GO-BP terms by semantic similarity to the predicted function. The robot icon used throughout this study was adapted from ref.^9^. under the CC BY 4.0 license. **f** Specificity performance curves for the embedding models and GSAI, evaluated using the same 1000 GO-BP terms selected as query sets in the GSAI study, across varying percentile cutoffs for specificity. Values in parentheses indicate the area under the curve for each method. The dashed line indicates the 5% cutoff used in this study, consistent with the original GSAI analysis. Models in the legend are ordered by performance at this cutoff. **g** Proportion of functions meeting both sensitivity and specificity criteria, integrating results from Figs. 4b and 5b (one-tailed Mann-Whitney U test *p* < 0.05 and *δ* ≥ 0.147, and top 5% specificity ranking) for GO-BP and CP terms across embedding models. Source data are provided as a Source Data file.

Given the hierarchical and interrelated nature of biological functions, capturing gene-function specificity is critical; *i.e*., determining whether an observed gene-function relationship is uniquely associated with a particular function or broadly shared across others (Fig. 5a). We defined a relationship as specific if the target function ranked within the top 5% among all functional annotations (*e.g*., all GO-BP terms) when tested against the same gene set (“Methods”), consistent with a previously established threshold to assess semantic similarity among GO terms^5^. OpenAI-TE3 performed best, identifying specific gene-function relationships in 84.3% of GO-BP sets and 72.7% of CP sets (Fig. 5b, c and Supplementary Fig. 4c), trailed by Gemini-E1 (83.1% and 75.1%) and Mistral (77.9% and 66.7%). Relative to embeddings generated from NCBI gene descriptions, embeddings generated from RAG descriptions showed improved performance for GO-BP and comparable performance for CP sets (Supplementary Fig. 6c). Specificity in CGP sets remained low across models (Supplementary Fig. 7). When applied to terms newly added to MSigDB, the specificity of OpenAI-TE3 and Gemini-E1 remained high for GO-BP (86.8% and 83.1%, respectively; Fig. 5d), whereas performance for CP was moderately reduced (60.3%).

To further benchmark specificity under a standardized evaluation framework, we compared the embedding methods to Gene Set AI (GSAI)^5^, which evaluates the semantic specificity of function names predicted by GPT-4 for 1000 GO-BP gene sets against the full GO-BP hierarchy (11,943 terms; “Methods”, Supplementary Table 3, and illustration in Fig. 5e). Five embedding models, led by Gemini-E1 (80.1%) and OpenAI-TE3 (79.9%), achieved substantially higher specificity rates (71.1-80.1%) than GSAI (60.3%) (Fig. 5f and Supplementary Data 2). Across varying specificity thresholds, the five embedding-based models consistently outperformed GSAI, again with Gemini-E1 and OpenAI-TE3 achieving the highest areas under the curve (0.95-0.96; Fig. 5f) compared to GSAI (0.84).

Gene-function pairs identified by our framework significantly overlapped with those captured by GSAI (one-tailed Fisher’s exact test, *p* = 7.2 × 10^−33^). Among the 47 sets missed by our method, 24 (51.1%) were within the borderline range (top 10% of ranks). Importantly, our framework uniquely identified 243 sets. For example, “snRNA Metabolic Process” ranked ninth of all 11,943 GO terms (0.13%) using our method (Fig. 5e and Supplementary Data 2), whereas GSAI’s inferred function “RNA Processing and Surveillance Complex Assembly” ranked only in the top 9.8%.

Notably, when both sensitivity and specificity were jointly considered (*p* < 0.05, *δ* ≥ 0.147, and top 5% specificity ranking), the best-performing model, OpenAI-TE3, captured 84.3% of GO-BP and 72.7% of CP terms (Fig. 5g). Taken together, these findings further demonstrate that embedding-based gene-function alignment generalizes to recently curated biological functions and supports its applicability to emerging biological functions and pathways. Given its consistently strong performance across both sensitivity and specificity analyses, we selected OpenAI-TE3 for subsequent analyses of LLM-inferred de novo gene-set function hypotheses.

### Statistical evaluation of LLM-inferred de novo gene-set function hypotheses under contamination

While the preceding analyses established the sensitivity and specificity of embedding-based gene-function alignment using curated annotations, LLMs are increasingly used to infer shared functions directly from gene lists^5,6^, including the state-of-the-art GSAI method. In such workflows, models such as GPT-4o generate functional hypotheses with confidence scores derived from prompting strategies rather than formal statistical testing^24^, limiting their ability to distinguish true biological signal from noise. Our framework addresses this gap by enabling statistical evaluation of LLM-generated gene function hypotheses. Before applying it to real-world experimental gene lists, we first assessed robustness using curated GO-BP gene sets subjected to systematic contamination. By progressively introducing random genes into known functional sets, we simulated varying signal-to-noise ratios commonly observed in high-throughput datasets and evaluated whether statistical significance appropriately tracked the underlying biological signal.

**Fig. 6:**
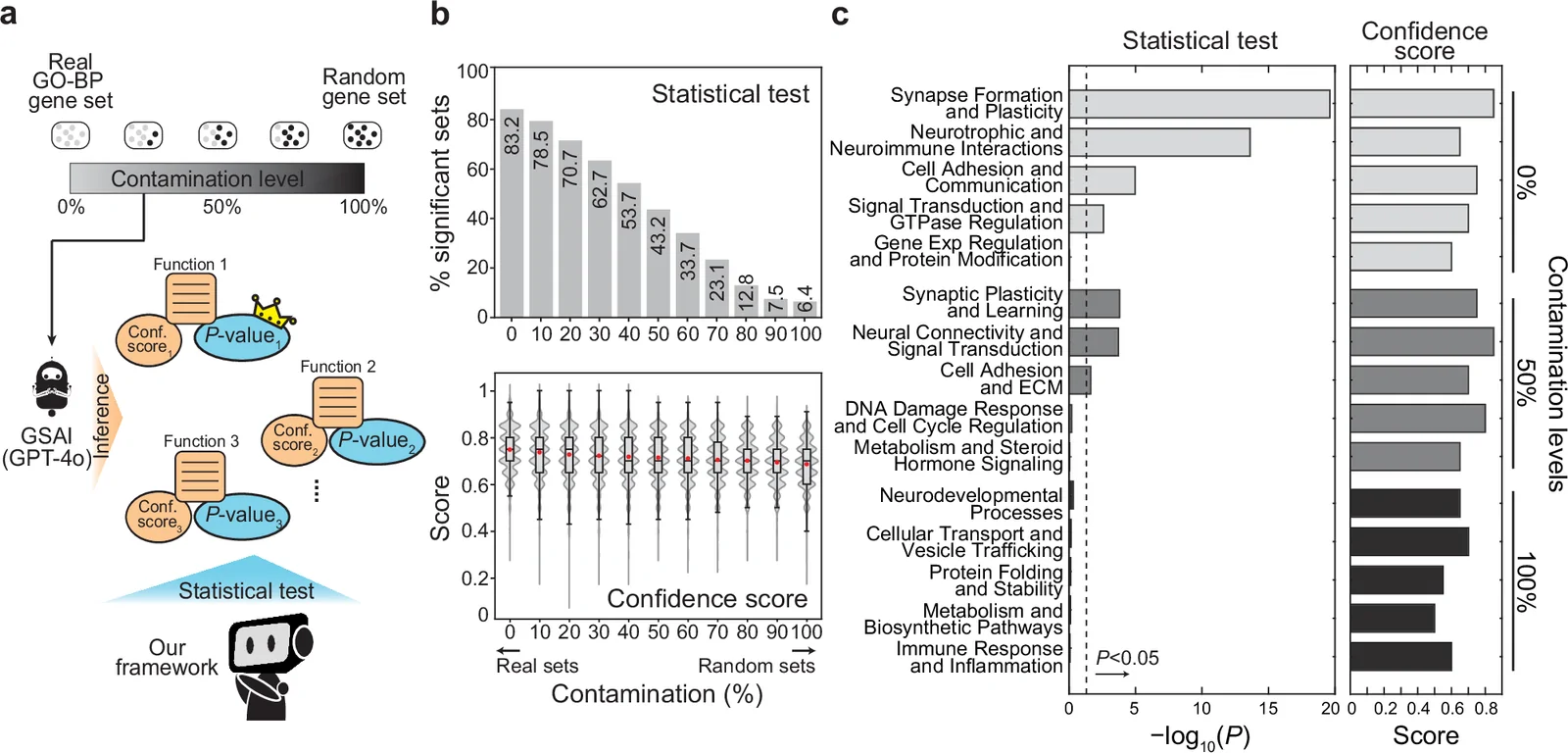
Embedding-based hypothesis testing of LLM-inferred de novo functions under contamination. **a** Contamination analysis framework for testing LLM-inferred de novo functional hypotheses. Genes within each of the 4146 GO-BP gene sets were progressively replaced with randomly selected genes to generate contaminated sets. GSAI (using a modified GPT-4o-based prompt; see Supplementary Method 1) was applied to each set to predict up to five functions, each accompanied by an LLM-inferred confidence score. The predicted functions were then statistically evaluated against the corresponding contaminated gene set using our gene-function sensitivity analysis framework. Conf. score, confidence score. **b** Proportion of significant results from the proposed framework (upper panel; with one-tailed Mann-Whitney U test *p* < 0.05 and Cliff’s delta *δ* ≥ 0.147) and GSAI confidence scores (lower) across varying contamination levels. For each level, the proportion and score were computed by aggregating up to 20,730 predicted gene functions (up to five predictions per GO-BP term, across 4146 terms; actual sample sizes, 20,548-20,671). In the lower panel, violin plots show the confidence score distribution, and the center line and red dot denote the median and mean, respectively; box bounds indicate the 25^th^ and 75^th^ percentiles; and whiskers extend to the farthest data points within 1.5 × the interquartile range. **c** Performance for the GO-BP term “Postsynaptic Specialization Organization” at 0, 50, and 100% contamination, showing one-tailed Mann-Whitney U test *p*-values from our framework in comparison with confidence scores from GSAI. Exp, expression; ECM, extracellular matrix. Source data are provided as a Source Data file.

We implemented the GSAI model using GPT-4o to predict up to five functions (*i.e*., hypotheses) for each gene list, along with corresponding textual descriptions and confidence scores ranging from 0 to 1 (“Methods”, Supplementary Method 1, and Supplementary Fig. 8). We then computed cosine similarity between the input gene list and each function embedding derived from GSAI-generated text. To assess performance, we conducted a contamination test across GO-BP gene sets (Fig. 6a). The rationale is that increasing contamination (*i.e*., replacing true genes with random genes) should progressively weaken the functional signal and reduce statistical significance. Notably, GSAI confidence scores declined only modestly across contamination levels, from an average of 0.75 at 0% to 0.69 at 100% contamination (Fig. 6b), limiting the ability to distinguish between biologically meaningful and noisy gene lists. Moreover, these scores exhibited a coarse, discretized distribution with peaks concentrated around common thresholds (*e.g*., 0.70 or 0.75; Fig. 6b and Supplementary Fig. 9), reflecting their qualitative rather than calibrated nature.

In contrast, our framework revealed clear differentiation across contamination levels (Fig. 6b): the proportion of significant gene-function associations (*p* < 0.05 and *δ* ≥ 0.147) declined markedly from 83.2% at 0% contamination to 43.2% at 50%, and further to 6.4% at 100%. For example, for the GO-BP term “Postsynaptic Specialization Organization,” GSAI generated five hypotheses for the true gene set with varying confidence levels (0.60-0.85), including “Synapse Formation and Plasticity” (confidence = 0.85; embedding-based *p* = 2.5 × 10^−20^ and *δ* ≥ 0.147; Fig. 6c and Supplementary Data 3) and other related signaling pathways and processes such as neuroimmune interaction and cell adhesion. Notably, despite a moderate confidence score (0.60), the broader hypothesis “Gene Expression Regulation and Protein Modification” was not statistically significant under our framework, indicating that embedding-based statistical testing can distinguish mechanistically coherent functions from more generic biological processes. When 50% of the genes were replaced with random ones, “Synaptic Plasticity and Learning” and “Neural Connectivity and Signal Transduction” were still predicted with high confidence (0.75 and 0.85), yet their statistical association decreased markedly (*p* = 1.6 × 10^−4^ and 1.9 × 10^−4^, respectively; and both *δ* ≥ 0.147). At full contamination (100%), GSAI continued to generate five terms (confidence: 0.50-0.70), yet none were statistically significant using our method. These results demonstrate that GSAI confidence scores alone are less responsive to changes in underlying biological signal strength, whereas our statistical framework captures the progressive loss of functional coherence as the signal is diluted by random genes. Accordingly, the embedding-based framework provides the statistical resolution necessary to distinguish biologically meaningful from noisy gene lists, thereby improving the reliability of LLM-guided de novo functional discovery and enabling quantitative hypothesis testing.

### Evaluation on LLM-inferred functions from experimentally informed gene systems

Following controlled contamination experiments, we next evaluated our framework using experimentally informed, computationally integrated gene systems from the Nested Systems in Tumors (NeST) resource (*n* = 50)^25^, a collection of protein assembly modules used in recent LLM benchmarking studies^5,6^. Unlike curated ontology terms, NeST gene lists represent context-specific, network-derived protein assemblies informed by large-scale experimental interaction data and therefore provide a stringent and biologically realistic test of hypothesis-free functional inference.

**Fig. 7:**
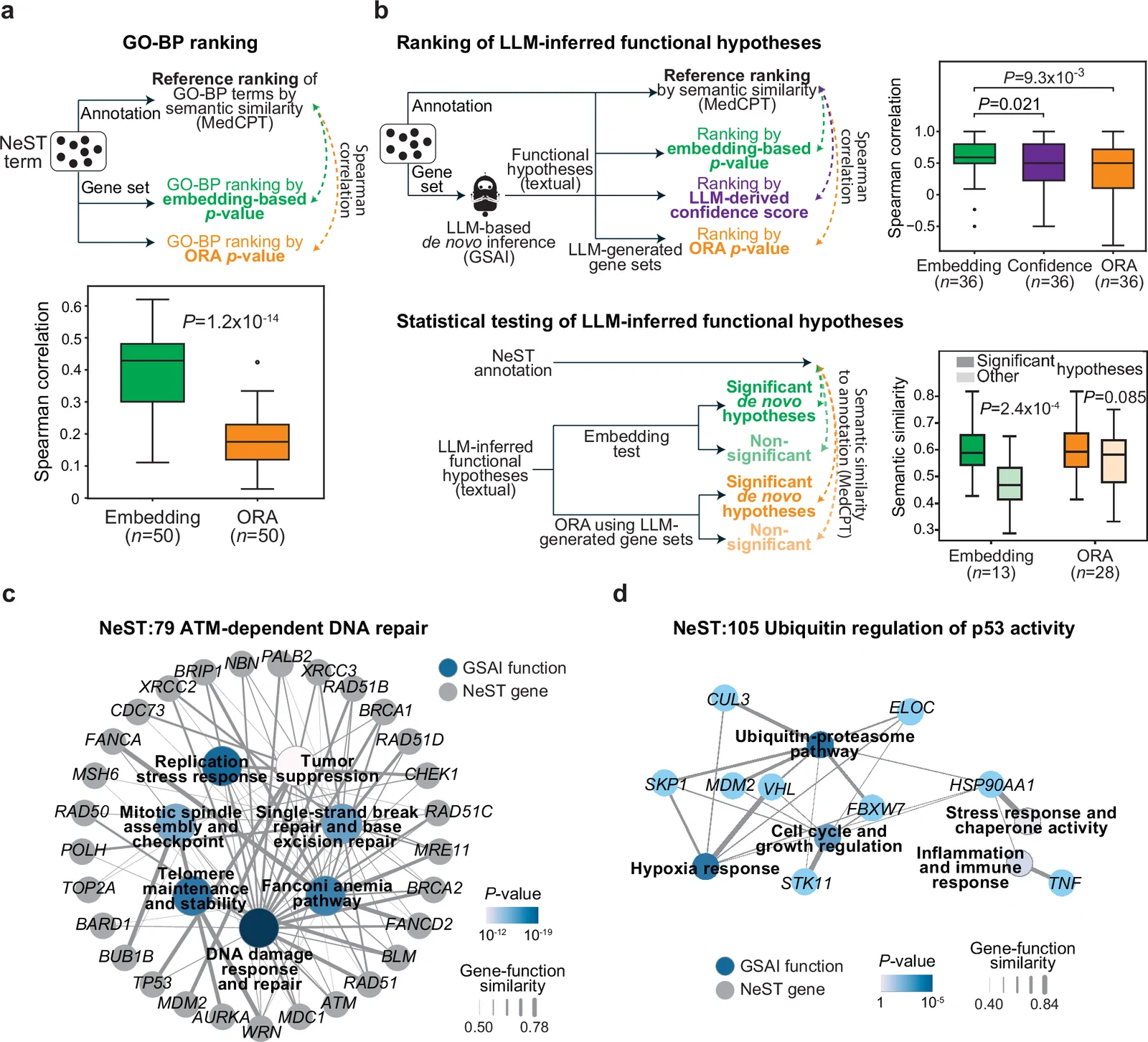
Real-world evaluation of embedding-based hypothesis testing using NeST protein systems. **a** Evaluation of GO-BP term prioritization using 50 NeST gene lists. GO-BP terms were ranked either by statistical significance derived from our embedding-based gene-function sensitivity analysis (Fig. 4a) or by overrepresentation analysis (ORA), and compared against a semantic reference ranking derived from MedCPT similarity to the corresponding NeST annotations. Spearman's rank correlations between each ranking strategy and the semantic reference ranking are shown. Statistical significance was assessed by a one-tailed Wilcoxon signed-rank test. The center line denotes the median, box bounds indicate the 25^th^ and 75^th^ percentiles, and whiskers extend to the farthest data points within 1.5 × the interquartile range. **b** Evaluation of ranking and statistical testing of LLM-inferred de novo functional hypotheses generated by GSAI. Upper panel: Spearman correlations between semantic reference rankings and hypothesis prioritization based on embedding-based statistical testing, GSAI-derived confidence scores, and ORA using GPT-4o-generated gene sets corresponding to the inferred textual functions across NeST gene lists. NeST systems with fewer than three inferred functional hypotheses were excluded from analysis. Lower panel: Semantic similarity to the corresponding NeST annotations for hypotheses classified as significant versus non-significant under the embedding-based framework and ORA. For each NeST system, the mean MedCPT similarity to the corresponding NeST annotation was calculated separately for the two groups. Only NeST systems containing at least one hypothesis in each group were included. In both panels, statistical significance was assessed using a one-tailed Wilcoxon signed-rank test. Box plot elements are defined as in the previous panel. **c** Gene-function network for the NeST system “ATM-Dependent DNA Repair” (NeST:79). GSAI-inferred functions are connected to component genes based on embedding-derived cosine similarity. Node shading reflects the embedding-based statistical significance (-log10 *p*-value from a one-tailed Mann-Whitney U test), and edge width represents gene-function cosine similarity (edges with similarity < 0.50 are not shown). **d** Gene-function network for the NeST system “Ubiquitin Regulation of p53 Activity” (NeST:105). Edges with cosine similarity < 0.40 are not shown. Statistical significance was assessed using a one-tailed Mann-Whitney U test. Source data are provided as a Source Data file.

We first analyzed each NeST gene list against GO-BP terms using our gene-function sensitivity framework (Fig. 4a) and compared it with conventional ORA based on Fisher’s exact test, reflecting standard functional annotation workflows for high-throughput data (Fig. 7a). To define a reference ranking, we calculated MedCPT-based semantic similarity between GO-BP term names and the corresponding NeST annotation using the MedCPT Query Encoder model, following a recent study^6^ (“Methods”). This MedCPT Query Encoder is distinct from the MedCPT Article Encoder evaluated in previous sections. GO-BP rankings prioritized by the *p*-value obtained from our embedding-based method showed substantially higher agreement with this reference ordering than those obtained by ORA (median Spearman correlation across 50 NeST lists, 0.43 vs. 0.18; one-tailed Wilcoxon signed-rank test *p* = 1.2 × 10^−14^; Fig. 7a), indicating improved recovery of biologically relevant functional themes.

We next evaluated de novo functional inference. Unlike the previous evaluation, which directly assessed enrichment of predefined GO-BP terms from the NeST gene lists, this analysis evaluated free-text inferred functional concepts generated by an LLM (Fig. 7b). For each NeST gene list, we applied GSAI to generate up to twenty candidate functional hypotheses, allowing broader exploration of potential functions in these experimentally defined systems. On average, GSAI predicted 4.6 functions per NeST gene list (range, 1–15; Supplementary Data 4). Each inferred function was then statistically evaluated using our embedding-based gene-function sensitivity framework. For comparison, we implemented ORA using GPT-4o-generated gene sets corresponding to each inferred function, because these de novo textual hypotheses may not directly correspond to existing curated gene sets or ontology terms, thereby enabling direct comparison under a matched inference setting (Fig. 7b, upper panel; “Methods”). Across NeST gene lists, our method more effectively prioritized inferred hypotheses, achieving higher Spearman correlation with the semantic reference ranking (median = 0.59 across 36 NeST lists) than either GSAI confidence scores or ORA (one-tailed Wilcoxon signed-rank test *p* = 0.021 and 9.3 × 10^−3^, respectively; Fig. 7b). Importantly, functions classified as significant by our framework (*p* < 0.05 and *δ* ≥ 0.147) showed significantly higher MedCPT semantic similarity to the corresponding NeST annotations than non-significant predictions (one-tailed Wilcoxon signed-rank test *p* = 2.4 × 10^−4^; *n* = 13; Fig. 7b, lower panel), whereas ORA did not effectively distinguish biologically coherent hypotheses from less supported ones (*p* = 0.085; *n* = 28).

### Representative case studies from NeST systems

To illustrate whether embedding-based statistical testing can alter functional prioritization and mechanistic interpretation within controlled experimental systems, we examined two representative NeST gene lists representing a highly coherent DNA repair module and a more heterogeneous regulatory module. For the protein system “ATM-Dependent DNA Repair” (NeST:79; 29 genes), GSAI produced seven closely related hypotheses spanning core DNA damage signaling and repair processes (confidence scores, 0.50-0.95; Fig. 7c and Supplementary Data 4). These hypotheses were well aligned with the system’s gene composition, which includes canonical ATM-mediated DNA repair components, such as *ATM*, *TP53*, *BRCA1/2*, and *CHEK1* (Fig. 7c). Using our framework, all seven inferred functions were significantly associated with the NeST gene set, including hypotheses that received relatively lower LLM confidence, indicating that statistical testing can recover biologically coherent functions beyond confidence ranking alone. In contrast, ORA statistics and LLM confidence scores showed weaker prioritization relative to the semantic reference ranking, with Spearman correlations of 0.32 and 0.25, respectively, compared with 0.57 for the embedding-based method (side-by-side comparisons are provided in Supplementary Data 4). To gain further molecular insight, we constructed a gene-function network based on embedding-derived cosine similarities (Fig. 7c). The network supported mechanistic coherence: inferred DNA repair-related functions formed a connected module with multiple strong gene-function links, consistent with coordinated ATM-mediated signaling and downstream repair pathways captured by this experimentally defined system.

In contrast, the NeST system “Ubiquitin Regulation of p53 Activity” (NeST:105; 9 genes) yielded five hypotheses spanning ubiquitin signaling, growth control, stress responses, and immune-related processes (Fig. 7d). Our statistical testing supported three hypotheses, “Ubiquitin-Proteasome Pathway,” “Cell Cycle and Growth Regulation,” and “Hypoxia Response,” whereas “Stress Response and Chaperone Activity” and “Inflammation and Immune Response” were not significant by our criteria (*p* < 0.05 and *δ* ≥ 0.147; Fig. 7d and Supplementary Data 4). This prioritization shifted interpretation toward a more coherent ubiquitin-regulatory and growth-control framework while down-weighting broader stress- and immune-related hypotheses. This separation was consistent with gene-level connectivity in the network. The significant functions were linked through a coherent set of ubiquitin and growth-control genes such as *MDM2*, *VHL*, *FBXW7*, *CUL3*, *SKP1*, *ELOC*, and *STK11*, whereas the non-significant terms showed comparatively weaker and more peripheral connections, for example via *HSP90AA1* and *TNF* (Fig. 7d). The significant terms also exhibited stronger similarity to the corresponding NeST annotation, with median similarities of 0.60 for significant hypotheses (*n* = 3) versus 0.44 for non-significant hypotheses (*n* = 2), although this comparison was underpowered and did not reach statistical significance in a one-tailed Mann-Whitney U test (*p* = 0.20). In contrast, ORA classified four terms as significant but failed to prioritize the most mechanistically central term, “Ubiquitin-Proteasome Pathway,” highlighting its limited specificity in this context.

Together, these examples illustrate that embedding-based hypothesis testing preserves mechanistic signal in well-defined modules while down-weighting broader functional descriptions that are plausible but less strongly supported by the underlying gene list. These case studies further demonstrate how embedding-based statistical testing can alter functional prioritization and mechanistic interpretation relative to conventional enrichment analysis and qualitative LLM confidence scoring within experimentally derived systems. Combined with the contamination analysis, these controlled evaluations demonstrate that embedding-based hypothesis testing bridges qualitative LLM inference and quantitative functional genomics, enabling rigorous interpretation of experimentally defined protein assemblies not yet incorporated into annotation databases.

### DEGEmbedR implementation for de novo functional annotation of DEGs

**Fig. 8:**
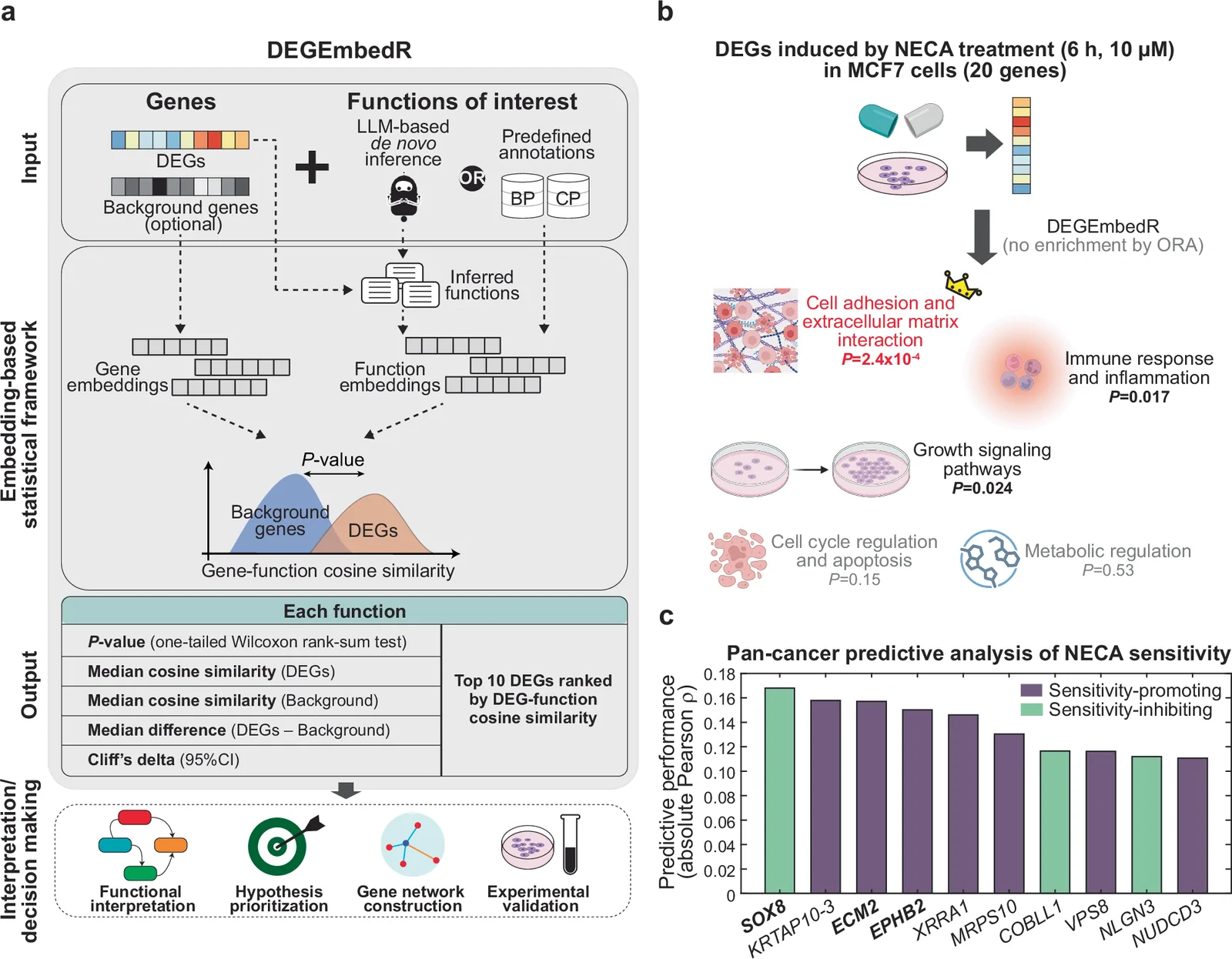
DEGEmbedR implementation and case study for embedding-based interpretation of differentially expressed gene (DEG) signatures. **a** Overview of the R package DEGEmbedR workflow for embedding-based statistical interpretation of DEG signatures using either LLM-inferred de novo functional hypotheses generated by our re-implementation of GSAI or predefined functional annotations. DEGEmbedR generates gene and function embeddings, performs the gene-function sensitivity analysis presented in this study (Fig. 4a), and statistically evaluates prioritized functional associations relative to user-provided or built-in background genes. The workflow is designed to support downstream biological interpretation, hypothesis prioritization, and decision-making in routine DEG analysis workflows. **b** Application of DEGEmbedR to a NECA treatment-derived DEG signature in MCF7 cells from the LINCS dataset previously reported to lack significant annotation by ORA. Embedding-based statistical testing prioritized extracellular matrix interaction-, inflammatory-, and growth signaling-related functional hypotheses, in contrast to broader and nonspecific functions, including cell cycle and metabolic regulation. Biological illustrations in this panel were created with BioRender: Created in BioRender. Guo, Y. (2026) https://BioRender.com/w0ujs8p. **c** Pan-cancer analysis of gene expression predictors of NECA sensitivity using the Cancer Dependency Map (DepMap) Portal. The top 10 predictor genes were obtained from the portal and ranked by absolute Pearson correlation. Genes associated with extracellular interaction discussed in the Results section are highlighted in bold. Source data are provided as a Source Data file.

To facilitate broader application in functional genomics, we developed an open-source R package, DEGEmbedR^26^, that implements our embedding-based framework for functional annotation of gene lists, including DEGs derived from high-throughput experiments. As illustrated in Fig. 8a, DEGEmbedR supports both hypothesis-free and annotation-guided analyses by enabling de novo functional hypothesis generation followed by embedding-based statistical evaluation within our framework, as well as direct testing against curated functional annotations such as GO-BP and CP. By integrating LLM-based functional inference with quantitative statistical testing, the package provides a practical workflow for interpreting DEG signatures, particularly in settings where conventional enrichment approaches yield limited or no informative annotations. The resulting prioritized functional associations can subsequently support downstream functional interpretation, hypothesis prioritization, gene network construction, and experimental validation.

As a use case, we applied DEGEmbedR to a set of 20 DEGs identified from 5’-N-ethylcarboxamidoadenosine (NECA; an adenosine receptor agonist) treatment in MCF7 breast cancer cells in the Library of Integrated Network-Based Cellular Signatures (LINCS) dataset^27^ previously reported to lack significant functional annotation by overrepresentation analysis^5^. Indeed, our reanalysis of this set against 4146 GO-BP terms yielded no significant overlap (significance defined as Fisher’s exact test with Benjamini-Hochberg correction *p* < 0.05). In contrast, DEGEmbedR generated and quantitatively prioritized multiple biologically plausible de novo functional hypotheses (Fig. 8b). Among the five candidate functions inferred by the LLM, embedding-based statistical testing strongly prioritized “Cell adhesion and extracellular matrix interaction” (*p* = 2.4 × 10^-4^), followed by “Immune response and inflammation” (*p* = 0.017) and “Growth signaling pathways” (*p* = 0.024), whereas “Cell cycle regulation and apoptosis” and “Metabolic regulation” were not significant (*p* = 0.15 and 0.53, respectively). This ranking substantially altered the mechanistic interpretation of the perturbation relative to evaluating all candidate functions equivalently or relying solely on LLM confidence scores, which, similar to our previous analyses, ranged narrowly between 0.60 and 0.75. Specifically, the embedding-based prioritization shifted the interpretation from a broad, nonspecific cancer response toward extracellular matrix remodeling and inflammatory signaling, processes strongly associated with adenosine signaling in cancer. Consistent with this interpretation, extracellular adenosine signaling has been implicated in immune suppression, angiogenesis, tumor invasion, and microenvironmental remodeling across multiple solid tumors^28,29^. In contrast, apoptosis and metabolic rewiring, although broadly associated with cancer biology, are less consistently linked to acute NECA responses in breast cancer models. Importantly, this prioritization would directly influence downstream experimental design by favoring migration and invasion assays, extracellular matrix remodeling analyses, cytokine profiling, and receptor subtype interrogation over apoptosis- or metabolism-focused experiments.

Notably, independent pharmacogenomic analysis across pan-cancer cell lines using the Cancer Dependency Map (DepMap) data resources further supported this interpretation. Among the top gene expression features most predictive of NECA sensitivity were extracellular interaction- and cell-state-associated genes, including *ECM2*, *EPHB2*, and *SOX8* (Fig. 8c). *ECM2* encodes extracellular matrix protein 2, whereas *EPHB2* has established roles in tissue organization, migration, and tumor progression^30^. Higher expression of *ECM2* and *EPHB2* predicted stronger NECA response, consistent with the extracellular matrix and adhesion-related functions prioritized by DEGEmbedR. In contrast, *SOX8*, a developmental transcription factor associated with lineage plasticity and stem-like programs in multiple cancers^31^, showed the opposite association with NECA sensitivity. This pattern suggests that NECA response may not simply be linked to a generalized aggressive or mesenchymal-like cellular state, but instead reflects a more specific extracellular interaction and receptor-signaling context. The convergence of multiple independently derived response-associated features on extracellular interaction biology provides orthogonal support for the biologically coherent and mechanistically informative hypotheses prioritized by DEGEmbedR.

More broadly, this example illustrates how embedding-based statistical prioritization can support downstream functional interpretation, hypothesis prioritization, and experimental design in practical DEG analysis workflows (Fig. 8a). Thus, beyond benchmarking semantic similarity, DEGEmbedR provides a practical decision-support layer that quantitatively distinguishes strongly supported mechanistic hypotheses from broader but weakly supported alternatives, a capability difficult to achieve using conventional overlap-based enrichment approaches when no curated annotation reaches significance.

## Discussion

This study establishes a statistical framework that enables quantitative hypothesis testing of gene functions inferred from gene lists using LLM-derived embeddings. Emerging gene-set-free LLM approaches can generate functional summaries, but they lack a principled mechanism to determine whether those inferred functions are statistically supported by the input genes. By systematically evaluating both gene-gene and gene-function relationships across diverse functional categories, we show that modern embedding models, including OpenAI-TE3, Gemini-E1, and the broadly used biomedical model MedCPT, provide a robust quantitative representation of biological function. Our ablation analyses further reveal that embedding structure reflects distributed semantic information across gene descriptions, improving the interpretability of cluster organization. Together, these findings establish embedding-based statistical testing as a scalable quantitative foundation for functional genomics.

The practical importance of this framework becomes evident when applied to LLM-based gene function inference methods such as GSAI. Although GSAI represents an important advance in generating de novo and interpretable functional summaries, its reliance on heuristic confidence scores limits its ability to distinguish a strong biological signal from noise. Our contamination experiments and evaluations on experimentally derived NeST systems demonstrate that confidence scores alone do not consistently track underlying functional coherence. In contrast, embedding-based statistical testing directly evaluates whether an inferred function is quantitatively supported by the gene set and prioritizes hypotheses accordingly. By pairing generative LLM hypothesis production with formal statistical validation, this framework enhances rigor and reproducibility in gene-set-free functional analysis.

In addition to complementing LLM-based inference models, our framework addresses important limitations of conventional ORA. ORA relies on predefined gene sets and tests for enrichment against curated annotations, making it well-suited for hypothesis-driven analyses but less adaptable to de novo functional predictions. In our evaluation of NeST systems, ORA often identified broader functional terms as significant yet showed weaker prioritization relative to semantic reference rankings and, in some cases, failed to highlight the most mechanistically central functions. This limitation arises because enrichment statistics depend primarily on gene overlap counts and do not account for graded semantic alignment between gene descriptions and functional concepts. By contrast, embedding-based gene-function testing evaluates continuous similarity structure, enabling more refined discrimination among closely related hypotheses. As a result, our framework preserves mechanistic signal in coherent modules while down-weighting plausible but weakly supported functional descriptions, providing improved calibration in realistic experimental settings.

A practical example of this capability is provided by the DEGEmbedR analysis of the LINCS NECA perturbation signature. In this setting, conventional ORA failed to identify significant enrichment, while LLM-only inference generated multiple biologically plausible functional hypotheses without clear quantitative prioritization. Embedding-based statistical prioritization instead highlighted extracellular matrix remodeling and inflammatory signaling as the most strongly supported processes, shifting the interpretation away from a broad nonspecific cancer-response framework. Such prioritization could guide downstream experimental design toward migration and invasion assays, extracellular matrix remodeling analyses, and cytokine profiling. Importantly, DEGEmbedR was developed as an open-source, user-friendly R package designed for straightforward integration into standard DEG analysis workflows alongside existing enrichment approaches. Collectively, these findings demonstrate that embedding-based statistical testing can provide a practical decision-support framework for functional genomics by quantitatively prioritizing mechanistically coherent LLM-derived hypotheses over broader but weakly supported alternatives, particularly in settings where conventional annotation-based enrichment approaches provide limited guidance.

Beyond benchmarking embedding models, our results highlight the advantages of using retrieval-augmented gene descriptions as a flexible substrate for functional representation. Compared with structured summaries such as those from NCBI, retrieval-based descriptions can dynamically incorporate recent literature and capture diverse functional facets of a gene across pathways, perturbations, and disease contexts. In our analyses, embeddings generated from retrieval-based descriptions achieved strong performance in both gene-gene and gene-function evaluations, including for new GO-BP and CP terms independent from retrieval. This suggests that embedding-based representations are not narrowly tied to specific curated ontologies but instead reflect broader semantic structure encoded in biomedical text. Such flexibility is particularly important for hypothesis-free settings, where newly characterized genes or emerging biological processes may not yet be fully incorporated into structured databases.

Our analyses also revealed differential behavior across functional categories. Embeddings consistently captured relationships within focused ontology- and pathway-based annotations such as GO-BP and CP, whereas performance was more variable for perturbation-derived and disease-associated sets such as CGP and GWAS. Rather than representing a limitation of the embedding framework, this pattern likely reflects the inherently context-dependent and multifactorial nature of perturbation responses and complex diseases, which frequently involve dynamic interactions among multiple downstream pathways rather than a single cohesive, context-agnostic biological process. In such settings, functional interpretation depends strongly on experimental context, cell type, temporal state, and environmental conditions. These observations highlight an important future opportunity for LLM- and embedding-based approaches that integrate RAG-derived, context-specific biological knowledge to support more nuanced and context-aware functional interpretation beyond what can be achieved using static curated annotations alone. Such approaches could enable increasingly context-aware and mechanistically informed interpretation of complex molecular phenotypes across diverse biological and disease contexts.

We employed the Mann-Whitney U test, a robust nonparametric method for comparing similarity distributions, to assess whether gene or function embeddings showed significantly higher associations than expected by chance. To complement this, we used Cliff’s delta as a nonparametric effect-size measure, ensuring that statistical significance corresponded to biologically meaningful differences in embedding similarity. This combination avoids the assumption of normality, which is often violated in correlation-based similarity measurements. Although permutation-based or bootstrapped tests could, in principle, better account for the inherent dependencies among genes within functional sets, such approaches are computationally impractical for large-scale analyses involving thousands of gene sets, as performed in this study. Our framework thus balances statistical rigor, interpretability, and scalability, while future implementations could incorporate adaptive or approximate resampling schemes to account for complex gene-gene dependencies.

A notable limitation of embedding-based approaches is the inability to capture directionality in biological functions. Semantic similarity alone cannot distinguish whether a gene activates or represses a given function, a challenge also faced by traditional gene set enrichment methods and LLM-based inference models. Addressing this limitation may require integrating embeddings with direction-aware information, such as gene expression magnitude, causal inference methods, or specialized representation techniques.

In summary, our work provides a comprehensive statistical assessment of gene and function embeddings derived from LLMs. The proposed framework enables not only systematic benchmarking but also efficient and scalable hypothesis testing, even in contexts where curated gene sets are unavailable. As LLMs continue to evolve and drive increasingly ambitious biomedical applications, embedding-based methods like ours are well-positioned to support statistical rigor, interpretability, and translational relevance.

## Methods

### Implementation of LLM embedding models

We evaluated embeddings generated from seven state-of-the-art LLMs, including two proprietary models (text-embedding-3-large [OpenAI-TE3] from OpenAI and gemini-embedding-001 [Gemini-E1]^15^ from Google AI) and five open-source models: pubmedbert-base-embeddings (PubMedBERT^18^) from NeuML, e5-mistral-7b-instruct (Mistral)^17^ from Microsoft, MedCPT-Article Encoder (MedCPT; https://huggingface.co/ncbi/MedCPT-Article-Encoder)^19^ from NCBI, OpenBioLLM-70B (OpenBioLLM; https://huggingface.co/aaditya/Llama3-OpenBioLLM-70B) from Saama, and Llama-3-70B (Llama-3)^16^ from Meta. Detailed information and sources of these embedding methods are provided in Supplementary Table 1. The two proprietary models were accessed using their respective application programming interfaces (APIs). Both models are general-purpose and trained on data from diverse domains. Among the five open-source models, Mistral and Llama-3 are general-purpose, while PubMedBERT, MedCPT, and OpenBioLLM are primarily trained or finetuned on biomedical data. PubMedBERT, Mistral, and MedCPT were sourced from Hugging Face and deployed locally, whereas OpenBioLLM and Llama-3 were downloaded and executed using Ollama. The embeddings vary in their dimensions, ranging from 768 (PubMedBERT and MedCPT) to 8192 (OpenBioLLM and Llama-3). For comparison, we also included BioWordVec^20^, a non-LLM, word-based model trained on PubMed and MIMIC-III clinical notes by the NLM/NCBI BioNLP Research Group, which generates 200-dimensional embeddings.

### Generation of gene embeddings

This study focused on human protein-coding genes because they represent the most well-annotated portion of the genome, with reliable functional summaries, and are typically the primary focus of functional annotation analyses. We obtained functional summaries of individual genes from three widely used databases: (1) NCBI Gene^32^, which curates descriptions from multiple sources such as RefSeq under the “Summary” field, (2) Alliance of Genome Resources^33^, where descriptions are generated from structured gene data such as ontology terms and ortholog sets under the “Automated Description” field, and (3) UniProt^34^, which focuses on protein-level functions under the “Function” field. The NCBI database was accessed using its API, Entrez Programming Utilities (E-utilities), while descriptions from Alliance of Genome Resources and UniProt were directly downloaded from their respective databases. The number of genes retrieved from each database was 18,753 from NCBI, 18,600 from Alliance of Genome Resources, and 16,343 from UniProt, with 16,329 genes shared across all three databases. We preprocessed the gene descriptions to remove irrelevant information, such as source attribution found in NCBI descriptions (e.g., “[provided by RefSeq, Jul 2008]”). After preprocessing, each gene name was combined with its corresponding functional summary and input into the embedding models to generate gene embeddings.

We also generated de novo gene descriptions using an RAG approach. For each gene, we first retrieved the 100 most relevant sentences from PubMed and PubMed Central via the LitSense^35^ API using biological context-specific queries: “function of ‘gene symbol’”, “‘gene symbol’ pathway association”, “‘gene symbol’ transcriptional response”, “‘gene symbol’ disease association”. The numbers of genes under each biological context were 18,723, 18,723, 18,288, and 18,548, respectively. To restrict the temporal scope of knowledge and ensure controlled evaluation, we filtered the retrieved sentences to retain only those from publications published in or before October 2023, consistent with the versions of curated gene databases used in this study. For each gene, up to 40 retrieved sentences (determined based on our recent evaluation^9^) were summarized by GPT-4o from OpenAI (model gpt-4o-2024-08-06, knowledge cutoff Oct 1, 2023) accessed via the OpenAI API, using the following prompt:

“Based on the scientific materials provided below (sentences retrieved from PubMed), write a single coherent paragraph summarizing the function of the gene ‘gene symbol’. Ensure that the summary is accurate, concise, and integrates the key findings without redundancy.” For different biological contexts used in RAG, we modified the phrase “function of the gene ‘gene symbol’” accordingly, for example, to “pathway association of the gene ‘gene symbol’” to generate context-specific descriptions.

### Construction and visualization of gene embedding clusters

To analyze the relationships among gene embeddings, we applied *k*-means clustering to group all 18,723 genes into 107 clusters based on cosine similarity in the gene embeddings generated by OpenAI-TE3 using RAG-function gene summaries. The optimal number of clusters was determined using an enhanced elbow method that identified the inflection point by measuring the perpendicular distance of the sum of squared errors curve across cluster configurations ranging from 15 to 300 clusters. The results were visualized using a *t*-SNE plot. Cluster compactness was quantified by the average cosine distance of each gene to its cluster center in the 3072-dimensional space. A smaller value indicates a more compact cluster. For subsequent interpretation analysis, we focused on the representative gene with the highest mean cosine similarity to all other genes in the cluster.

### Annotation and interpretation of gene embedding clusters via ablation analysis

To annotate each cluster, we extracted the top 10 representative biological terms (named entities) from the descriptions of genes in each cluster. We used the *en_core_sci_lg* model from the Python scispaCy package^36^ to recognize biomedical named entities within the descriptions, followed by lemmatization. Within each cluster, entities were ranked based on their occurrence frequency, and the top ten were selected as representative keywords. For each cluster, we generated a concise, descriptive annotation of the 10 keywords using an automated pipeline with GPT-4o (model gpt-4o-2024-08-06) using the following prompt:

“These ten terms are derived from the functional descriptions of a set of genes, listed in decreasing order of importance; please summarize the biological functions they represent in a single ‘gene function term’ from a biomedical perspective in a concise way (No more than 5 words, just give me the ‘gene function term’), with an emphasis on highlighting the relationships between these keywords.”

To further interpret the semantic structure of each cluster, we performed an ablation analysis to assess the contribution of individual biological terms to local embedding organization. Because named entity recognition is context dependent, we first merged the descriptions of all genes within each cluster to ensure coherent entity extraction. For each identified entity, we generated ablated versions of the gene descriptions by removing that entity from all member genes and then regenerated the corresponding gene embeddings using the same OpenAI-TE3 model. We computed all pairwise cosine similarities among genes within a cluster to evaluate how the removal of each term affected intra-cluster cohesion. The change in average cosine similarity served as a quantitative measure of the influence of each term. Changes in cosine similarity were expressed as percentage differences relative to the original median intra-cluster cosine similarity.

### Functional annotation databases, corresponding gene sets, and generation of function embeddings

To comprehensively evaluate the functional relevance of embeddings, we analyzed diverse functional annotations based on different definitions and taxonomies (Supplementary Table 3). Specifically, we obtained descriptions and associated gene sets for four types of functional annotations from MSigDB^3^ (version, 2023.2.Hs) and GWAS Catalog^14^ (version, 1.0.2): GO-BP within C5 ontology gene sets; CP (curated signaling and metabolic pathways) and CGP (gene sets reflecting expression changes in response to perturbations) within C2 curated gene sets; and diseases mapped to Experimental Factor Ontology terms descending from EFO:0000408 (“disease”) in the GWAS Catalog. To avoid overly specific or generalized functional terms, we included only gene sets containing 15 to 500 genes, following the commonly used thresholds in gene set enrichment analysis^2^. As a result, 4146 GO-BP, 2438 CP, 2691 CGP, and 301 GWAS functional annotations were analyzed.

For MSigDB terms, we generated function embeddings by combining the name of each functional annotation (“Standard name” in MSigDB), replacing underscores with spaces and removing prefixes such as “GO_BP”, with its corresponding description (“Brief description”), after removing brackets like “[GeneID = …].” The combined text was then converted into function embeddings using the LLMs. For GWAS terms, which typically describe disease phenotypes or symptoms rather than explicit biological processes, we generated functional descriptions using GPT-4o (model: gpt-4o-2024-08-06) with the following prompt:

“Write a paragraph to describe what is known about the underlying molecular functions and mechanisms of the disease ‘disease name’.”

### Analysis of gene-gene functional similarities and statistical test

We systematically assessed whether the similarity between gene embeddings reflects their functional similarities. Genes within the same gene set corresponding to a functional annotation were assumed to share a similar function. For a given gene set, we evaluated whether gene-gene cosine similarities are significantly higher among gene pairs within the set compared to inter-gene set pairs (i.e., pairs where one gene belongs to the set and the other does not; Fig. 3a). A one-tailed Mann-Whitney U test *p* < 0.05 with Cliff’s delta *δ* ≥ 0.147 was used to define statistical significance. Multiple testing correction was not applied, as our objective was to summarize the proportion of functions captured by gene embeddings and compare overall trends across embedding models, rather than to make formal statistical inferences for individual functions.

We also analyzed the effect of gene set size on performance. Based on the distribution of GO-BP sizes, we categorized all gene sets into three groups: small (15–30 genes), medium (31–70 genes), and large (71–500 genes), using the nearest 5-gene increment cutoffs that yield similarly sized subgroups (see Supplementary Fig. 10). We applied the same criteria in the subsequent gene-function analyses.

### Sensitivity and specificity analysis for gene-function relationships

For gene-function analysis, we tested whether a function embedding (derived from the description of a functional annotation) is highly similar to the gene embeddings of its corresponding gene set. To assess the robustness of gene-function associations, we analyzed both sensitivity and specificity. Sensitivity was evaluated by determining whether the similarity between a function embedding and the gene embeddings of its designated gene set was significantly higher than the similarity between the function embedding and genes outside the designated gene set (Fig. 4a). Multiple testing correction was not applied, as the goal was to assess overall trends rather than test specific hypotheses.

Specificity was evaluated to account for potential dependencies among functional annotations and their gene sets. We tested whether gene embeddings from a given gene set were more strongly correlated with their corresponding function embedding than with embeddings from other functions (Fig. 5a). A gene-function relationship was considered specific if its median similarity difference (*Δ*) ranked within the top 5% among all functional annotations (e.g., all GO-BP terms), consistent with the threshold established in a recent study^5^. The median similarity difference was defined as the difference between (1) the median cosine similarity of genes to their corresponding function embedding and (2) the median cosine similarity of all other genes to the same function embedding, conceptually aligned with the Mann-Whitney U test used in the gene-function sensitivity analysis (Fig. 4a).

We used the results of GSAI^5^ as a baseline for gene-function specificity analysis, as it assesses semantic specificity among GO Biological Process terms. To ensure a consistent comparison, we used the same 1000 GO-BP terms and corresponding gene sets analyzed in the GSAI study. For each gene set, GSAI employed GPT-4 to predict a function name based on the input genes. SapBERT^37^ was then used to embed both the predicted function name and each of the 11,943 GO-BP term names, including the 1000 used for testing. Cosine similarity was calculated between the predicted name and each GO-BP term name. A GO-BP term was considered correctly identified if the corresponding true term ranked within the top 5% in cosine similarity among all 11,943 terms (https://github.com/idekerlab/llm_evaluation_for_gene_set_interpretation/blob/main/data/go_terms.csv). We obtained GSAI results from its GitHub repository (https://github.com/idekerlab/llm_evaluation_for_gene_set_interpretation/blob/main/data/GO_term_analysis/simrank_LLM_processed_selected_1000_go_terms.tsv). To compare against this benchmark, we applied the same 1000 gene sets (https://github.com/idekerlab/llm_evaluation_for_gene_set_interpretation/blob/main/data/GO_term_analysis/1000_selected_go_terms.csv) to our embedding-based framework. We assessed specificity by ranking differences in median cosine similarities derived from our gene-function analysis framework. Performance was also evaluated across a range of specificity thresholds to ensure comprehensive assessment.

### De novo hypothesis generation and testing for gene functions

We generated functional hypotheses for a list of genes of interest using the GSAI model^5^. We implemented a slightly modified version of GSAI, incorporating the updated prompt and the newer GPT-4o model used by the web tool, to predict up to five functions associated with a given gene list (see Supplementary Method 1 for implementation details). For each predicted function, the LLM generates a functional summary and a confidence score. We then constructed a function embedding by combining the function term with its corresponding summary and tested its similarity to the input gene list.

### Contamination analysis of LLM-generated functional hypotheses

To evaluate GSAI-generated results, we applied our framework to a contamination test across 4146 GO-BP gene sets (Fig. 6a). For each gene set, we systematically introduced contamination by replacing genes with randomly sampled genes in 5% increments, ranging from 0% (true gene set) up to 100% contamination (fully contaminated set with random genes). These contaminated gene sets were then input into GSAI, which inferred up to five biological functions per set. As mentioned above, the embedding of each predicted function was tested against the corresponding contaminated gene set. Finally, we compared the *p* and *δ* values with the GSAI-generated confidence scores across different contamination levels.

### Evaluation using NeST protein system gene lists

NeST comprises hierarchically organized protein assemblies identified through multiscale community detection applied to an integrated protein interaction network. We analyzed 50 representative NeST systems previously used in LLM benchmarking studies, downloaded from https://github.com/ncbi-nlp/GeneAgent/tree/main/Datasets/NeST. To construct a semantic reference ranking, we computed cosine similarity between embeddings generated by the MedCPT Query Encoder (https://huggingface.co/ncbi/MedCPT-Query-Encoder) for GO-BP term names and the corresponding NeST annotation (Fig. 7a). The choice of the MedCPT Query Encoder followed a recent study^6^ and is distinct from the MedCPT Article Encoder evaluated as one of the embedding models elsewhere in this study. GO-BP terms were then ranked according to this semantic similarity for each NeST system. For evaluation of LLM-inferred functions, GPT-4o was prompted to infer up to twenty functional terms with descriptions for each NeST system using a modified version of the prompt shown in Supplementary Fig. 8. For embedding-based analysis, each inferred functional term was concatenated with its generated description and converted into an embedding representation prior to statistical testing using the gene-function sensitivity framework described above. For each LLM-inferred functional term, a corresponding gene set was generated by GPT-4o using a dedicated prompt (Supplementary Fig. 11), following the strategy described in a recent publication^7^. These inferred gene sets were subsequently used to perform ORA against the NeST gene list for the corresponding system.

### Statistics and reproducibility

Statistical analyses were performed using the methods described in the corresponding Methods sections and are summarized in the relevant figure legends. The embedding-based framework defined statistical significance using a one-tailed Mann-Whitney U test (*p* < 0.05) together with a non-negligible effect size (Cliff’s delta *δ* ≥ 0.147). Comparative analyses were performed using one-tailed Wilcoxon signed-rank tests for paired comparisons, and Mann-Whitney U tests for independent samples, with the tail setting specified for each analysis. Ranking concordance was assessed using Spearman rank correlation. No statistical method was used to predetermine sample size. Sample sizes were determined by the number of genes, gene sets, functional annotations, or LLM-inferred hypotheses available for each analysis, as described in the corresponding Methods sections. No data were excluded after applying the predefined inclusion criteria. Analyses requiring a minimum number of observations (for example, at least three samples for calculation of Spearman rank correlations) were performed only when the predefined inclusion criteria were met. To ensure reproducibility, source data underlying the relevant figures and tables are provided as a Source Data file, and the source code and processed datasets generated in this study are publicly available through Zenodo. Additional information on data and code availability is provided in the Data Availability and Code Availability sections.

### Reporting summary

Further information on research design is available in the Nature Portfolio Reporting Summary linked to this article.

## Supplementary information


Supplementary Information
Description of Additional Supplementary Files
Supplementary Data 1
Supplementary Data 2
Supplementary Data 3
Supplementary Data 4
Reporting Summary
Transparent Peer Review file


## Source data


Source data


## Data Availability

All data used in this study are publicly available. The processed datasets generated and analyzed in this study are publicly available through Zenodo (https://doi.org/10.5281/zenodo.21044582)^38^. Gene descriptions were obtained from NCBI through the Entrez API, the Alliance of Genome Resources, and UniProt. Gene sets and functional annotation resources were obtained from MSigDB, including the Gene Ontology Biological Process (GO-BP), Canonical Pathway (CP), and Chemical and Genetic Perturbation (CGP) collections (versions, v2023.2.Hs and v2025.1.Hs). Additional public datasets used for benchmarking and evaluation were obtained from the GWAS Catalog; the full and 1000 selected GO-BP terms from Hu et al.^5^. (GitHub links are provided in the Methods section); the 50 NeST gene lists from Wang et al.^6^. (GitHub link is provided in the Methods section); and LINCS data from Supplementary Table 3 of Hu et al.^5^. Source data are provided in this paper.

## Code availability

The R package implementing the present analysis is available on GitHub (https://github.com/chiu-lab/DEGEmbedR; https://doi.org/10.5281/zenodo.21382882)^26^. All code used in this study, including the full implementation of the computational framework, data preprocessing, and evaluation, is available at the Zenodo link cited in the Data availability section.

## References

[1] Feuermann, M. et al. A compendium of human gene functions derived from evolutionary modelling. *Nature* **640**, 146–154 (2025). 40011791 10.1038/s41586-025-08592-0 PMC11964926

[2] Subramanian, A. et al. Gene set enrichment analysis: a knowledge-based approach for interpreting genome-wide expression profiles. *Proc. Natl. Acad. Sci. USA* **102**, 15545–15550 (2005). 16199517 10.1073/pnas.0506580102 PMC1239896

[3] Liberzon, A. et al. Molecular Signatures Database (MSigDB) 3.0. *Bioinformatics* **27**, 1739–1740 (2011). 10.1093/bioinformatics/btr260 PMC3106198 21546393

[4] Mooney, M. A., Nigg, J. T., McWeeney, S. K. & Wilmot, B. Functional and genomic context in pathway analysis of GWAS data. *Trends Genet.* **30**, 390–400 (2014). 25154796 10.1016/j.tig.2014.07.004 PMC4266582

[5] Hu, M. et al. Evaluation of large language models for discovery of gene set function. *Nat. Methods* **22**, 82–91 (2024). 39609565 10.1038/s41592-024-02525-x PMC11725441

[6] Wang, Z. et al. GeneAgent: self-verification language agent for gene-set analysis using domain databases. *Nat. Methods* **22**, 1677–1685 (2025). 40721871 10.1038/s41592-025-02748-6 PMC12328209

[7] Zhu, J. et al. Enhancing gene set overrepresentation analysis with large language models. *Bioinform. Adv.* **5**, vbaf054 (2025). 40401046 10.1093/bioadv/vbaf054 PMC12093311

[8] Lin, X. et al. GeneRAG: enhancing large language models with gene-related task by retrieval-augmented generation. Preprint at 10.1101/2024.06.24.600176 (2024).

[9] Lai, Y. J. et al. Inferring drug-gene relationships in cancer using literature-augmented large language models. *Cancer Res. Commun.* **5**, 706–718 (2025). 40293950 10.1158/2767-9764.CRC-25-0030 PMC12036822

[10] Chen, Y. & Zou, J. Simple and effective embedding model for single-cell biology built from ChatGPT. *Nat. Biomed. Eng.* **9**, 483–493 (2024). 10.1038/s41551-024-01284-6 39643729

[11] Boyle, I. A. et al. Natural language processing of gene descriptions for overrepresentation analysis with GeneTEA. *Genome Biol.* **26**, 376 (2025). 41168869 10.1186/s13059-025-03844-8 PMC12577433

[12] Tao, C. et al. LLMs are also effective embedding models: an in-depth overview. Preprint at 10.48550/arXiv.2412.12591 (2024).

[13] Freestone, M. & Santu, S. K. K. Word embeddings revisited: Do LLMs offer something new? Preprint at 10.48550/arXiv.2402.11094 (2024).

[14] Sollis, E. et al. The NHGRI-EBI GWAS Catalog: knowledgebase and deposition resource. *Nucleic Acids Res.* **51**, D977–D985 (2023). 36350656 10.1093/nar/gkac1010 PMC9825413

[15] Lee, J. et al. Gecko: Versatile text embeddings distilled from large language models. Preprint at 10.48550/arXiv.2403.20327 (2024).

[16] Dubey, A. et al. The Llama 3 herd of models. Preprint at 10.48550/arXiv.2407.21783 (2024).

[17] Jiang, A. Q. et al. Mistral 7B. Preprint at 10.48550/arXiv.2310.06825 (2023).

[18] Gu, Y. et al. Domain-specific language model pretraining for biomedical natural language processing. *ACM Trans. Comput. Healthc.* **3**, 1–23 (2021).

[19] Jin, Q. et al. MedCPT: Contrastive Pre-trained Transformers with large-scale PubMed search logs for zero-shot biomedical information retrieval. *Bioinformatics* **39**, btad651 (2023). 37930897 10.1093/bioinformatics/btad651 PMC10627406

[20] Zhang, Y., Chen, Q., Yang, Z., Lin, H. & Lu, Z. BioWordVec, improving biomedical word embeddings with subword information and MeSH. *Sci. Data* **6**, 52 (2019). 31076572 10.1038/s41597-019-0055-0 PMC6510737

[21] O’Duibhir, E. et al. Cell cycle population effects in perturbation studies. *Mol. Syst. Biol.* **10**, 732 (2014). 24952590 10.15252/msb.20145172 PMC4265054

[22] Szalai, B. & Saez-Rodriguez, J. Why do pathway methods work better than they should? *FEBS Lett.* **594**, 4189–4200 (2020). 33270910 10.1002/1873-3468.14011

[23] Chambers, B. & Shah, I. Evaluating adaptive stress response gene signatures using transcriptomics. *Comput. Toxicol.* **20**, 1–9 (2021). 37829472 10.1016/j.comtox.2021.100179 PMC10569130

[24] Steyvers, M. et al. What large language models know and what people think they know. *Nat. Mach. Intell.* **7**, 221–231 (2025).

[25] Zheng, F. et al. Interpretation of cancer mutations using a multiscale map of protein systems. *Science* **374**, eabf3067 (2021). 34591613 10.1126/science.abf3067 PMC9126298

[26] Tan, Y. et al. An embedding-based framework enables statistical testing of gene-set function hypotheses inferred by large language models. chiu-lab/DEGEmbedR: raw-data. *Zenodo*10.5281/zenodo.21382882 (2026). PMC13526818 42669697

[27] Subramanian, A. et al. A next generation Connectivity Map: L1000 platform and the first 1,000,000 profiles. *Cell* **171**, 1437–1452 (2017). 10.1016/j.cell.2017.10.049 PMC5990023 29195078

[28] Stagg, J. & Smyth, M. J. Extracellular adenosine triphosphate and adenosine in cancer. *Oncogene* **29**, 5346–5358 (2010). 20661219 10.1038/onc.2010.292

[29] Antonioli, L., Blandizzi, C., Pacher, P. & Hasko, G. Immunity, inflammation and cancer: a leading role for adenosine. *Nat. Rev. Cancer* **13**, 842–857 (2013). 24226193 10.1038/nrc3613

[30] Pasquale, E. B. Eph receptors and ephrins in cancer: bidirectional signalling and beyond. *Nat. Rev. Cancer* **10**, 165–180 (2010). 20179713 10.1038/nrc2806 PMC2921274

[31] Grimm, D. et al. The role of SOX family members in solid tumours and metastasis. *Semin. Cancer Biol.* **67**, 122–153 (2020). 30914279 10.1016/j.semcancer.2019.03.004

[32] Sayers, E. W. et al. Database resources of the National Center for Biotechnology Information. *Nucleic Acids Res.* **52**, D33–D43 (2024). 37994677 10.1093/nar/gkad1044 PMC10767890

[33] Alliance of Genome Resources Consortium. Alliance of Genome Resources Portal: unified model organism research platform. *Nucleic Acids Res.* **48**, D650–D658 (2020). 10.1093/nar/gkz813 PMC6943066 31552413

[34] UniProt Consortium. UniProt: the universal protein knowledgebase in 2025. *Nucleic Acids Res.* **53**, D609–D617 (2025). 10.1093/nar/gkae1010 PMC11701636 39552041

[35] Allot, A. et al. LitSense: making sense of biomedical literature at sentence level. *Nucleic Acids Res.* **47**, W594–W599 (2019). 31020319 10.1093/nar/gkz289 PMC6602490

[36] Neumann, M., King, D., Beltagy, I. & Ammar, W. ScispaCy: Fast and robust models for biomedical natural language processing. Preprint at 10.48550/arXiv.1902.07669 (2019).

[37] Liu, F., Shareghi, E., Meng, Z., Basaldella, M. & Collier, N. Self-alignment pretraining for biomedical entity representations. Preprint at 10.48550/arXiv.2010.11784 (2021).

[38] Tan, Y. et al. An embedding-based framework enables statistical testing of gene-set function hypotheses inferred by large language models. Code and data for: “An embedding-based framework enables statistical testing of gene-set function hypotheses inferred by large language models.” *Zenodo*10.5281/zenodo.21044582 (2026). PMC13526818 42669697

