# Supplementary material for: An embedding-based framework enables statistical testing of gene-set function hypotheses inferred by large language models
Source: Nat Commun. 2026 Jul 30;17:9248. doi: 10.1038/s41467-026-75972-z (PMC13526818; doi:10.1038/s41467-026-75972-z)
Supplement: Supplementary file 1 [file 41467_2026_75972_MOESM1_ESM.pdf]

Supplementary Information

An embedding-based framework enables statistical testing of gene-set function hypotheses inferred by large language models

Yanhao Tan, Li-Ju Wang, Tianyuzhou Liang, Ying-Ju Lai, Chien-Hung Shih, Yibing Guo, Tyler M. Yasaka, George C. Tseng, Yu-Chiao Chiu

- Supplementary Method 1
- Supplementary Tables 1-3
- Supplementary Figures 1-11

Supplementary Methods

Supplementary Method 1. Implementation of GSAI for hypothesis generation

We implemented GSAI to generate functional hypotheses for a gene list. GSAI is a prompt-based approach that uses an inference LLM to predict gene functions and assign a confidence score ranging from 0 to 1 to each inferred function. At the time of our study, GSAI was available in two versions:

(1) The original prompt using GPT-4, as described and evaluated in the publication (accessible at https://idekerlab.ucsd.edu/gsai-hu-et-al-2024-llm-gene-sets/ and the publication[1]).
(2) An updated prompt using the newer GPT-4o model (API version, gpt-4o-2024-08-06), implemented in the web tool (the Biological Process Analysis prompt; https://idekerlab.ucsd.edu/gsai/), which the tool recommends for users.

In this study, we adopted the overall structure of the updated prompt, as it is the recommended version. In addition to using the newer GPT-4o model, the updated version replaces the numeric confidence scores used in the publication with qualitative confidence levels (high, medium, or low). To enable quantitative analyses and comparisons, we retained the numeric confidence score assignment instructions from the original prompt. Additionally, to ensure consistent output formatting and unbiased summarization of results, we slightly modified the prompt to constrain the output to a maximum of five functions, each accompanied by a description and a numeric confidence score. The generated function names and descriptions were then converted into function embeddings for subsequent analyses.

Supplementary Fig. 8 presents the final prompt used in our study. Content originating from the updated and original GSAI prompts is shown in black and red, respectively, while the modifications introduced in our study are shown in blue. The final prompt was implemented using GPT-4o (API version: gpt-4o-2024-08-06), consistent with the version used in the latest GSAI web tool.

Supplementary Tables

Supplementary Table 1. Embedding models used in this study.

Model	Abbreviation	Training data	Embedding dimension	Type	Developer	Access	Release date
**text-embedding-3-large**	OpenAI-TE3	Internet text using neighboring text as positive pairs	3,072	Proprietary	OpenAI	API	Jan 25, 2024
**gemini-embedding-001**	Gemini-E1	--	3,072	Proprietary	Google	API	July 14, 2025
**e5-mistral-7b-instruct**	Mistral	Synthetic multilingual datasets generated by LLMs across 93 languages	4,096	Open-source	Microsoft	Hugging Face (intfloat/ e5-mistral-7b-instruct)	Dec 20, 2023[a]
**pubmedbert-base-embeddings**	PubMedBERT	A random sample of PubMed title-abstract and similar title pairs	768	Open-source	NeuML	Hugging Face (NeuML/ pubmedbert-base-embeddings)	Oct 18, 2023[a]
**MedCPT-Article-Encoder**	MedCPT	255M query-article pairs from PubMed search logs	768	Open-source	NCBI	Hugging Face (ncbi/ MedCPT-Article-Encoder)	Oct 25, 2023[a]
**OpenBioLLM-70B**	OpenBioLLM	Vast corpus of high-quality biomedical data	8,192	Open-source	Saama	Hugging Face/ Ollama (aaditya/ Llama3-OpenBioLLM-70B)[c]	Apr 23, 2024[a]
**Llama-3-70B**	Llama-3	Mix of publicly available online data	8,192	Open-source	Meta	Ollama (llama3:70b)	Apr 18, 2024
**BioWordVec (non-LLM)**	BioWordVec	28M sentences from PubMed and 2M sentences from MIMIC III Clinical notes	200	Open-source	NLM/NCBI BioNLP Research Group	GitHub (ncbi-nlp/ BioSentVec)	Oct 31, 2018[b]

[a]Release date on Hugging Face.
[b]Release date on GitHub.
[c]Downloaded from https://huggingface.co/mradermacher/Llama3-OpenBioLLM-70B-GGUF

Supplementary Table 2. Gene description data resources.

Source	Number of genes	Description length (words)[a]	Gene type	Organism	Access date
**NCBI Gene (NCBI)**	18,753	62 (3-528)	Protein coding	Homo sapiens	Feb. 2024
**Alliance of Genome Resources (Alliance)**	18,600	36 (3-167)	Protein coding	Homo sapiens	May 2024
**Universal Protein Resource (UniProt)**	16,343	72 (1-1,507)	Protein coding	Homo sapiens	May 2024
**Retrieval-augmented generation (RAG)-function**	18,723	170 (50-283)	Protein coding	Homo sapiens	Up to 2023
**RAG-pathway**	18,723	179 (81-293)	Protein coding	Homo sapiens	Up to 2023
**RAG-perturbation**	18,288	181 (76-281)	Protein coding	Homo sapiens	Up to 2023
**RAG-disease**	18,584	178 (76-280)	Protein coding	Homo sapiens	Up to 2023

[a]Average (range).

Supplementary Table 3. Functional annotation databases.

Database	Number of functions	Usage	Gene count[a]	Description length (words)[a]	Source	Version
**Gene Ontology Biological Process (GO-BP)**	4,146	Focused annotations as systematic evaluation	80 (15-500)	27 (5-153)	MSigDB (C5.GO.BP)	2023.2.Hs
**Canonical Pathway (CP)**	2,438	Focused annotations as systematic evaluation	54 (15-477)	6 (1-33)	MSigDB (C2.CP)	2023.2.Hs
**Chemical and Genetic Perturbations (CGP)**	2,691	Diffuse annotations for benchmarking	98 (15-500)	18 (2-70)	MSigDB (C2.CGP)	2023.2.Hs
**Genome-Wide Association Study (GWAS)**	301	Diffuse annotations for benchmarking	59 (15-452)	178 (134-318)	GWAS Catalog	v1.0.2
**Gene Ontology Biological Process (sample from full database)**	1,000	Specificity evaluation and comparison with GSAI	20 (3-100)	26 (5-112)	GitHub (idekerlab/ llm_evaluation_ for_gene_set_in terpretation)	--

[a]Average (range).

Supplementary Figures

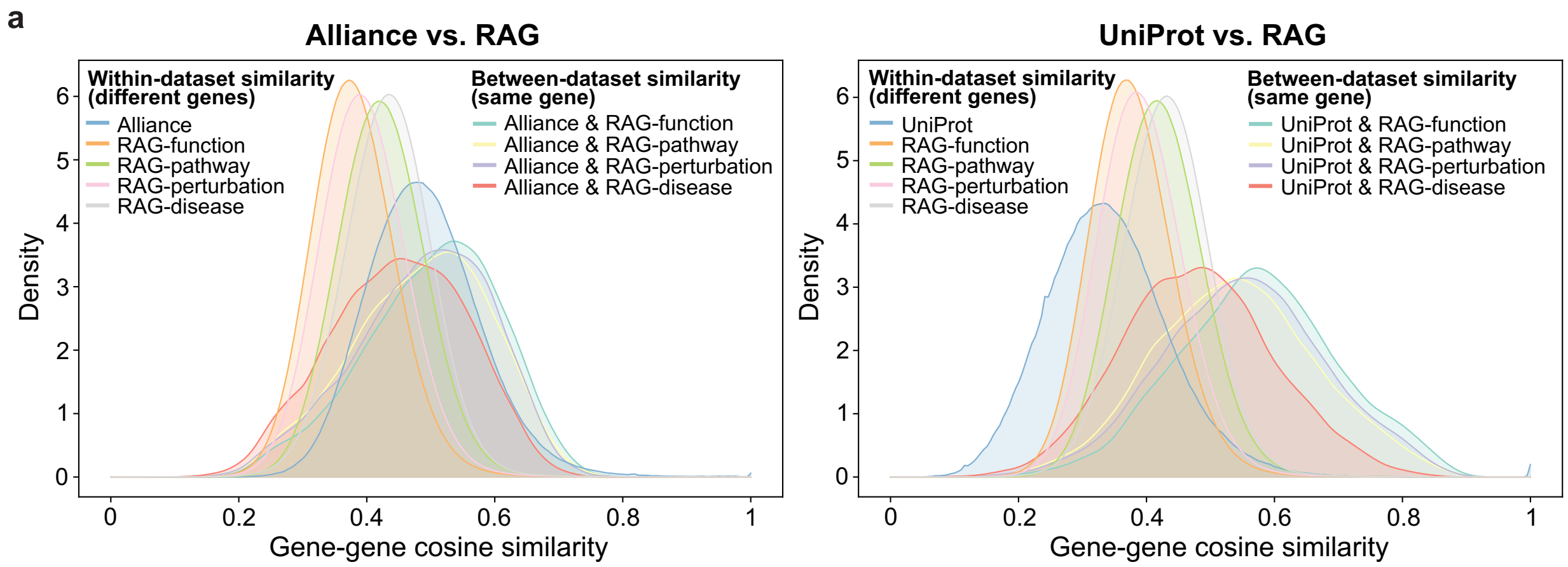

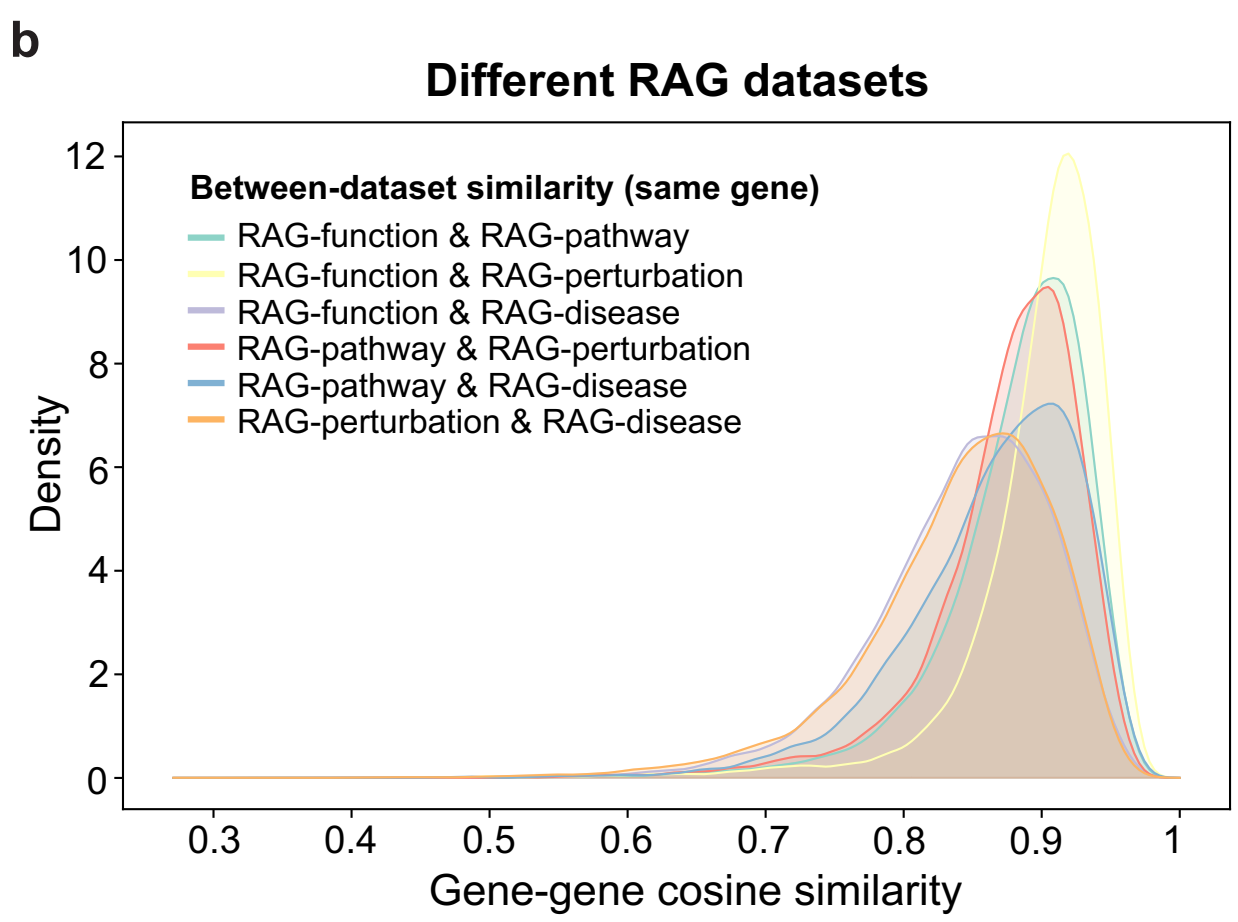

c **Between-dataset matched-gene similarity (median)**

	NCBI	Alliance	UniProt	RAG-function	RAG-pathway	RAG-perturbation	RAG-disease
NCBI	--	0.635	0.628	0.612	0.576	0.585	0.547
Alliance	0.635	--	0.627	0.512	0.494	0.498	0.454
UniProt	0.628	0.627	--	0.573	0.537	0.552	0.479
RAG-function	0.612	0.512	0.573	--	0.893	0.910	0.851
RAG-pathway	0.576	0.494	0.537	0.893	--	0.886	0.877
RAG-perturbation	0.585	0.498	0.552	0.910	0.886	--	0.852
RAG-disease	0.547	0.454	0.479	0.851	0.877	0.852	--
**Average**	**0.597**	**0.537**	**0.566**	**0.725**	**0.711**	**0.714**	**0.677**

Supplementary Fig. 1. Gene embedding similarities derived from curated databases and retrieval-augmented generation (RAG). (a) Distributions of cosine similarities for overlapping gene embeddings within and between individual data sources, including Alliance of Genome Resources (Alliance), UniProt, and RAG-derived descriptions (RAG-function, RAG-pathway, RAG-perturbation, and RAG-disease). Left panels show within-dataset similarity between different genes, and right panels show between-dataset similarity of matched genes. Density plots were generated using Gaussian kernel density estimation implemented in Python (SciPy, default parameters). **(b)** Between-dataset similarity for overlapping genes across pairs of RAG categories, illustrating cross-source consistency of gene embeddings derived from different retrieval contexts. **(c)** Median cosine similarity of matched gene embeddings between dataset pairs. Source data are provided as a Source Data file.

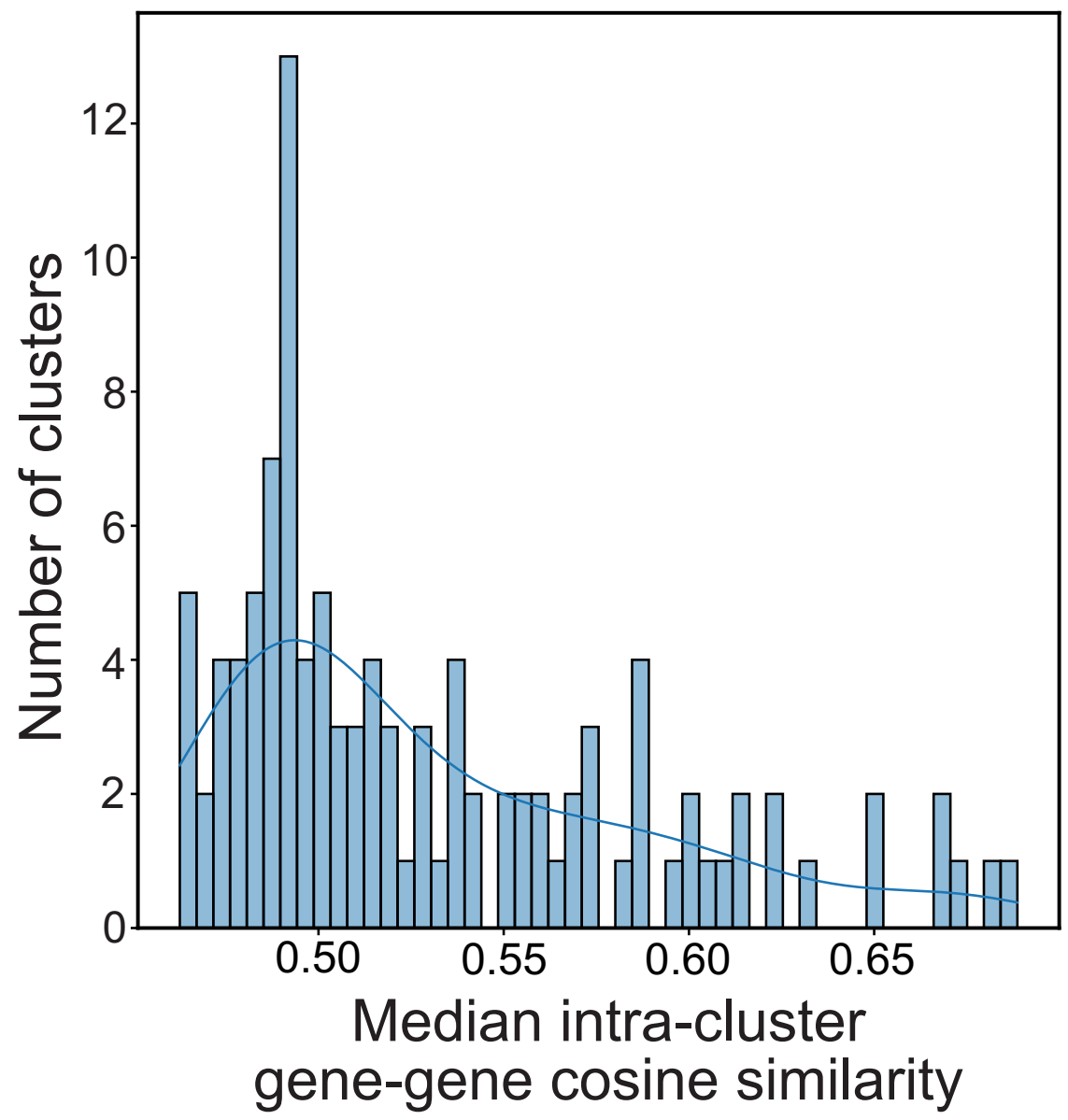

Supplementary Fig. 2. Distribution of intra-cluster gene-gene cosine similarity. Histogram showing the median intra-cluster cosine similarity for each of the 107 gene clusters derived from OpenAI-TE3 embeddings of RAG-based gene descriptions, corresponding to the clustering shown in Fig. 2b. Higher values indicate tighter semantic cohesion among genes within a cluster. Source data are provided as a Source Data file.

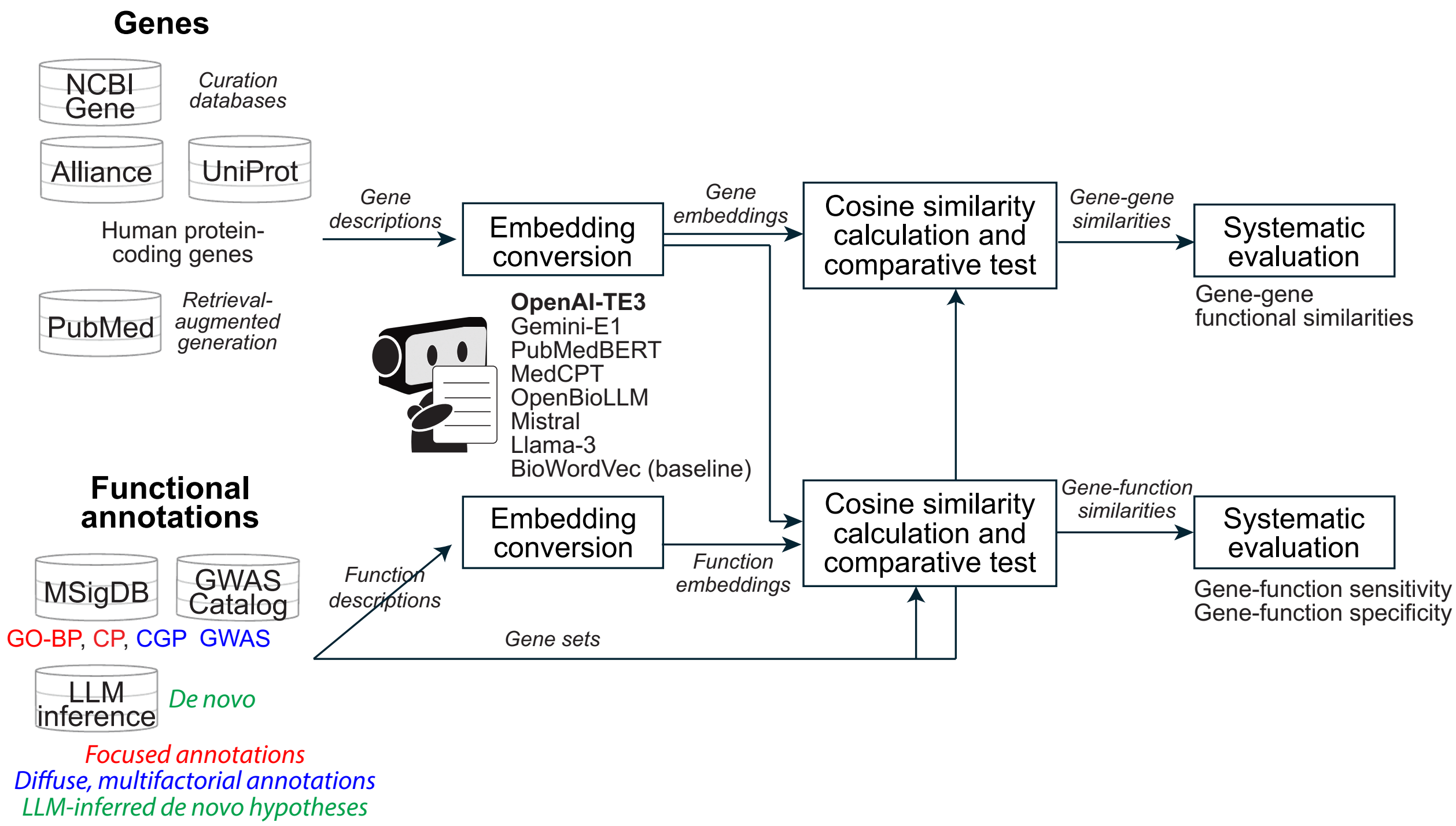

Supplementary Fig. 3. Flowchart of the computational framework for embedding-based functional evaluation. Gene descriptions were obtained from curated databases (NCBI Gene, Alliance, and UniProt) and retrieval-augmented generation (RAG) from biomedical literature. Functional annotation descriptions were collected from MSigDB (GO-BP, CP, CGP) and GWAS Catalog, and also included LLM-inferred *de novo* hypotheses. All textual descriptions were converted into embedding vectors using seven LLM-based models (OpenAI-TE3, Gemini-E1, PubMedBERT, MedCPT, Mistral, OpenBioLLM, and Llama-3) and one baseline model (BioWordVec). Cosine similarities were computed for gene-gene and gene-function pairs, followed by statistical comparison against corresponding gene sets. This framework enables systematic evaluation of gene-gene functional similarity and quantitative assessment of gene-function sensitivity and specificity across focused, diffuse, and *de novo* functional scenarios.

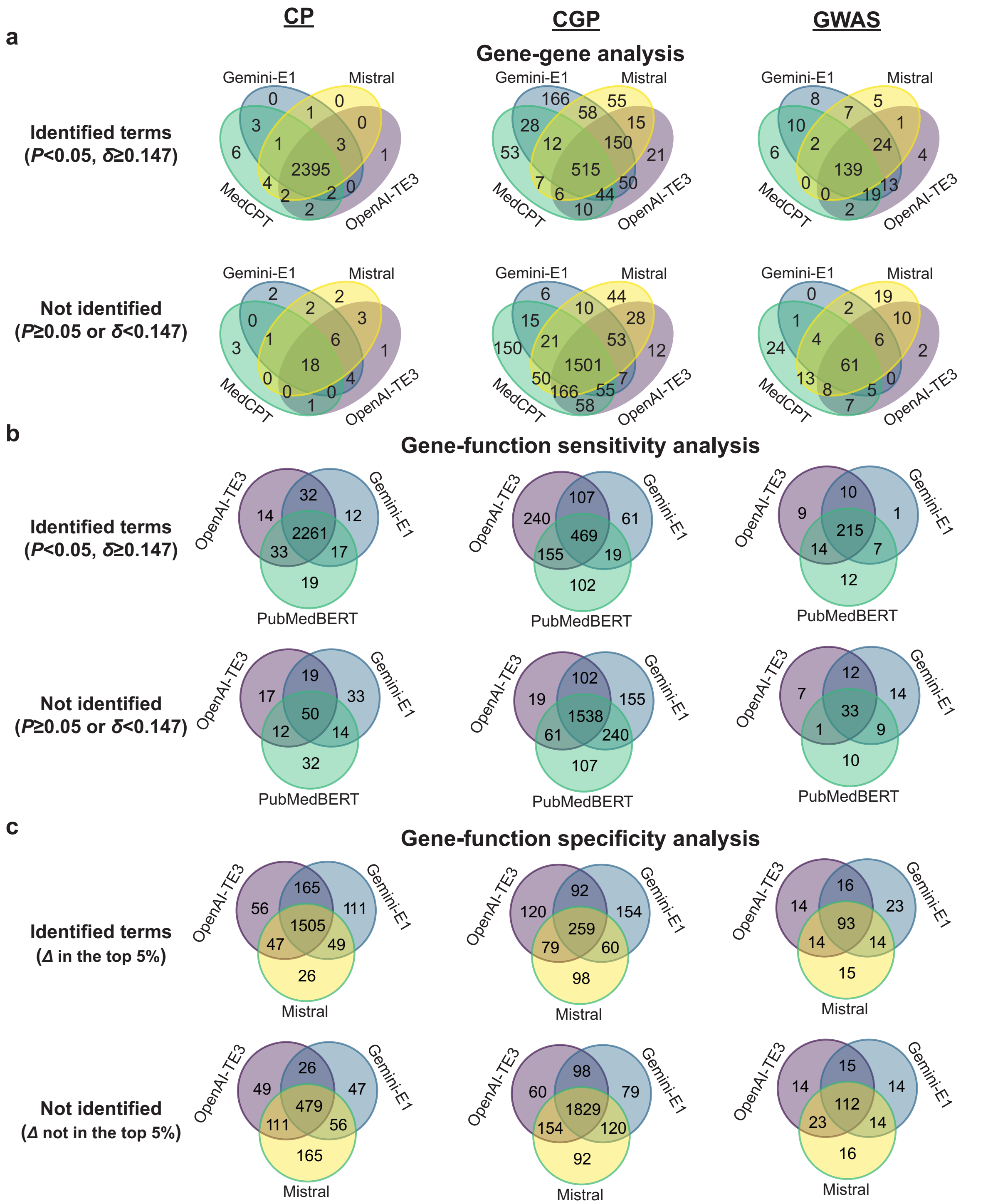

Supplementary Fig. 4. Venn diagrams of CP, CGP, and GWAS annotations identified and not identified by the best-performing embedding models. (a) Gene-gene analysis, corresponding to the GO-BP results in Fig. 3c of the main text. Statistical significance and effect size were assessed using one-tailed Mann-Whitney U tests and Cliff's delta δ, respectively. **(b)** Gene-function sensitivity analysis, corresponding to the GO-BP results in Fig. 4c. **(c)** Gene-function specificity analysis, corresponding to the GO-BP results in Fig. 5c. Source data are provided as a Source Data file.

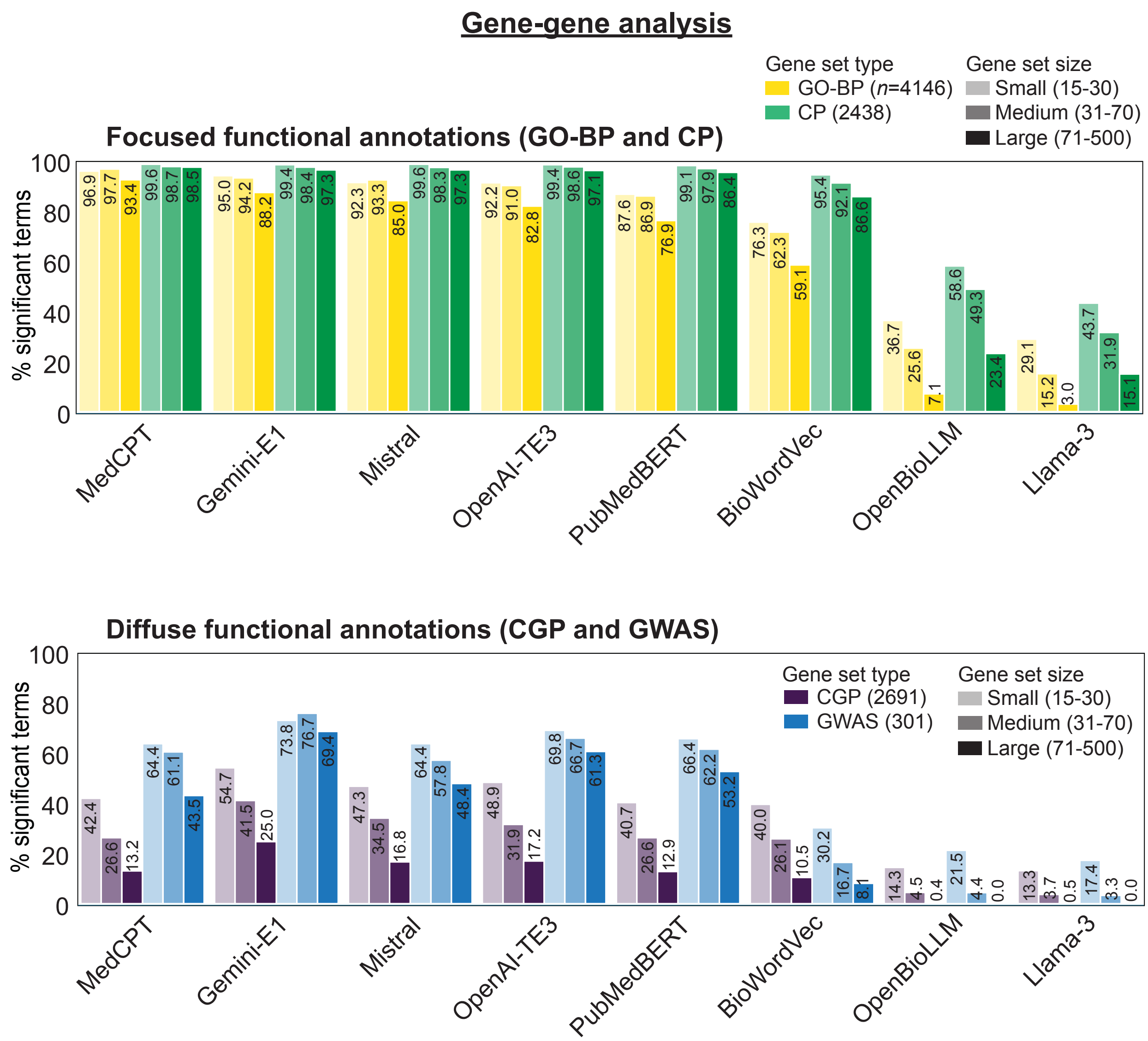

Supplementary Fig. 5. Effect of gene set size on gene-gene functional similarity performance. Gene-gene analysis shown as a size-stratified breakdown of Fig. 3b in the main text. Gene sets were categorized into small (15-30 genes), medium (31-70 genes), and large (71-500 genes) using nearest 5-gene increment cutoffs chosen to produce similarly sized subgroups in GO-BP (see Supplementary Fig. 10). Results are presented separately for focused functional annotations (GO-BP and CP) and diffuse annotations (CGP and GWAS). Significance was defined as a one-tailed Mann-Whitney U test ($p < 0.05$) and $\delta \geq 0.147$. Source data are provided as a Source Data file.

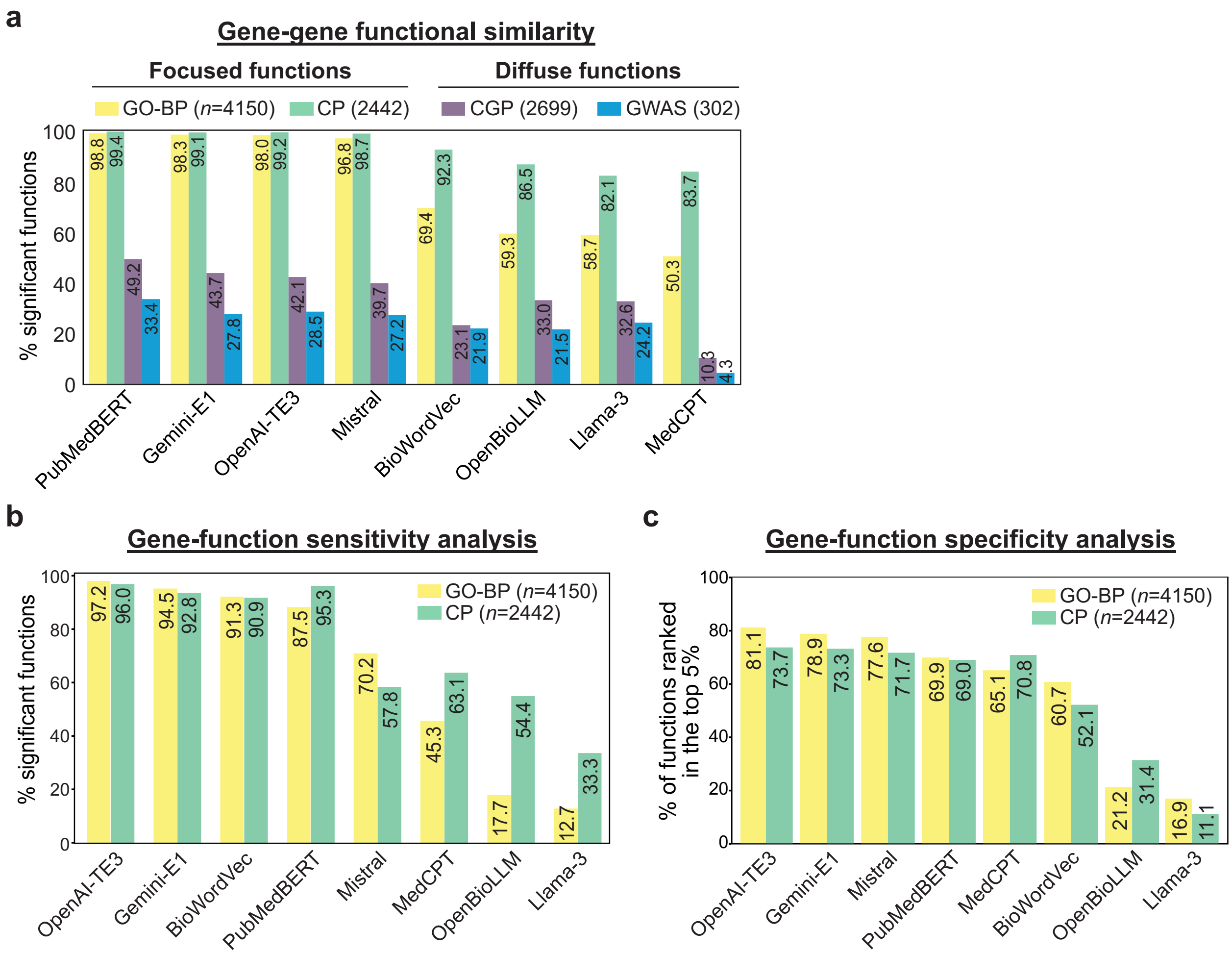

Supplementary Fig. 6. Performance of gene embeddings generated using NCBI gene summaries for GO-BP and CP functions. (a) Gene-gene functional similarity analysis, corresponding to Fig. 3b in the main text. Significance was defined as a one-tailed Mann-Whitney U test ($p < 0.05$) and $\delta \geq 0.147$. **(b)** Gene-function sensitivity analysis, corresponding to Fig. 4b. **(c)** Gene-function specificity analysis, corresponding to Fig. 5b. All analyses were performed identically to those shown in Figs. 3b, 4b, and 5b. Source data are provided as a Source Data file.

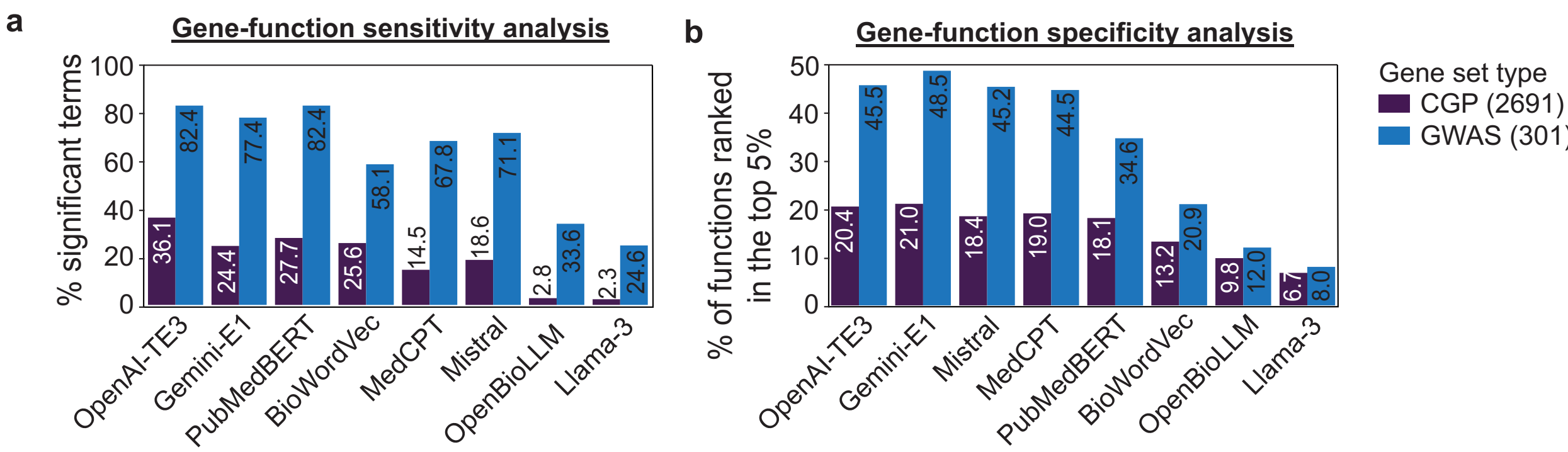

Supplementary Fig. 7. Gene-function sensitivity and specificity analyses for diffuse functional annotations. (a) Gene-function sensitivity analysis for CGP and GWAS gene sets, corresponding to Fig. 4b in the main text. Significance was defined as a one-tailed Mann-Whitney U test ($p < 0.05$) and $\delta \geq 0.147$. **(b)** Gene-function specificity analysis for CGP and GWAS gene sets, corresponding to Fig. 5b. All analyses were performed identically to those shown in Figs. 4b and 5b. Source data are provided as a Source Data file.

```
You are an efficient and insightful assistant to a molecular biologist.

<task>
Analyze the following set of proteins that have been identified as potentially
acting together in a particular biological context. Do their known roles and
relationships suggest that there a specific process (or processes) that may be
occurring? Do you see any strong relationships to specific disease processes or
other phenotypes? It's OK if you don't see a significant pattern. Don't try to find
one if it isn't there. In that case, just briefly describe the roles and
interactions.

Be concise and factual. Only speculate about novel protein functions or interactions
when you think there is very strong evidence. Be very clear when you are making a
conjecture, a speculation. Use language such as "it might be the case that
(speculation) based on (supporting reasoning)".

Be specific: Avoid overly general statements such as "the proteins are involved in
various cellular processes".
</task>

<process>
Identify groups of proteins with similar functions or which you think interact in
this context. For each group, briefly discuss their interplay, synergistic, or
antagonistic effects, and functional integration within the system, instead of
listing facts about individual proteins. Output no more than five groups. Do not
mention any specific protein or gene names in the output.

After completing your analysis, please also assign a self-assessment confidence
score to the process you selected. This score should range from 0.00 to 1.00. A
score of 0.00 indicates the lowest confidence, while 1.00 reflects the highest
confidence. This score helps gauge how accurately the chosen process name and the
generated process description represent the functions and activities within the
system of interacting proteins. When determining your score, consider the
proportion of genes in the protein system that participate in the identified
biological process. For instance, if you select "Ribosome biogenesis" as the process
name but only a few genes in the system contribute to this process, the score should
be lower compared to a scenario where a majority of the genes are involved in
"Ribosome biogenesis".
</process>


<output>
For each group, make sure to follow this output format: "(LLM self-assessed
confidence: ) Process name, Process description". Avoid using any special symbols
like the asterisk or the pound sign, or any highlighting. Don't generate a summary
paragraph at the end.
</output>

<protein>
{gene1, gene2, ...}
</protein>
```

Updated GSAI (web tool)
Original GSAI (published)
Added for this study

Supplementary Fig. 8. Prompt implementation for GSAI. We integrated elements from both the original (red) and updated (black) GSAI prompts and introduced additional instructions (blue).

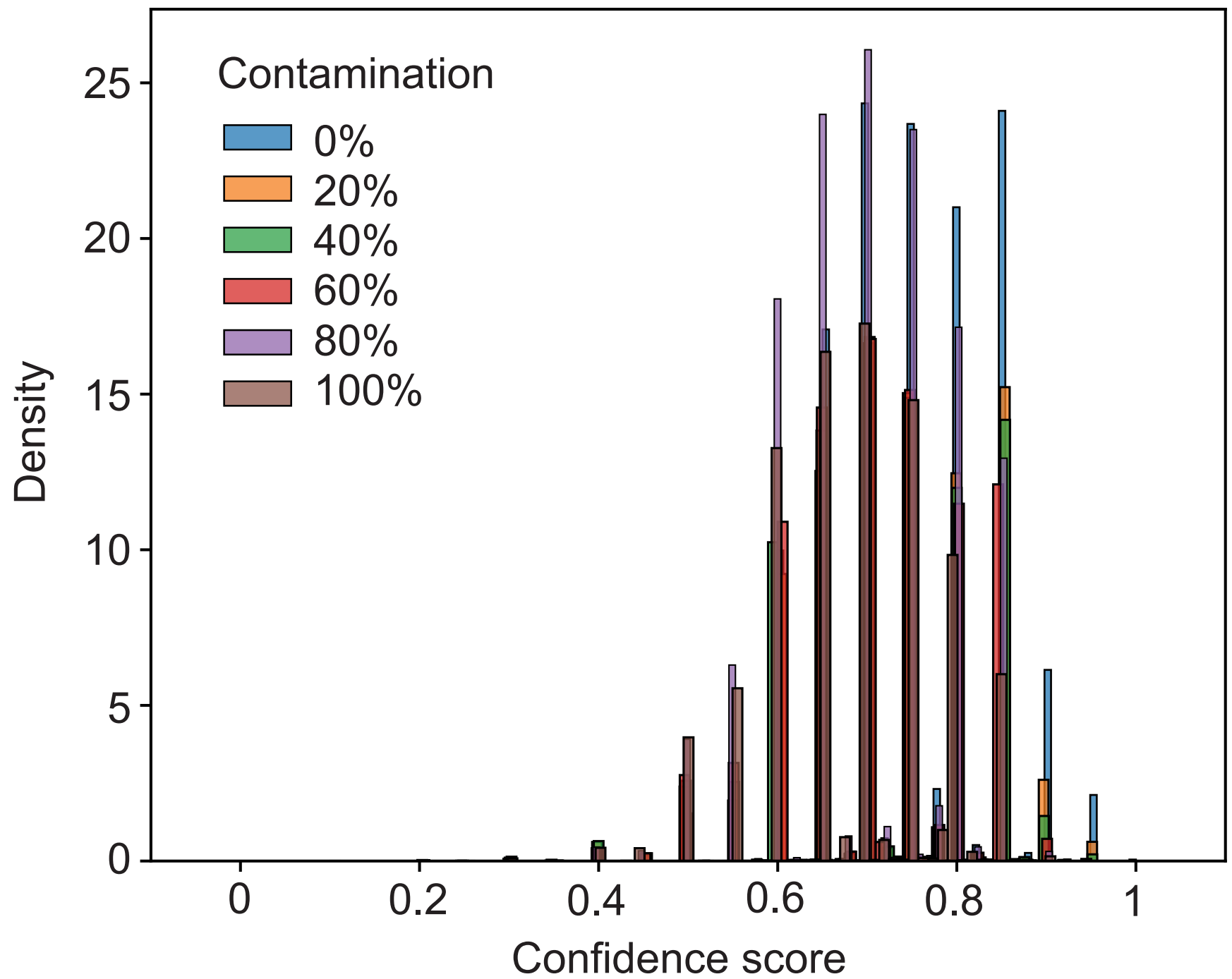

Supplementary Fig. 9. Distribution of LLM-generated confidence scores using GSAI for each predicted biological function. The scores exhibit a qualitative, quasi-discretized distribution with peaks at approximately 0.05-unit intervals across varying gene set contamination levels. Source data are provided as a Source Data file.

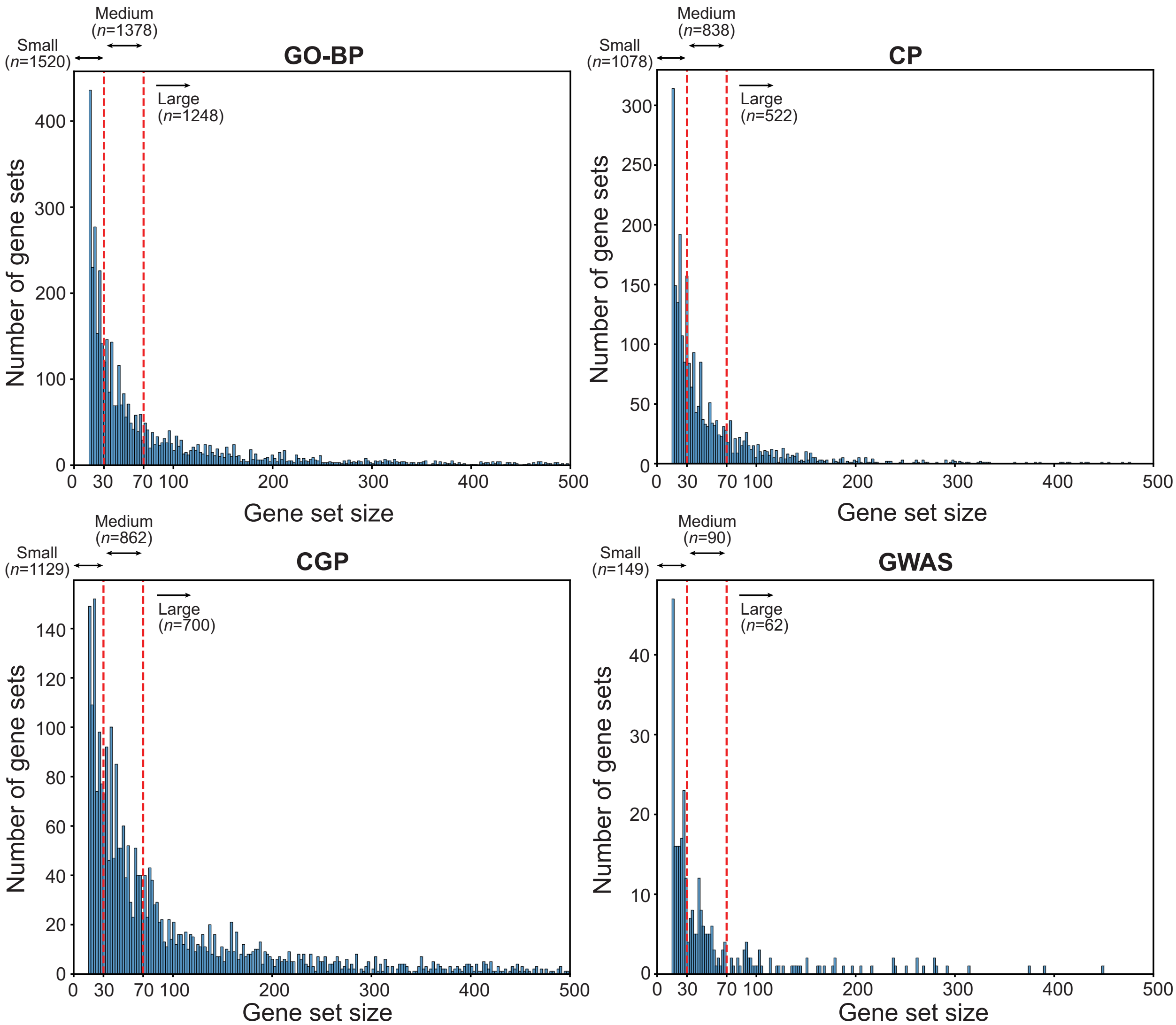

Supplementary Fig. 10. Distribution of gene set sizes across GO-BP, CP, CGP, and GWAS annotations. Gene set size thresholds were defined using the nearest 5-gene increment cutoffs to yield similarly sized subgroups in GO-BP. These thresholds were applied to the other three annotation sets to enable consistent comparisons. Source data are provided as a Source Data file.

```
You are an efficient and insightful assistant to a molecular biologist.

Given a biological pathway or gene function name: {gene_function}

Using ONLY your existing biomedical training knowledge (do NOT invent genes; do Not
include obscure or weakly supported genes), infer a biologically coherent gene set
and output the result using the EXACT format below.

Scientific constraints:
The gene set must contain real, official gene symbols (e.g., TP53)
Do NOT fabricate or guess gene names
Do NOT reduce the gene set to a minimal or illustrative subset. Aim for completeness
rather than minimality.
Genes must be biologically justified by known mechanisms, regulation, or pathway
structure
If the function/pathway is too vague, ill-defined, or non-specific, you MUST trigger
the failure mode

### OUTPUT FORMAT (STRICT)
Name: <concise gene set name>
LLM self-assessed confidence: <Low | Medium | High | None>
Analysis: <brief scientific explanation of why these genes belong together>
Gene set: <comma-separated official gene symbols>

Focus on:
Biological coherence
Mechanistic reasoning
Interpretability over completeness
Hallucination avoidance
```

Supplementary Fig. 11. Prompt used to generate gene sets representing specific biological functions for follow-up over-representation analysis (ORA).
