# Supplementary material for: An embedding-based framework enables statistical testing of gene-set function hypotheses inferred by large language models
Source: Nat Commun. 2026 Jul 30;17:9248. doi: 10.1038/s41467-026-75972-z (PMC13526818; doi:10.1038/s41467-026-75972-z)
Supplement: Supplementary file 2 [file 41467_2026_75972_MOESM2_ESM.pdf]

Description of Additional Supplementary Files

Supplementary Data 1. This file provides the complete annotation results for 107 gene embedding clusters generated from RAG-function gene embeddings. For each cluster, the table reports the representative keywords, GPT-4o-generated cluster annotation, cluster-specific unique keywords, the number of unique keywords, cluster size, compactness score, and the number of overlapping Gene Ontology Biological Process (GO-BP) terms identified using Jaccard index thresholds of 0.05 and 0.1.

Supplementary Data 2. This file provides model-specific specificity rankings for 1,000 GO-BP gene sets used to compare embedding-based methods with GSAI. Separate worksheets are provided for each embedding model. For each GO-BP term, the table reports the gene set size and the rank fraction of the corresponding function among all 11,943 GO-BP terms. A rank fraction below 0.05 indicates that the correct GO-BP term was ranked within the top 5% and was therefore considered specific in this analysis.

Supplementary Data 3. This file provides the detailed results for the representative GO-BP term "Postsynaptic Specialization Organization" used in the contamination analysis shown in Fig. 6c. The table reports the results at 0%, 50%, and 100% gene replacement, including the input gene list at each contamination level, GSAI-inferred function names and descriptions, GSAI confidence scores, and the corresponding embedding-based statistical testing results. Statistical significance and effect size were assessed using one-tailed Mann-Whitney U tests and Cliff's delta (δ), respectively. "PValue" denotes one-tailed Mann-Whitney U test *p*-value, and "DValue" denotes Cliff's delta.

Supplementary Data 4. This file provides the complete results for LLM-inferred *de novo* functional hypotheses across 50 NeST protein systems. For each NeST system, the table reports the NeST identifier, NeST annotation, gene list, GSAI-inferred function names and descriptions, GSAI confidence scores, GPT-4o-generated gene sets used for overrepresentation analysis (ORA), ORA *p*-values, MedCPT-based semantic similarity to the corresponding NeST annotation, and the corresponding embedding-based statistical testing results. "EmbeddingPValue" denotes one-tailed Mann-Whitney U test *p*-value, and "EmbeddingDValue" denotes Cliff's delta.
