# Supplementary material for: An embedding-based framework enables statistical testing of gene-set function hypotheses inferred by large language models
Source: Nat Commun. 2026 Jul 30;17:9248. doi: 10.1038/s41467-026-75972-z (PMC13526818; doi:10.1038/s41467-026-75972-z)
Supplement: Supplementary file 3 [file 41467_2026_75972_MOESM7_ESM.pdf]

nature portfolio

Corresponding author(s): Yu-Chiao Chiu

Last updated by author(s): Jun 30, 2026

Reporting Summary

Nature Portfolio wishes to improve the reproducibility of the work that we publish. This form provides structure for consistency and transparency in reporting. For further information on Nature Portfolio policies, see our Editorial Policies and the Editorial Policy Checklist.

Statistics

For all statistical analyses, confirm that the following items are present in the figure legend, table legend, main text, or Methods section.

n/a	Confirmed	
[ ]	[x]	The exact sample size (*n*) for each experimental group/condition, given as a discrete number and unit of measurement
[ ]	[x]	A statement on whether measurements were taken from distinct samples or whether the same sample was measured repeatedly
[ ]	[x]	The statistical test(s) used AND whether they are one- or two-sided<br>*Only common tests should be described solely by name; describe more complex techniques in the Methods section.*
[x]	[ ]	A description of all covariates tested
[ ]	[x]	A description of any assumptions or corrections, such as tests of normality and adjustment for multiple comparisons
[ ]	[x]	A full description of the statistical parameters including central tendency (e.g. means) or other basic estimates (e.g. regression coefficient) AND variation (e.g. standard deviation) or associated estimates of uncertainty (e.g. confidence intervals)
[ ]	[x]	For null hypothesis testing, the test statistic (e.g. *F*, *t*, *r*) with confidence intervals, effect sizes, degrees of freedom and *P* value noted<br>*Give P values as exact values whenever suitable.*
[x]	[ ]	For Bayesian analysis, information on the choice of priors and Markov chain Monte Carlo settings
[x]	[ ]	For hierarchical and complex designs, identification of the appropriate level for tests and full reporting of outcomes
[ ]	[x]	Estimates of effect sizes (e.g. Cohen's *d*, Pearson's *r*), indicating how they were calculated

Our web collection on statistics for biologists contains articles on many of the points above.

Software and code

Policy information about availability of computer code

Data collection

Gene descriptions from NCBI were retrieved using custom Python scripts in Python v3.11.7 through the Entrez API implemented in Biopython v1.84. Literature-derived gene descriptions were retrieved using the LitSense API and summarized using GPT-4o model gpt-4o-2024-08-06. The full data collection source code will be made publicly available via Zenodo (https://doi.org/10.5281/zenodo.21044582).

Data analysis

Major data analysis was performed using Python (v3.11.7), and the full source code will be made publicly available via Zenodo (https://doi.org/10.5281/zenodo.21044582). The proposed computational framework consists of three main components: (1) embedding generation, (2) similarity computation, and (3) statistical analysis. For the embedding generation, we employed different models:
1. text-embedding-3-large (https://platform.openai.com/docs/libraries)
2. gemini-embedding-001 (https://ai.google.dev/gemini-api/docs/embeddings)
3. e5-mistral-7b-instruct (https://huggingface.co/intfloat/e5-mistral-7b-instruct)
4. pubmedbert-base-embeddings (https://huggingface.co/NeuML/pubmedbert-base-embeddings)
5. MedCPT-Article-Encoder (https://huggingface.co/ncbi/MedCPT-Article-Encoder)
6. OpenBioLLM-70B (https://huggingface.co/mradermacher/Llama3-OpenBioLLM-70B-GGUF)
7. Llama-3-70B (https://ollama.com/library/llama3:70b)
8. BioWordVec (https://github.com/ncbi-nlp/BioSentVec)
9. MedCPT Query Encoder (https://huggingface.co/ncbi/MedCPT-Query-Encoder)

Embeddings from the text-embedding-3-large and gemini-embedding-001 were obtained via API. The remaining six models were downloaded from the Hugging Face or GitHub and executed locally. Of these, OpenBioLLM-70B and Llama-3-70B were executed locally using the Ollama framework. Similarity computation and statistical analysis was performed using the following Python libraries:
biopython (v1.84, https://biopython.org/)
fasttext (v0.9.3, https://pypi.org/project/fasttext/)

google-generativeai (v0.7.2, https://pypi.org/project/google-generativeai/)
numpy (v1.26.4, https://pypi.org/search/?q=numpy)
matplotlib (v3.8.2, https://pypi.org/search/?q=matplotlib&o=)
ollama (v0.2., https://pypi.org/project/ollama/)
openai (v1.23.2, https://pypi.org/project/openai/)
pandarallel (v1.6.5, https://pypi.org/project/pandarallel/)
pandas (v2.2.0, https://pypi.org/project/pandas/)
pillow (v10.2., https://pypi.org/project/pillow/)
pronto (v2.5.7, https://pypi.org/project/pronto/)
requests (v2.32.3, https://pypi.org/project/requests/)
scikit-learn (v1.4.0, https://pypi.org/project/scikit-learn/)
scipy (v1.12.0, https://pypi.org/project/scipy/)
seaborn (v0.13.2, https://pypi.org/project/seaborn/)
sentence-transformers (v3.0.1, https://pypi.org/project/sentence-transformers/)
spacy (v3.7.5, https://pypi.org/project/spacy/)
torch (v2.3.1, https://pypi.org/project/torch/)
transformers (v4.41., https://pypi.org/project/transformers/)
venn (v0.1.3, https://pypi.org/project/venn/)
In the applications of this study, we utilized GPT-4o (model: gpt-4o-2024-08-06) via OpenAI's API for generating hypothesis. Prompts were adapted from the GSAI (https://idekerlab.ucsd.edu/gsai/) with minor modifications. The modified prompt designs and usage scripts are available via Zenodo (https://doi.org/10.5281/zenodo.21044582).

For manuscripts utilizing custom algorithms or software that are central to the research but not yet described in published literature, software must be made available to editors and reviewers. We strongly encourage code deposition in a community repository (e.g. GitHub). See the Nature Portfolio guidelines for submitting code & software for further information.

Data

Policy information about availability of data

All manuscripts must include a data availability statement. This statement should provide the following information, where applicable:

- Accession codes, unique identifiers, or web links for publicly available datasets
- A description of any restrictions on data availability
- For clinical datasets or third party data, please ensure that the statement adheres to our policy

All data used in this study are publicly available. Processed datasets generated and analyzed in this study can be made available via Zenodo (https://doi.org/10.5281/zenodo.21044582) upon publication. The R package implementing the present analysis is available on GitHub (https://github.com/chiu-lab/DEGEmbedR). Raw data were obtained from the following public databases:
1. NCBI (v2024.2): gene descriptions were retrieved using Python scripts (v3.11.7) via the Entrez API (biopython(v1.84), https://biopython.org/).
2. Alliance of Genome Resources (v2024.5): https://www.alliancegenome.org/downloads#gene-descriptions
3. UniProt (v2024.5): https://ftp.uniprot.org/pub/databases/uniprot/current_release/knowledgebase/taxonomic_divisions/
4. Biological Process (GO-BP, v2023.2.Hs, v2025.1.Hs), Canonical Pathway (CP, v2023.2.Hs, v2025.1.Hs), and Chemical and Genetic Perturbations (CGP, v2023.2.Hs, v2025.1.Hs): https://www.gsea-msigdb.org/gsea/downloads_archive.jsp
5. GWAS Catalog: https://www.ebi.ac.uk/gwas/docs/file-downloads
6. Gene Ontology Biological Process: https://github.com/idekerlab/llm_evaluation_for_gene_set_interpretation/blob/main/data/go_terms.csv
7. 1000 selected GO-BP terms: https://github.com/idekerlab/llm_evaluation_for_gene_set_interpretation/blob/main/data/GO_term_analysis/1000_selected_go_terms.csv
8. NeST50: https://github.com/ncbi-nlp/GeneAgent/tree/main/Datasets/NeST
9. LINCS: Supplementary Table 3 (Hu et al. Nature Methods 2024. https://doi.org/10.1038/s41592-024-02525-x)

Research involving human participants, their data, or biological material

Policy information about studies with human participants or human data. See also policy information about sex, gender (identity/presentation), and sexual orientation and race, ethnicity and racism.

Reporting on sex and gender	Not applicable.
Reporting on race, ethnicity, or other socially relevant groupings	Not applicable.
Population characteristics	Not applicable.
Recruitment	Not applicable.
Ethics oversight	Not applicable.

Note that full information on the approval of the study protocol must also be provided in the manuscript.

Field-specific reporting

Please select the one below that is the best fit for your research. If you are not sure, read the appropriate sections before making your selection.

☒ Life sciences ☐ Behavioural & social sciences ☐ Ecological, evolutionary & environmental sciences

For a reference copy of the document with all sections, see nature.com/documents/nr-reporting-summary-flat.pdf

Life sciences study design

All studies must disclose on these points even when the disclosure is negative.

Sample size

No wet-lab experiments were performed in this study. For gene descriptions, we included all human protein-coding genes available in the NCBI database (n = 18,288-18,723). Functional annotations were obtained from four curated collections: Gene Ontology – Biological Process (GO-BP, n=4,146), Canonical Pathway (CP, n=2,438), Chemical and Genetic Perturbations (CGP, n=2,691), and GWAS Catalog (disease trait, n=301). To avoid overly specific or generalized functional terms, we included only gene sets containing between 15 and 500 genes, following the default setting for the Gene Set Enrichment Analysis (GSEA). Additionally, a curated set of 1,000 GO terms was selected for performance evaluation and comparison with a previous publication (Hu et al. Nature Methods 2024. https://doi.org/10.1038/s41592-024-02525-x). In real-world application, NeST50 was obtained from the GitHub repository (https://github.com/ncbi-nlp/GeneAgent/tree/main/Datasets/NeST).

Data exclusions

No data were excluded after applying the predefined inclusion criteria.

Replication

No wet-lab experiments were performed in this study. As detailed above, all data, source code, and selected intermediate results are provided. These resources allow for complete replication of the study's computational procedures and results.

Randomization

No wet-lab experiments were performed in this study. Randomization was applied in the hypothesis-free application of the study. Specifically, we introduced contamination to each of the 4,146 gene sets from the Gene Ontology – Biological Process (GO-BP) by replacing genes with randomly sampled genes. The contamination levels ranged from 0% (the original gene set) to 100% (fully randomized gene set) with 5% increments.

Blinding

Not applicable to this computational study, as there was no group allocation or experimental data collection.

Reporting for specific materials, systems and methods

We require information from authors about some types of materials, experimental systems and methods used in many studies. Here, indicate whether each material, system or method listed is relevant to your study. If you are not sure if a list item applies to your research, read the appropriate section before selecting a response.

Materials & experimental systems

n/a	Involved in the study	
☒	☐	Antibodies
☒	☐	Eukaryotic cell lines
☒	☐	Palaeontology and archaeology
☒	☐	Animals and other organisms
☒	☐	Clinical data
☒	☐	Dual use research of concern
☒	☐	Plants

Methods

n/a	Involved in the study	
☒	☐	ChIP-seq
☒	☐	Flow cytometry
☒	☐	MRI-based neuroimaging

Plants

Seed stocks

Not applicable.

Novel plant genotypes

Not applicable.

Authentication

Not applicable.
