# Supplementary material for: An embedding-based framework enables statistical testing of gene-set function hypotheses inferred by large language models
Source: Nat Commun. 2026 Jul 30;17:9248. doi: 10.1038/s41467-026-75972-z (PMC13526818; doi:10.1038/s41467-026-75972-z)
Supplement: Supplementary file 4 [file 41467_2026_75972_MOESM8_ESM.pdf]

nature portfolio

Peer Review File

An embedding-based framework enables statistical testing of gene-set function hypotheses inferred by large language models

Corresponding Author: Dr Yu-Chiao Chiu

This file contains all reviewer reports in order by version, followed by all author rebuttals in order by version.

Version 0:

Reviewer comments:

Reviewer #1

(Remarks to the Author)
In this work authors evaluate embeddings of genes and biological functions derived from state-of-the-art LLM models to assess their biological relevance, specificity, and interpretability. The benchmark evaluates their ability to capture gene-gene functional similarities and gene-function relationships across diverse biological contexts.
Authors state that this study demonstrates the potential of LLM-derived embeddings to enhance quantitative functional representation and facilitate hypothesis testing of gene functions towards improving the reliability and practical applicability of LLMs in functional genomics and computational biology.
The framework presented adds a critical statistical layer for evaluating LLM-inferred gene functions, enabling hypothesis testing without predefined gene sets and enhancing the reliability
of LLM-guided functional discovery.

This study presents the first systematic and statistical evaluation of LLM-based embedding models for gene function representation. It a very valuable work, with an important amount of experimetal work involved. The study is original, very complete and comprehensive, and it is certainly of interest to the community.

I have just some minor comments in order to further improve the work:
- The data selected for the experiments is quite reduced, considering that there are millions of proteins in UniProt. Why just choosing less than 20,000 genes? The data selection should be better justified.
- I do not understand the objective of the data ablation in Fig 2d. What should be expected form such experiment? that the removal of a term should impact significantly in the embedding? if that is the case a 5% reduction is not really a big reduction, isn't it? On the other hand, one could also expect that a good embedding should not be so sentitive to a word removal. This experiment should be better explained.
- Why ablation was not performed with all LLM methods evaluated?
- Why only k-means for clustering and why k=100? I think this should be better justified. Or even better, several clustering algorithms could be tried and results compared in order to see if conclusions still hold.

(Remarks on code availability)

Reviewer #2

(Remarks to the Author)
Paper Title:
Large language model embeddings enable quantitative gene function representation and hypothesis testing
Summary:
This paper recognized a limitation of traditional functional annotation, which relies on human-curated and static databases that limit the opportunity to capture novelty and dynamics of gene functions. To address this, the authors developed a framework that uses LLM-generated embeddings from gene function descriptions and quantitatively evaluated the gene-gene and gene-function relationships derived from the embeddings for biological relevance. Their evaluation approach

involves 1) an ablation study to find the key word for gene function alignment, 2) the systematic evaluation of sensitivity and specificity for the functional representation from the embedding space, 3) hypothesis testing for real-world gene sets not previously documented, 4) statistical testing for gene function hypotheses, overcoming the lack of significance scoring in an existing LLM-based gene set functional annotation tool. The key findings claimed by the paper include that the text embeddings achieved high sensitivity and specificity for predefined gene functions. Their framework is heavily dependent on statistical analysis, but the authors did not justify the use of statistical methods, and the significant scores may be inflated due to unmet assumptions. Additionally, the main impact and real-world applications of hypothesis testing are not clearly outlined, and the embedding approach relies on predefined gene function descriptions from curated databases. This dependency leaves it unclear how the approach addresses the stated limitation of over-reliance on static resources. The traditional functional enrichment approach can already provide statistical results and return terms ranked by the significance of overlap between gene lists. In contrast, the statistical score proposed by this study seems less intuitively interpretable, as it depends on the significance of the separation between inferred distributions.
Concerns:
The abstract needs to highlight the key findings from the paper, whereas it currently reads more like a description of the methods than a summary of the findings. The impact and practicality are also not highlighted in the abstract.

There is a significant gap between motivation and implementation. The authors point out that the limitation of functional annotation is that it depends on databases that are manually curated and static. However, their LLM-based approach embeds pre-defined gene function descriptions from static databases such as NCBI, making it unclear how their approach addresses this issue. Furthermore, functional enrichment can be performed using gene sets annotated from experimental data, such as GWAS catalogs or gene expression signatures from transcriptomics data, which can capture some context-specificity. However, the paper did not consider how the embedding approach could adapt to this.

Basing the method on the embedding of curated gene function descriptions makes it heavily dependent on common genes that are well-studied and well-documented. This mirrors the structure of the curated gene function databases. The author's methods have limitations in discovering new gene/protein functions, especially for understudied proteins. Those are more impactful challenges to overcome in biology and would be of greater interest. Can the authors comment and address this limitation?

The authors grouped gene function embeddings into 100 clusters and then annotated them using LLM. How do those clusters compare with existing gene clusters from known databases? If there are differences, are there new findings? (i.e., new genes being clustered in a well-studied gene set group or a new functional term). It would strengthen the paper if they compared cluster composition to known biological clusters.

The authors did not seem to review the keywords used for the clustering, as there are multiple obvious errors: 1) keywords are dominated by non-biological terms like "predicted" and "located" and very general biological terms like transcript, variant, and encoding, 2) they include semantic duplicates such as "olfactory" vs. "Olfactory", "enzyme" vs. "enzymes", and 3) biological terms were separated into individual words, such as "RNA Polymerase II" → "RNA", "Polymerase", "II". The authors should consider the method that extracts the biological terms from the functional description instead of splitting the word.

What is the motivation for the analysis of inter-model similarity in gene clusters? There is no clear conclusion for the comparison between different embedding models.

There are substantial concerns about the statistical methods the authors chose to calculate the significance. Take the analysis for the gene-gene similarity as an example: Is the size of inter-function similarities the same as intra-function similarities? The one-tailed t-tests asked the question whether the mean similarity of the intra-function group is larger than the inter-function group. However, n number of genes can result in $n*(n-1)/2$ intra-function pairs that are not completely independent observations and may therefore lead to very significant results with small mean differences. The authors did not justify the assumption for choosing to use t-test over other non-parametric tests, such as the Wilcoxon rank-sum test, which does not assume independence. The effect size (the difference between means) should be taken into consideration when determining significance (for example, $p < 0.05$ and mean difference > X). Similar statistical analysis concerns applied to all related tasks described in the paper.

The hypothesis testing task described in the paper is trivial and only confirmatory. For example, the STING pathway is already predefined as a gene set of interest to explore, and the test is to check whether related genes are enriched in the visugromab-treated DEGs. There is a gap in the logic: how is the STING pathway being recognized without knowing genes that are associated with it, and if the hypothesis itself is already based on that gene list, why is this hypothesis testing task needed? Can the embedding method be used to explore new/alternative pathways or mechanisms for the given observation? How would the authors expect researchers to use this method for real-world applications without prior knowledge of the STING pathway? What is the unique information from this approach that can help refine potential follow-up studies for the STING pathway and Visugromab mechanism, other than some p-value?

For the MOA hypotheses, how many MOAs were identified as significant in the study? According to Figure 6c, all the MOAs tested in the study are significant (i.e., even the last in the figure is highly significant). This raises concerns about whether the embedding method can discriminate between more or less relevant mechanisms. In addition, there is no gene set information reported that leads to the significance of the MOA ranking. It is unclear whether this approach is to differentiate the specific associations or just generic signals. And, beyond that, the purpose of this step is also unclear. What information is added, given that the calcium signaling is already identified in the drug-target network.

Both traditional functional enrichment analysis and this LLM embedding approach provide metrics of significance, but how do the significance and top enriched functional terms differ between the two methods? Do the methods yield substantively different results that would affect the insight gained by a researcher exploring a gene set? Again, given that the author's approach also relies on curated gene function descriptions in a canonical database, the gain of information using this approach is not clear. Since it is faster and cheaper to use functional enrichment, what are the benefits of using LLM embeddings?

For the comparison with GSAI's confidence score, is the 0 confidence being considered in the distribution? Zeros were reported from the original paper, but not shown in the Supplementary Fig. 6. Are there no zeros being returned, or are the authors excluding them? If excluding, please provide the rationale.

(Remarks on code availability)
Given the concerns that we raised, it did not seem meaningful to review the code.

Reviewer #3

(Remarks to the Author)
This manuscript presents a computational framework to evaluate and apply large language model (LLM)-derived embeddings for gene function representation and hypothesis testing. The authors benchmark multiple LLMs using gene- and function-level annotations from public databases and demonstrate two applications related to drug mechanism discovery. The manuscript explores a timely and promising application of large language models (LLMs) in gene function representation. However, the technical depth and conceptual advancement remain limited.
Major Concerns
1. Curated Summary Inputs Limits the Utility and Innovation of the LLM-based Framework
While the authors present a framework to compare gene and function embeddings across multiple LLMs, their exclusive use of curated summaries (e.g., from NCBI Gene) limits the linguistic richness and contextual diversity of the input. These structured, shallow texts do not fully leverage LLMs' strength in extracting relational and contextual knowledge from unstructured language. As a result, applying off-the-shelf LLM embeddings to such input offers limited advantage over traditional methods (e.g., Word2Vec, BioBERT) and weakens the motivation for using LLMs in this setting. The limited input prevents these models from showcasing their semantic capabilities, making cross-model comparisons less informative. Moreover, the curated summaries used as input often share substantial content overlap with benchmark annotation sources such as Gene Ontology (GO). This overlap introduces a risk of information leakage, where the inputs and evaluation metrics are not fully independent. As a result, it becomes difficult to assess the true semantic performance of the models, weakening the interpretability and rigor of the benchmarking process.
A more meaningful application of LLMs would be to analyze large-scale unstructured literature (e.g., PubMed abstracts or full texts), where their semantic reasoning capabilities could be more fully utilized. As it stands, the framework's reliance on curated summaries limits both its conceptual novelty and practical significance, falling short of the innovation threshold expected for publication in Nature Communications.
2. Limited accessibility and translational utility
The framework lacks a deployable implementation, such as a web-based interface or R/Python package, making it difficult for other researchers to apply the method in practice. This limits its utility and broader impact. I strongly encourage the authors to develop a user-accessible implementation to support adoption and reproducibility.

(Remarks on code availability)

Reviewer #4

(Remarks to the Author)
I co-reviewed this manuscript with one of the reviewers who provided the listed reports. This is part of the Nature Communications initiative to facilitate training in peer review and to provide appropriate recognition for Early Career Researchers who co-review manuscripts.

(Remarks on code availability)

Reviewer #5

(Remarks to the Author)
I co-reviewed this manuscript with one of the reviewers who provided the listed reports. This is part of the Nature Communications initiative to facilitate training in peer review and to provide appropriate recognition for Early Career Researchers who co-review manuscripts.

(Remarks on code availability)

Version 1:

Reviewer comments:

Reviewer #1

(Remarks to the Author)
Authors have satisfactorily responded to all my comments. Thanks.

(Remarks on code availability)

Reviewer #2

(Remarks to the Author)
Comments:

We appreciate the authors for making updates to the keyword extraction; we agree that the resulting keywords are now better suited for clustering. Thanks to the authors for their efforts in revising the analyses to use non-parametric statistical tests. The study now presents a good systematic evaluation framework for future similar gene/ function-embedding tasks.

Our greatest concern remains:

The authors have not adequately addressed our comments regarding the intended uses of the method and the need for benchmarking versus gene set enrichment, the dominant method in the field. There seems to be a misunderstanding of our concerns.

The two examples of hypothesis testing are anecdotal rather than systematic and do not compare the results to an enrichment approach, see below. There is also a lack of systematic quantitative comparison of how results differ from those of traditional enrichment analysis over a test corpus. The critical point is to provide evidence characterizing how the technique yields different results across a broad range of data. Furthermore, the insights provided to a researcher by their method should be clearly compared with those from enrichment analysis.

In the STING and visugromab example, the authors first proposed the novel hypothesis that STING mediates visugromab-induced immune sensitization and that similarity between visugromab-induced genes and a GPT-4o-curated description of the STING pathway would support this hypothesis. But what is the difference between asking the LLM to generate a text description and asking it to curate a gene list? The authors could have asked GPT-4o to generate a ‘curated’ gene set for the STING pathway, add to the reference corpus, and run the enrichment analysis?

In the vortioxetine example, the authors first developed the novel hypothesis that vortioxetine acts via calcium channels in glioblastoma, then curated the MOA description embeddings using GPT-4o. Similarly, the authors could have asked the LLM to curate the gene list related to the MOA and run the enrichment analysis. Then compare their approach with the enrichment analysis.

Note that the purpose of hypothesis testing is to falsify unlikely conditions. The author's current hypothesis testing scenarios lack negative controls. For example:

The authors did not compare the similarity of the STING description to comparable GPT-4o-generated descriptions of other immune pathways. GDF-15 may have multiple modes of action; can the STING pathway be meaningfully differentiated from alternatives? If the authors can show the DEG embeddings are much closer to STING than other pathways, the hypothesis is more likely to be true.

Their MOA analysis found calcium/ion channel regulation as the top “hit”, but are there negative controls in the analysis? What MOAs should not be associated with vortioxetine? Are the distances of gene embeddings against negative controls larger than the expected positive controls? Please consider including these results in the analysis.

Notably, the promise of the method is that it can make use of non-curated information. But in the current paper, they focus on the use of curated information, similar to enrichment analysis, with RAG left as a “promising approach”.

Finally, in the discussion, pg. 19 line 396-397: “Notably, our framework produced promising findings such as the visugromab-STING link and alternative signaling of vortioxetine…” is too strong without addressing the comments raised above. It reads like this study developed and validated the hypotheses, but the authors developed novel hypotheses outside their framework.

Other issues:

Thanks to the authors for updating the abstract, including more details of key findings and an overview of the study. The first two sentences, however, do not yet identify the key gap and how LLMs can help.

Fig. 2a needs a legend divider to explicitly show that the top three are the same genes across databases and the bottom three are different genes in the same database.

Page 7 line 123-125 authors stated “Functionally related clusters emerged in close proximity…”, but there is no data or result showing the distances between clusters. The t-SNE analysis only preserves local relationships, not the global distances, so this statement was not supported. Suggest providing supporting evidence or removing the claim in the result.

Intra-cluster gene-gene cosine similarity was first mentioned in line 139 on pg.7. Without context, it is hard to interpret what 0.75 means in relation to other clusters (i.e., does that mean it is a really tight cluster or around medium). Suggest providing a distribution plot of all intra-cluster cosine similarities to support the number; otherwise, this number here solely does not provide much information to understand the cluster (it can be moved to the ablation analysis paragraph as a parentheses, i.e., dropped X % from 0.75 to X).

Line 219-220 on pg. 11 said the CGP description has limited functional coherence as it emphasized perturbation and experimental conditions. Is it really a limit? It is important to identify genes that behave similarly under certain conditions, as context matters. The result should be addressed differently.

Fig. 4d is not referenced in the text.

Line 301 on pg.15: Are there any citations that support the potential new MOA? Something in a different context? Skeptical if it is a purely new concept proposed without any external evidence.

Thanks to the authors for making changes and providing the full range of scores. The statistical score reported in the manuscript is promising. However, when I tested GSAI using the same model (gpt-4o-2024-08-06) on 100 random gene sets, it returned 94 zero scores. Therefore, a nontrivial number of zeros is expected in the score distribution. To help interpret and reproduce these results, the authors should consider rerunning the analysis and double-checking the result, or provide the data and code to enable us to reproduce the analysis.

Comments on the code:

The code executes successfully.

It needs more detailed documentation for the required input and the optional input. For example, what are the background genes? If the researcher wanted to learn about a list of DEGs, would they be required to provide another list of background genes?

(Remarks on code availability)

Reviewer #3

(Remarks to the Author)
We appreciate the extensive revisions and the substantial effort invested in expanding the analyses in this version. The manuscript is now more complete from a technical perspective. However, the major conceptual concerns raised in the previous round remain unresolved.
1. A central issue is that the framework continues to rely heavily on curated, highly structured gene summaries. Although RAG-generated summaries and LLM-generated descriptions were included in the revision, they do not materially improve performance or address the core dependence on curated text. As a consequence, the method still primarily reflects the information structure already encoded in these curated descriptions.
2. The input data still share substantial lexical and semantic overlap with the GO and pathway annotations used for benchmarking. This lack of independence between input and evaluation raises concerns about information leakage and makes it difficult to determine whether the reported performance reflects genuine semantic capability of LLM embeddings rather than the inherent structure of curated annotations.
3. The conceptual novelty over traditional ontology- or enrichment-based approaches remains limited. Although the embedding framework provides some incremental insights, these gains are modest—for example, only 7 of 103 clusters (6.8%) do not overlap with any GO term, and the MOA case study primarily highlights broad pathways that conventional DEG or network analyses would also recover. These observations suggest that the embedding representations, while useful, do not fundamentally alter the types of biological insights that can be obtained using existing methods.
Overall, while the technical revisions improve the manuscript, the central issues remain, and the conceptual novelty of the proposed framework is still insufficient for Nature Communications.

(Remarks on code availability)

Reviewer #4

(Remarks to the Author)
I co-reviewed this manuscript with one of the reviewers who provided the listed reports. This is part of the Nature Communications initiative to facilitate training in peer review and to provide appropriate recognition for Early Career Researchers who co-review manuscripts.

(Remarks on code availability)
Given the concerns that we raised, it did not seem meaningful to review the code.

Reviewer #5

(Remarks to the Author)
I co-reviewed this manuscript with one of the reviewers who provided the listed reports. This is part of the Nature Communications initiative to facilitate training in peer review and to provide appropriate recognition for Early Career Researchers who co-review manuscripts.

(Remarks on code availability)

Version 2:

Reviewer comments:

Reviewer #2

(Remarks to the Author)
This revision provides a substantial improvement over the previous version. We appreciate the author's serious effort in addressing the critiques from the last review. The narrative is now clearer, the foundation is stronger, and the conceptual contribution is better positioned. We detail below what has been addressed well, followed by the remaining points.

One concern in the previous round was the lack of a quantitative comparison with gene set enrichment. The authors have now rigorously implemented this across 50 NeST protein systems, showing that GO-BP rankings from the embedding framework achieve substantially higher concordance with the semantic reference ranking than those from ORA.

Another critical concern the authors addressed was the use of anecdotal examples in hypothesis testing. This version has removed the two examples and shifted the theme from hypothesis testing to a more systematic evaluation of the statistical output from their method. This change has made their method more practical and the narrative clearer.

In addition, the contamination analysis has addressed the concern raised about the lack of negative controls.

The RAG pipeline has been expanded to include four context-specific query types: function, pathway, perturbation, and disease. This addresses the concern that RAG was previously treated as a secondary or future direction.

Major concerns:

The authors attribute lower performance on GWAS and CGP gene sets to the heterogeneous nature of complex disease biology, and leave it at that. This framing treats heterogeneity as a caveat rather than recognizing it as the most scientifically interesting challenge for this framework. The idea of using LLM-based approaches is that LLMs could go beyond what curated databases already offer. The context-specific RAG descriptions with disease-specific retrieval suggest the authors are already moving in this direction. The Discussion should explicitly state this, framing context-dependent gene function as a forward-looking opportunity rather than a limitation.

A clear schematic should be included in Figure 7 that details the approach the authors used for function inference and comparisons.

The semantic reference ranking in Figure 7 is constructed using MedCPT embeddings, while MedCPT is simultaneously one of the seven evaluated models. The authors should briefly justify this design choice and clarify that it does not affect the central OpenAI-TE3 vs. ORA comparison.

The ORA used in the NeST evaluation is performed on GPT-4o-generated gene sets rather than the standard enrichment workflow using curated database gene sets. This should be labeled clearly. And please clarify why standard enrichment approaches were not used to find enriched terms for the NeST gene sets.

Minor concerns:

The de novo functional inference (Fig. 7b) showed a sample size of 36 instead of the 50 gene sets from the original pool. Is the missing gene set due to a lack of functional enrichment/GSAI function proposal? Or other reason, please clarify.

(Remarks on code availability)

Reviewer #3

(Remarks to the Author)
The manuscript has been substantially improved at the technical level, with expanded benchmarking and clearer positioning as a validation framework. However, the manuscript does not demonstrate that the method provides a necessary or decision-altering capability within typical functional genomics workflows (such as Gene Ontology). In particular, the current analyses primarily benchmark different LLM models under their own framework with existing annotations (GO) rather than demonstrating a distinct capability beyond established approaches. As such, the contribution appears to be an incremental refinement of existing approaches rather than a conceptual or practical advance. Therefore, I still feel that the manuscript does not meet the standard for novelty in Nature Communications.

(Remarks on code availability)

Reviewer #4

(Remarks to the Author)
I co-reviewed this manuscript with one of the reviewers who provided the listed reports. This is part of the Nature Communications initiative to facilitate training in peer review and to provide appropriate recognition for Early Career Researchers who co-review manuscripts.

(Remarks on code availability)

Reviewer #5

(Remarks to the Author)
I co-reviewed this manuscript with one of the reviewers who provided the listed reports. This is part of the Nature Communications initiative to facilitate training in peer review and to provide appropriate recognition for Early Career Researchers who co-review manuscripts.

(Remarks on code availability)

Version 3:

Reviewer comments:

Reviewer #2

(Remarks to the Author)
The authors have addressed our concerns.

(Remarks on code availability)

Reviewer #5

(Remarks to the Author)
I co-reviewed this manuscript with one of the reviewers who provided the listed reports. This is part of the Nature Communications initiative to facilitate training in peer review and to provide appropriate recognition for Early Career Researchers who co-review manuscripts.

(Remarks on code availability)

Open Access This Peer Review File is licensed under a Creative Commons Attribution 4.0 International License, which permits use, sharing, adaptation, distribution and reproduction in any medium or format, as long as you give appropriate credit to the original author(s) and the source, provide a link to the Creative Commons license, and indicate if changes were made.
In cases where reviewers are anonymous, credit should be given to 'Anonymous Referee' and the source.
The images or other third party material in this Peer Review File are included in the article's Creative Commons license, unless indicated otherwise in a credit line to the material. If material is not included in the article's Creative Commons license and your intended use is not permitted by statutory regulation or exceeds the permitted use, you will need to obtain permission directly from the copyright holder.
To view a copy of this license, visit https://creativecommons.org/licenses/by/4.0/

Manuscript #: NCOMMS-25-46979A
Title: Large language model embeddings enable quantitative gene function representation and hypothesis testing

Overall Response: We sincerely thank all five reviewers for their thorough evaluation of our manuscript and their constructive feedback, which has guided us to substantially revise and enhance this study. We have carefully addressed each comment and made major methodological and interpretative improvements throughout the manuscript. All changes are tracked in the revised Word version of the manuscript. Please note that all line and page numbers referred to in this response document correspond to the unmarked PDF file generated by the submission system. The key revisions are summarized below:

- **Statistical test:** We have replaced all previous statistical analyses with a non-parametric framework using the Mann-Whitney U test, complemented by effect size assessment with Cliff's delta. Importantly, all major conclusions remain unchanged under these revised statistical criteria.
- **Expanded benchmarking:** We incorporated additional benchmarking analyses, including (1) *de novo* gene descriptions generated through a retrieval-augmented generation (RAG) approach based on up-to-date biomedical literature, (2) inclusion of a non-LLM, word-level embedding model (BioWordVec) as a baseline, and (3) integration of disease annotations from the GWAS Catalog to extend evaluation to disease-associated gene sets.
- **Comparison with existing gene set-based methods:** We clarify the conceptual and technical advances of our framework relative to traditional gene set-based analyses. Through real-world applications, we demonstrate how our approach enables functional hypothesis testing in contexts where predefined gene set annotations are incomplete or unavailable: (1) the systematic identification of drug mechanisms of action from differential gene expression following drug treatment, and (2) statistically evaluating *de novo* functions inferred by emerging LLM tools (*e.g.*, Hu *et al*. *Nature Methods*, 2025; PMID: 39609565), where LLM-generated confidence scores are qualitative and lack statistical grounding.
- **Novel biological insights:** The revised manuscript highlights new biological findings, including previously unannotated gene clusters that govern essential cellular processes. In the case studies, we reveal an emerging immunologic pathway involved in immunotherapy sensitization and novel gene-mechanism associations relevant to drug repurposing. Both findings are independent of the data used to train the embedding model and post-date the underlying gene annotations, remaining consistent with but unreported in current literature and representing promising directions for future experimental validation.
- **Additional improvements:** We have optimized the number of clusters (k) in the k-means analysis, enhanced the named entity recognition pipeline for interpreting embedding-derived gene clusters, and developed an easy-to-use R package (DEGEmbedR) for embedding-based annotation of differentially expressed genes to promote reproducibility and community adoption.

We hope that these substantial revisions address the reviewers' concerns and significantly strengthen the manuscript. We greatly appreciate the reviewers' time and thoughtful comments, which have helped us refine both the rigor and the impact of this work.

Reviewer #1 (Remarks to the Author):

In this work authors evaluate embeddings of genes and biological functions derived from state-of-the-art LLM models to assess their biological relevance, specificity, and interpretability. The benchmark evaluates their ability to capture gene-gene functional similarities and gene-function relationships across diverse biological contexts.

Authors state that this study demonstrates the potential of LLM-derived embeddings to enhance quantitative functional representation and facilitate hypothesis testing of gene functions towards improving the reliability and practical applicability of LLMs in functional genomics and computational biology.

The framework presented adds a critical statistical layer for evaluating LLM-inferred gene functions, enabling hypothesis testing without predefined gene sets and enhancing the reliability of LLM-guided functional discovery.

This study presents the first systematic and statistical evaluation of LLM-based embedding models for gene function representation. It a very valuable work, with an important amount of experimetal work involved. The study is original, very complete and comprehensive, and it is certainly of interest to the community.

Response: We thank the reviewer for the positive feedback and thoughtful comments. We have revised the manuscript to further improve its clarity and rigor, and we hope the changes satisfactorily address all concerns.

I have just some minor comments in order to further improve the work:

- The data selected for the experiments is quite reduced, considering that there are millions of proteins in UniProt. Why just choosing less than 20,000 genes? The data selection should be better justified.

Response: We appreciate the reviewer's comment. Our analysis includes all human protein-coding genes available across three major databases: NCBI Gene, Alliance of Genome Resources, and UniProt. We focused on these genes because they represent the best-annotated portion of the human genome with reliable functional summaries, and are typically the primary focus of functional annotation analyses. We have clarified this rationale in the Methods section (lines 491-493; pages 23-24 in the unmarked PDF file).

- I do not understand the objective of the data ablation in Fig 2d. What should be expected form such experiment? that the removal of a term should impact significantly in the embedding? if that is the case a 5% reduction is not really a big reduction, isn't it? On the other hand, one could also expect that a good embedding should not be so sentitive to a word removal. This experiment should be better explained.

Response: We thank the reviewer for this insightful question. We would like to clarify that the ablation analysis was designed to enhance the interpretability of the "black-box" LLM embedding models by quantifying the relative contribution of key biological terms to the captured semantic meaning, rather than to test model sensitivity. In the revised manuscript, we have refined this analysis to evaluate the semantic cohesion and interpretability of each gene cluster. For the illustrative example of the cluster annotated as "RNA-associated protein binding regulation," removal of key named entities such as "RNA binding activity" and "protein binding activity" across all genes in the cluster resulted in a 15.7% and 13.1% reduction, respectively, in mean gene-gene cosine similarity. This analysis helps identify the biological terms that most strongly influence the organization and coherence of each functional cluster, thereby facilitating interpretation. We have revised the corresponding Results and Discussion sections (lines 149-157, page 8; lines 373-375, page 18) to clarify the purpose and interpretation of this analysis.

- Why ablation was not performed with all LLM methods evaluated?

Response: We focused the ablation analysis on OpenAI-TE3, as recent studies have reported the superior performance of OpenAI LLMs and embeddings in functional genomics (Refs. 5-7 in the manuscript). Consistent with these reports, our benchmarking results later in this manuscript also demonstrate that OpenAI-TE3 achieved the best overall performance in capturing gene functions. We note that because named entity recognition is context dependent, performing ablation across all seven LLMs would require independent entity extraction and regeneration of all gene embeddings for each ablated term, which would be computationally intensive and costly, particularly for proprietary embedding models. Therefore, this was not pursued in the present study. We have clarified the rationale for model selection in the corresponding Results section (lines 97-99, page 6).

- Why only k-means for clustering and why k=100? I think this should be better justified. Or even better, several clustering algorithms could be tried and results compared in order to see if conclusions still hold.

Response: We thank the reviewer for this valuable suggestion. We have now employed an enhanced elbow method to identify the optimal inflection point in the sum of squared errors curve (tested between $k = 15$ and 300), which determined the optimal k value of 103. This setting also produced cluster sizes ranging from 36 to 406 genes, which fall within an ideal range for subsequent analysis and interpretation. The details have been added to the Methods section (lines 516-522, page 25). As the clustering was primarily used to visualize and interpret overall patterns and to aid readers' understanding of the methodology, a comprehensive comparison across clustering algorithms was considered beyond the scope of this study.

Reviewer #2 (Remarks to the Author):

Summary:
This paper recognized a limitation of traditional functional annotation, which relies on human-curated and static databases that limit the opportunity to capture novelty and dynamics of gene functions. To address this, the authors developed a framework that uses LLM-generated embeddings from gene function descriptions and quantitatively evaluated the gene-gene and gene-function relationships derived from the embeddings for biological relevance. Their evaluation approach involves 1) an ablation study to find the key word for gene function alignment, 2) the systematic evaluation of sensitivity and specificity for the functional representation from the embedding space, 3) hypothesis testing for real-world gene sets not previously documented, 4) statistical testing for gene function hypotheses, overcoming the lack of significance scoring in an existing LLM-based gene set functional annotation tool. The key findings claimed by the paper include that the text embeddings achieved high sensitivity and specificity for predefined gene functions. Their framework is heavily dependent on statistical analysis, but the authors did not justify the use of statistical methods, and the significant scores may be inflated due to unmet assumptions. Additionally, the main impact and real-world applications of hypothesis testing are not clearly outlined, and the embedding approach relies on predefined gene function descriptions from curated databases. This dependency leaves it unclear how the approach addresses the stated limitation of over-reliance on static resources. The traditional functional enrichment approach can already provide statistical results and return terms ranked by the significance of overlap between gene lists. In contrast, the statistical score proposed by this study seems less intuitively interpretable, as it depends on the significance of the separation between inferred distributions.

Response: We greatly appreciate the reviewer's thorough evaluation of our manuscript and their constructive suggestions. As detailed below, we have carefully revised the manuscript to address all comments by incorporating an updated statistical framework, clarifications regarding the conceptual and technical novelty of our framework, new analyses and discussions, and additional literature support. We believe these revisions have substantially improved the quality and clarity of the manuscript, and we hope that they satisfactorily address the reviewer's concerns.

Concerns:
The abstract needs to highlight the key findings from the paper, whereas it currently reads more like a description of the methods than a summary of the findings. The impact and practicality are also not highlighted in the abstract.

Response: We thank the reviewer for this helpful suggestion and have revised the abstract to better highlight the key findings, impact, and practical significance of the study.

There is a significant gap between motivation and implementation. The authors point out that the limitation of functional annotation is that it depends on databases that are manually curated and static. However, their LLM-based approach embeds pre-defined gene function descriptions from static databases such as NCBI, making it unclear how their approach addresses this issue. Furthermore, functional enrichment can be performed using gene sets annotated from experimental

data, such as GWAS catalogs or gene expression signatures from transcriptomics data, which can capture some context-specificity. However, the paper did not consider how the embedding approach could adapt to this.

Both traditional functional enrichment analysis and this LLM embedding approach provide metrics of significance, but how do the significance and top enriched functional terms differ between the two methods? Do the methods yield substantively different results that would affect the insight gained by a researcher exploring a gene set? Again, given that the author's approach also relies on curated gene function descriptions in a canonical database, the gain of information using this approach is not clear. Since it is faster and cheaper to use functional enrichment, what are the benefits of using LLM embeddings?

Response: We thank the reviewer for raising these important points and have combined these related comments to provide a comprehensive response. Our framework represents both a conceptual and technical advance over traditional gene set-based annotation methods. By generating functional embeddings directly from biological function descriptions, our approach does not require predefined gene sets for analysis. This distinction is significant because it enables functional hypothesis testing in contexts where curated gene set annotations are incomplete, unavailable, or static, which is a limitation that arises from the labor-intensive nature of manual curation. We demonstrate the utility of our framework in both hypothesis-driven and hypothesis-free contexts:

- In the hypothesis-driven scenario of drug mechanism studies, our approach enables the systematic identification of mechanisms of action (MOAs) from differential gene expression following drug treatment, which is an area where, to our knowledge, no comprehensive gene set databases currently exist (as discussed in later responses).
- In the hypothesis-free scenario, our framework provides a critical statistical layer for emerging LLM-based functional inference models (*e.g.*, Hu *et al*., *Nature Methods*, 2025; PMID: 39609565) that generate *de novo* functional annotations from a given gene list with descriptive, text-based "confidence scores" that lack statistical grounding. Because such LLM-derived annotations are *de novo*, curating corresponding gene sets for significance testing is impractical, which is a critical gap directly addressed by our framework. As LLMs continue to evolve and drive increasingly ambitious biomedical applications, our embedding-based method adds an essential layer for ensuring statistical rigor and interpretability.

We have expanded the Introduction and Discussion sections (lines 51-57, page 4; lines 378-396, pages 18-19; lines 464-469, page 22 in the unmarked PDF file) to explicitly discuss this conceptual novelty and its implications for advancing LLM-based functional genomics.

To promote broader accessibility, we have developed an open-source R package, DEGEmbedR, which enables embedding-based annotation analysis of differentially expressed genes across multiple biological knowledge bases, including Gene Ontology Biological Process (GO-BP), Canonical Pathways (CP), and MOA categories. The package also supports the generation and evaluation of customized functional descriptions for user-defined hypotheses.

Finally, in response to the reviewer's suggestion to incorporate additional functional annotations, we have expanded our benchmarking to include four annotation datasets, grouped into two major classes:

- Focused annotations, including GO-BP and CP collections from MSigDB, which represent well-defined and specific biological functions; and
- Diffuse annotations, including Chemical and Genetic Perturbation (CGP) signatures from MSigDB and GWAS-based disease associations from the GWAS Catalog, which are typically multifactorial and context dependent, as the reviewer noted, reflecting the complexity of perturbation responses and disease processes.

These annotation datasets are now incorporated across all evaluation tasks. We have expanded the rationale and discussion of these databases and their comparative performance in the Results (lines 162-170, pages 8-9) and Discussion (lines 422-429, page 20) sections.

Basing the method on the embedding of curated gene function descriptions makes it heavily dependent on common genes that are well-studied and well-documented. This mirrors the structure of the curated gene function databases. The author's methods have limitations in discovering new gene/protein functions, especially for understudied proteins. Those are more impactful challenges to overcome in biology and would be of greater interest. Can the authors comment and address this limitation?

Response: We appreciate the reviewer's thoughtful comment on this important limitation and the potential for extending our framework to uncover novel or understudied gene functions. In the revised manuscript, we have introduced retrieval-augmented generation (RAG) to dynamically generate *de novo* gene function summaries based on the retrieval of up-to-date literature from PubMed. Our results indicate that embeddings derived from NCBI Gene descriptions continue to provide the strongest overall representation compared with RAG and two widely used gene databases (UniProt and Alliance of Genome Resources) (lines 110-119, pages 6-7), likely due to the comprehensiveness of the NCBI database. Nonetheless, we acknowledge the future potential of the RAG approach, particularly when combined with domain-specific retrieval pipelines, to better capture context-dependent biology such as cell type- or disease-specific functions (lines 430-445, pages 20-21). This hybrid framework increases coverage for underexplored genes and supports the discovery of novel pathways, as illustrated by the emerging STING/cGAS mechanism identified in this study (case study discussed in a later response).

The authors grouped gene function embeddings into 100 clusters and then annotated them using LLM. How do those clusters compare with existing gene clusters from known databases? If there are differences, are there new findings? (i.e., new genes being clustered in a well-studied gene set group or a new functional term). It would strengthen the paper if they compared cluster composition to known biological clusters.

Response: We thank the reviewer for this insightful suggestion, which indeed provides a valuable way to examine novel biology captured by the LLM embeddings. We have now optimized the number of clusters using an enhanced elbow method, which determined an optimal *k* value of 103

(cluster size, 36-406). Among these 103 clusters, seven clusters (6.8%) showed no significant overlap with any Gene Ontology (GO) term across all three categories, including biological processes, molecular functions, and cellular components. These clusters were associated with diverse biological themes, including gene regulation and expression, cellular organization and differentiation, and protein or extracellular interactions. For example, the cluster annotated as "RNA-associated protein binding regulation" unites RNA-binding proteins (*e.g.*, *RBM33*, *RSRC2*, *R3HDM1*) involved in RNA processing, modification, and export with protein-binding adaptors (*e.g.*, *SH3GLB2*, *UBFD1*, *PHLDB3*) that organize ubiquitin- and membrane-associated complexes. Together, these genes form a coordinated module regulating RNA-protein interaction networks that underpin post-transcriptional control but are not represented in current GO hierarchies. This example illustrates how LLM-derived embeddings can uncover semantically unified yet previously unannotated functional relationships beyond classical ontology definitions. The updated results are presented in the Results section (lines 131-157, pages 7-8; Fig. 2b and 2d and Supplementary Table 3).

The authors did not seem to review the keywords used for the clustering, as there are multiple obvious errors: 1) keywords are dominated by non-biological terms like "predicted" and "located" and very general biological terms like transcript, variant, and encoding, 2) they include semantic duplicates such as "olfactory" vs. "Olfactory", "enzyme" vs. "enzymes", and 3) biological terms were separated into individual words, such as "RNA Polymerase II" → "RNA", "Polymerase", "II". The authors should consider the method that extracts the biological terms from the functional description instead of splitting the word.

Response: We agree with the reviewer and have updated our named entity recognition pipeline accordingly. The revised pipeline now retains multi-word biological terms as single entities and applies lemmatization to unify related forms. The associated results have been updated in Fig. 2b, Fig. 2c, Supplementary Fig. 2, and Supplementary Table 3. We have confirmed that "RNA Polymerase II," identified in clusters 35, 52, 78, and 81, is now correctly recognized as a single term (Supplementary Table 3). We note that cluster 35, which primarily focuses on RNA polymerase II functions, includes not only "RNA Polymerase II" but also derivative terms such as "RNA polymerase II cis-regulatory region sequence-specific DNA binding," which are meaningful because they correspond to specific GO terms (e.g., GO:0000978) present in the biomedical vocabulary of the entity recognition model we used (*en_core_sci_lg* from the Python package *scispaCy*).

What is the motivation for the analysis of inter-model similarity in gene clusters? There is no clear conclusion for the comparison between different embedding models.

Response: We agree with the reviewer's concern and have therefore removed this analysis and the corresponding figure from Fig. 2.

There are substantial concerns about the statistical methods the authors chose to calculate the significance. Take the analysis for the gene-gene similarity as an example: Is the size of inter-

function similarities the same as intra-function similarities? The one-tailed t-tests asked the question whether the mean similarity of the intra-function group is larger than the inter-function group. However, n number of genes can result in n*(n-1)/2 intra-function pairs that are not completely independent observations and may therefore lead to very significant results with small mean differences. The authors did not justify the assumption for choosing to use t-test over other non-parametric tests, such as the Wilcoxon rank-sum test, which does not assume independence. The effect size (the difference between means) should be taken into consideration when determining significance (for example, $p < 0.05$ and mean difference > X). Similar statistical analysis concerns applied to all related tasks described in the paper.

Response: We fully appreciate the reviewer's insightful critique regarding the statistical framework. In response, we have revised all statistical analyses throughout the study to employ the nonparametric Mann-Whitney U test. To ensure that statistical significance reflects biologically meaningful effects, we now complement all tests with Cliff's delta as a nonparametric effect-size measure. Statistical significance is defined as a one-tailed Mann-Whitney U test with $p < 0.05$ and Cliff's $\delta \geq 0.147$ (a non-negligible effect, equivalent to Cohen's $d \geq 0.2$, often used to represent small effect). Importantly, under these revised and more stringent criteria, all major conclusions across both evaluation and application tasks remain unchanged.

We have also expanded the Discussion section (lines 446-457, page 21) to justify our statistical approach and address the reviewer's specific concerns.

The hypothesis testing task described in the paper is trivial and only confirmatory. For example, the STING pathway is already predefined as a gene set of interest to explore, and the test is to check whether related genes are enriched in the visugromab-treated DEGs. There is a gap in the logic: how is the STING pathway being recognized without knowing genes that are associated with it, and if the hypothesis itself is already based on that gene list, why is this hypothesis testing task needed? Can the embedding method be used to explore new/alternative pathways or mechanisms for the given observation? How would the authors expect researchers to use this method for real-world applications without prior knowledge of the STING pathway? What is the unique information from this approach that can help refine potential follow-up studies for the STING pathway and Visugromab mechanism, other than some p-value?

Response: We thank the reviewer for raising this important point and agree that additional clarification of the rationale and interpretation of this case study is needed. We have now revised the text to better explain the purpose and logic of the STING and visugromab analysis.

We selected two case studies, visugromab/STING and vortioxetine repurposing (the latter discussed in the next response), from papers published between September and December 2024. Both papers and datasets were released after the versions of the embedding model (OpenAI-TE3, released in January 2024) and gene summaries (NCBI Gene, accessed in February 2024) used in this study. This design minimizes the possibility of model-to-data leakage and demonstrates the framework's ability to generate novel biological hypotheses from embeddings derived from earlier data, particularly given the limited feasibility of wet-lab validation within this study.

We note that the hypothesis that "STING mediates visugromab-induced cancer immune sensitization" was not proposed in the original paper (Melero *et al*. *Nature*, 2025; PMID: 39663448) and remains novel to date. As elaborated in the revised Results section (lines 274-284, pages 13-14), the formulation of this hypothesis follows a rational, stepwise reasoning process that reflects how researchers might develop hypotheses in practice. Specifically, the original study showed that visugromab restores responsiveness to PD-1/PD-L1 therapy, suggesting that *GDF15* may contribute to immune suppression. A recent independent study in Parkinson's disease reported that *GDF15* suppresses the STING pathway (Wang *et al. Molecular and Cellular Biochemistry*, 2025; PMID: 40178669), which is an increasingly recognized key mediator of innate immune activation. Taken together, these observations led us to hypothesize that *GDF15* blockade by visugromab could enhance tumor immunity through STING signaling, a connection that has not yet been described in the existing literature.

Because the STING pathway is an emerging area of research and was not represented in the MSigDB version used in this study (2023.2.Hs), it was therefore untestable using existing gene set-based methods. To emulate researchers' prior knowledge, we generated a description of the STING pathway using GPT-4o (which also predates the study; knowledge cutoff: October 1, 2023; Supplementary Table 7). We then evaluated its embedding similarity with the visugromab-induced differential gene expression profile. The analysis revealed a significant correlation, providing computational support for the hypothesized mechanism. Notably, during the revision of this manuscript, a preprint released in October 2025 indirectly supported our hypothesis by reporting inverse expression changes between *Tmem173* (encoding STING) and *Gdf15* in murine cancer models (Chelette *et al.* bioRxiv, 2025.10.02.680158).

Our gene interpretation analysis, based on cosine similarities with the STING pathway embedding, identified *CXCL10* and *CXCL9*, which are canonical STING targets involved in T-cell recruitment, as showing particularly high similarities (Fig. 6c). These results suggest a potential mechanistic link between visugromab and STING activation in the context of cancer immunotherapy, which warrants further experimental validation.

To enable researchers to apply this framework to their own data and hypotheses, the accompanying R package (DEGEmbedR) integrates GPT-4o-based generation of functional descriptions for user-specified pathways or biological processes, conversion to embeddings, and statistical testing against user-provided differentially expressed genes. The package also outputs the top differentially expressed genes most correlated with each function to aid biological interpretation.

For the MOA hypotheses, how many MOAs were identified as significant in the study? According to Figure 6c, all the MOAs tested in the study are significant (i.e., even the last in the figure is highly significant). This raises concerns about whether the embedding method can discriminate between more or less relevant mechanisms. In addition, there is no gene set information reported that leads to the significance of the MOA ranking. It is unclear whether this approach is to differentiate the specific associations or just generic signals. And, beyond that, the purpose of this step is also unclear. What information is added, given that the calcium signaling is already identified in the drug-target network.

Response: In the second case study, we focused on identifying the MOAs underlying the repurposing potential of vortioxetine in glioblastoma. As discussed in the revised Discussion section (lines 386-401, pages 18-19), to the best of our knowledge, there are no comprehensive MOA gene sets that enable systematic identification of MOAs from differential gene expression data. Therefore, the original paper (Lee *et al*. *Nature Medicine*, 2024; PMID: 39304781) formulated its calcium-related hypothesis through manual interpretation of a gene-target network highlighting AP-1 transcription factors and immediate early genes (IEGs), which were then linked to calcium signaling based on literature evidence, as quoted: "*AP-1 activation and IEG expression are typically preceded by Ca2+-dependent signaling in neural lineage cells44,50,52,53. We, therefore, measured both extracellular Ca2+ influx …*"

In contrast, our study implemented a systematic analysis using embeddings generated from textual descriptions of 522 common MOAs, *without involving any predefined gene sets* (as illustrated in the newly added Fig. 6a). This analysis identified calcium channel regulation as the top MOA associated with the differentially expressed genes of vortioxetine, along with eight additional significant MOAs under the updated statistical criteria (revised Fig. 6d). Importantly, several of these high-confidence MOAs, such as such as those involving calcitonin, corticotropin-releasing factor (CRF), estrogen, and bradykinin, were not highlighted in the original study, yet each has been independently implicated in brain tumor biology and therapeutic response.

Furthermore, our network analysis of significant MOAs and their top correlated differentially expressed genes revealed three major signaling clusters corresponding to calcium/ion channel regulation, bradykinin-estrogenic modulation, and CRF-CRTH receptor signaling (Fig. 6e). These clusters represent a coherent mechanistic organization among calcium and neuroendocrine pathways and highlight potential hub genes governing vortioxetine's repurposing activity. We have substantially expanded the Results (lines 304-319, page 15) and Discussion sections (lines 402-421, pages 19-20) to include a more detailed discussion and additional literature support for these findings.

In summary, across the two case studies, our results demonstrate that embedding-based MOA analysis not only recapitulates known pharmacological effects but also uncovers previously underappreciated, interconnected signaling modules and specific gene-mechanism associations. This approach provides a scalable and hypothesis-driven framework for drug mechanism discovery and repurposing.

For the comparison with GSAI's confidence score, is the 0 confidence being considered in the distribution? Zeros were reported from the original paper, but not shown in the Supplementary Fig. 6. Are there no zeros being returned, or are the authors excluding them? If excluding, please provide the rationale.

Response: We thank the reviewer for this helpful comment and confirm that none of the GSAI inferences returned a confidence score of 0. To avoid confusion, we have now displayed the full possible range of confidence scores (0 to 1) in Supplementary Fig. 8.

Reviewer #2 (Remarks on code availability):

Given the concerns that we raised, it did not seem meaningful to review the code.

Response: We appreciate the reviewer's note regarding code availability. To ensure full transparency and reproducibility, as detailed in the Data Availability section, we now provide both the complete analysis code for reproducing all major results and an open-source R package (DEGEmbedR) that enables users to easily apply our framework for annotating differentially expressed genes. The code and package will be made publicly available and include detailed documentation and example workflows to facilitate independent verification and reuse by the research community.

Reviewer #3 (Remarks to the Author):

This manuscript presents a computational framework to evaluate and apply large language model (LLM)-derived embeddings for gene function representation and hypothesis testing. The authors benchmark multiple LLMs using gene- and function-level annotations from public databases and demonstrate two applications related to drug mechanism discovery. The manuscript explores a timely and promising application of large language models (LLMs) in gene function representation. However, the technical depth and conceptual advancement remain limited.

Response: We thank the reviewer for their constructive feedback and recognition of the potential of our work. In the revised manuscript, we have strengthened both the technical depth and conceptual clarity of the study. We highlight several key advances below:

- **Conceptual innovation:** Unlike traditional gene set-based analyses that rely on static annotations, our approach enables statistical hypothesis testing directly from functional embeddings generated from textual descriptions of biological functions. This allows efficient and scalable discovery in contexts where curated gene sets are incomplete or unavailable, as demonstrated in both hypothesis-driven and hypothesis-free applications described below.
- **Technical rigor:** Our updated nonparametric statistical framework enables *the first* comprehensive evaluation of seven state-of-the-art LLM embeddings and a non-LLM baseline model (BioWordVec) in representing gene-gene and gene-function relationships across four categories of functional annotations reflecting diverse biological contexts. This benchmarking establishes a strong foundation for real-world applications. We further improved embedding interpretability through an ablation analysis based on an enhanced named entity recognition pipeline and gene-function network analyses that reveal key mechanistic links in real-world case studies.
- **Applications to hypothesis testing:** We demonstrate how our approach supports functional hypothesis testing where predefined gene set annotations are incomplete or unavailable: 1) systematic identification of drug mechanisms of action from differential gene expression following drug treatment, and 2) statistical evaluation of *de novo* functional inferences from emerging LLM tools (*e.g.*, Hu *et al.*, *Nature Methods*, 2025; PMID: 39609565), where model-generated confidence scores lack statistical grounding.
- **Biological discovery:** Clustering of gene embeddings revealed novel gene clusters not captured by existing Gene Ontology annotations. Two real-world, hypothesis-driven drug mechanism studies, including visugromab/STING pathway activation and vortioxetine repurposing in glioblastoma, demonstrate the framework's ability to recapitulate known pharmacological effects while also uncovering interconnected signaling modules and specific gene-mechanism associations, providing a scalable and data-driven framework for hypothesis-driven drug mechanism discovery and repurposing.
- **Accessibility:** We now provide an easy-to-use R package (DEGEmbedR) that implements our framework for annotating differentially expressed genes and supports user-defined hypothesis testing.

Together, these revisions strengthen the study's contributions and demonstrate how LLM-derived embeddings advance functional genomics beyond traditional gene set-based analyses.

Major Concerns

1. Curated Summary Inputs Limits the Utility and Innovation of the LLM-based Framework

While the authors present a framework to compare gene and function embeddings across multiple LLMs, their exclusive use of curated summaries (e.g., from NCBI Gene) limits the linguistic richness and contextual diversity of the input. These structured, shallow texts do not fully leverage LLMs' strength in extracting relational and contextual knowledge from unstructured language. As a result, applying off-the-shelf LLM embeddings to such input offers limited advantage over traditional methods (e.g., Word2Vec, BioBERT) and weakens the motivation for using LLMs in this setting. The limited input prevents these models from showcasing their semantic capabilities, making cross-model comparisons less informative.

A more meaningful application of LLMs would be to analyze large-scale unstructured literature (e.g., PubMed abstracts or full texts), where their semantic reasoning capabilities could be more fully utilized. As it stands, the framework's reliance on curated summaries limits both its conceptual novelty and practical significance, falling short of the innovation threshold expected for publication in Nature Communications.

Response: We combined these two points to provide a more comprehensive response. We appreciate the reviewer's insightful comments and agree that the strength of LLMs lies in their ability to capture rich linguistic and contextual diversity. To address this, we have expanded our benchmarking to include both a comparison with a word-level embedding model and the use of less structured, LLM-generated gene and function descriptions.

First, to establish a non-LLM baseline for evaluating semantic representations, we incorporated BioWordVec, a state-of-the-art word-level embedding model trained on PubMed and clinical text corpora, across all evaluation tasks. Notably, in gene-gene similarity analysis, four leading LLM models, including OpenAI-TE3 and Gemini-TE4, outperformed BioWordVec (Fig. 3b). In gene-function analysis, BioWordVec trailed only OpenAI-TE3 and Gemini-TE4 in sensitivity (Fig. 4b and Supplementary Fig. 6) but exhibited lower specificity (ranked 6th of 8 models; Fig. 5b, Fig. 5e, and Supplementary Fig. 6), likely reflecting its emphasis on frequent co-occurrences of genes with functional and disease-related terms in biomedical texts. These results underscore the added semantic depth and contextual reasoning provided by LLMs in capturing biological relationships.

Second, we incorporated LLM-generated texts to better capture unstructured, context-specific, and potentially novel information beyond curated summaries:

- **Retrieval-augmented generation (RAG) of gene descriptions:** We introduced RAG to dynamically generate *de novo* gene function summaries based on up-to-date literature from PubMed. Our results indicate that embeddings derived from NCBI Gene descriptions continue to provide the strongest overall representation compared with RAG and two widely used gene databases (UniProt and Alliance of Genome Resources) (lines 110-119, pages 6-7 in the unmarked PDF file), likely due to the comprehensiveness of the NCBI database. Nonetheless, we acknowledge the future potential of the RAG approach, particularly when combined with domain-specific retrieval pipelines, to better capture context-dependent biology such as cell type- or disease-specific functions (lines 430-445, pages 20-21).

- ***De novo* generation of functional descriptions:** In the two drug cases studies, we used GPT-4o to automatically generate descriptions for hundreds of mechanisms of action (MOAs) and for the emerging STING pathway in cancer immunotherapy, demonstrating how LLMs can synthesize novel, biologically coherent function representations absent from curated databases (see Results section, "Hypothesis testing for drug mechanisms beyond predefined gene sets," starting on page 13).
- **Functional annotation inference using LLMs:** We integrated GSAI, which generates functional annotations for gene lists, and used our framework to provide statistical grounding for these emerging generative outputs (see Results section, "Statistical evaluation of LLM-generated *de novo* gene function hypotheses," starting on page 16).

Together, our framework now leverages the linguistic diversity and generative capabilities of LLMs to represent gene functions in a flexible, context-aware, and discovery-oriented manner.

Moreover, the curated summaries used as input often share substantial content overlap with benchmark annotation sources such as Gene Ontology (GO). This overlap introduces a risk of information leakage, where the inputs and evaluation metrics are not fully independent. As a result, it becomes difficult to assess the true semantic performance of the models, weakening the interpretability and rigor of the benchmarking process.

Response: We appreciate the reviewer's thoughtful comment and agree that potential information overlap between curated gene summaries and benchmark annotations warrants careful consideration. To address this, we assessed the gene clusters derived from NCBI Gene descriptions (Fig. 2b) and identified seven clusters (6.8% of 103 clusters) that did not overlap with any Gene Ontology (GO) terms across all three categories, revealing functionally coherent yet previously unannotated modules. These clusters were associated with diverse biological themes, including gene regulation and expression, cellular organization and differentiation, and protein or extracellular interactions. For example, the cluster annotated as "RNA-associated protein binding regulation" integrates RNA-binding proteins with protein-binding adaptors that coordinate post-transcriptional control. This example illustrates how LLM-derived embeddings can uncover semantically unified yet previously unannotated functional relationships beyond classical ontology definitions. The updated results are presented in the Results section (lines 131-157, pages 7-8; Fig. 2b and 2d).

Additionally, the comparable performance of canonical pathway (CP) annotations further supports this interpretation, as CP terms emphasize curated signaling and metabolic processes that are less likely to overlap with information in gene summaries. We have now included this discussion this in the Discussion section (lines 430-437, pages 20-21).

Furthermore, both drug case studies were intentionally designed to avoid data-to-model leakage, as the corresponding datasets and publications were released between September and December 2024, after the versions of the embedding model (OpenAI-TE3, released in January 2024) and gene summaries (NCBI Gene, accessed in February 2024) used in this study. Our analyses, which both recapitulated published findings and uncovered new, biologically meaningful results, demonstrate the framework's ability to generate novel hypotheses from embeddings derived from

earlier, independent data. We have expanded the Results and Discussion sections to clarify this rationale and emphasize the methodological safeguards ensuring that our benchmarking and applications reflect independent and biologically meaningful validation (lines 267-270, page 13; lines 396-401, page 19; lines 430-437, pages 20-21).

2. Limited accessibility and translational utility

The framework lacks a deployable implementation, such as a web-based interface or R/Python package, making it difficult for other researchers to apply the method in practice. This limits its utility and broader impact. I strongly encourage the authors to develop a user-accessible implementation to support adoption and reproducibility.

Response: We fully agree with the reviewer on the importance of accessibility to the broader research community. We have now developed and released an easy-to-use R package, DEGEmbedR, which implements our framework for annotating differentially expressed genes, as applied in the two case studies on drug mechanisms. As described in the Results (lines 325-328, page 16) and Data Availability sections, and detailed in the GitHub repository, the package enables embedding-based annotation of differentially expressed genes across Gene Ontology Biological Process (GO-BP), CP, and MOA categories. It also supports the generation and evaluation of customized functional descriptions, such as the STING pathway, and outputs the top differentially expressed genes most correlated with each function to aid biological interpretation.

The complete codebase for reproducing all major analyses in this study has also been provided to the reviewers and will be publicly released upon manuscript publication, as noted in the Data Availability and Code Availability sections.

Reviewer #4 (Remarks to the Author):

I co-reviewed this manuscript with one of the reviewers who provided the listed reports. This is part of the Nature Communications initiative to facilitate training in peer review and to provide appropriate recognition for Early Career Researchers who co-review manuscripts.

Response: We thank the Reviewer for co-reviewing the manuscript and providing their constructive feedback.

Reviewer #5 (Remarks to the Author):

I co-reviewed this manuscript with one of the reviewers who provided the listed reports. This is part of the Nature Communications initiative to facilitate training in peer review and to provide appropriate recognition for Early Career Researchers who co-review manuscripts.

Response: We thank the Reviewer for co-reviewing the manuscript and providing their constructive feedback.

Manuscript #: NCOMMS-25-46979B-Z
Revised Title: An embedding-based framework enables statistical testing of gene-set function hypotheses inferred by large language models

Overall Response: We sincerely thank all five reviewers for their careful re-evaluation of our manuscript and for their constructive and rigorous critiques. In this substantially revised version, we have fundamentally clarified the conceptual positioning of the study, strengthened systematic benchmarking against existing approaches, expanded the integration of retrieval-based LLM methods, and implemented additional safeguards addressing concerns of independence and information leakage. The manuscript title has been revised to more precisely capture the central contribution. All modifications are tracked in the revised Word file. Line and page numbers referenced in this document correspond to the unmarked PDF generated by the submission system. The major revisions are summarized below:

1. **Clarifying the core research gap and re-centering the conceptual advance.**
   Emerging gene-set-free LLM methods can generate *de novo* functional hypotheses directly from gene lists (*e.g.*, PMIDs: 39609565 and 40721871), yet they lack a principled statistical framework to test whether an inferred function is quantitatively supported by the input genes. This missing hypothesis-testing layer represents the central research gap addressed in our study. We have substantially revised the Abstract, Introduction, and Discussion to explicitly articulate this gap and to position our work as a statistical validation framework rather than an alternative annotation method.
   To logically establish this advance, the Results are now reorganized into a clear progression (updated Fig. 1): (i) benchmarking whether embeddings encode biologically meaningful gene-gene and gene-function structure using controlled ontology and pathway references; (ii) demonstrating robustness under systematic contamination to quantify discrimination between biological signal and noise; and (iii) applying the framework to statistically evaluate *de novo* LLM-inferred gene-set function hypotheses in real-world experimental gene systems.

2. **Demonstrating advancement over traditional enrichment and existing LLM methods.**
   In response to Reviewer #2, we now provide systematic, quantitative comparisons between our embedding-based hypothesis testing framework, conventional gene set overrepresentation analysis, and LLM-generated confidence scores. For LLM-inferred functional hypotheses, we implement enrichment-based testing and explicitly characterize differences in prioritization, discrimination, and mechanistic resolution. We further incorporate negative controls through systematic contamination analyses, systematic evaluation of experimentally derived gene lists, and two case studies from these gene lists to demonstrate that the framework differentiates competing hypotheses rather than merely confirming plausible associations. Collectively, these analyses establish that our method adds a statistically rigorous validation layer to LLM-generated functional inference and provides discriminatory power beyond gene-overlap-based enrichment and heuristic LLM confidence scores.

3. **Fully integrating retrieval-based and generative LLM capabilities.**
   To directly address concerns regarding reliance on curated summaries, we now

systematically incorporate retrieval-augmented gene descriptions across all benchmarking and application tasks throughout the study, with primary analyses centered on retrieval-based representations. We implement context-dependent retrieval strategies to generate function-, pathway-, perturbation-, and disease-specific gene descriptions from the literature, enabling dynamic and context-aware representation. These retrieval-based embeddings are evaluated alongside curated, static summaries and demonstrate comparable or superior performance, particularly in diffuse and context-dependent functional categories. Together, these results show that the framework substantively leverages LLM generative capacity rather than merely reflecting curated annotation structure.

4. **Strengthening safeguards against information leakage.**
   Retrieval-based gene descriptions were constructed independently from ontology annotations, thereby conceptually reducing lexical and structural overlap between input representations and evaluation benchmarks. In addition, we implemented temporal hold-out evaluations using GO and pathway terms added after the literature cutoff used for gene description generation. Together, these analyses address independence concerns and clarify that performance reflects semantic generalization rather than annotation redundancy.

5. **Updating embedding models for technical currency.**
   As text-embedding-004 (Gemini-TE4) was deprecated in January 2026, we replaced it with Google's latest and recommended model, gemini-embedding-001 (Gemini-E1), and reran all relevant analyses to ensure reproducibility and alignment with current best practices.

We believe these revisions directly address the reviewers' central concerns and substantially strengthen both the conceptual clarity and empirical rigor of the manuscript. We are grateful for the reviewers' guidance, which has materially improved the focus and impact of this work.

Reviewer #1 (Remarks to the Author):

Authors have satisfactorily responded to all my comments. Thanks.

Response: We thank the reviewer for the positive feedback.

Reviewer #2 (Remarks to the Author):

Comments:

We appreciate the authors for making updates to the keyword extraction; we agree that the resulting keywords are now better suited for clustering. Thanks to the authors for their efforts in revising the analyses to use non-parametric statistical tests. The study now presents a good systematic evaluation framework for future similar gene/ function-embedding tasks.

Response: We thank the reviewer for reviewing our manuscript again and for the positive feedback.

Our greatest concern remains:

The authors have not adequately addressed our comments regarding the intended uses of the method and the need for benchmarking versus gene set enrichment, the dominant method in the field. There seems to be a misunderstanding of our concerns.

Response: We thank the reviewer for clarifying this central concern. In this revision, we have directly addressed the intended use of the framework as the critical but currently missing statistical validation layer for LLM-inferred *de novo* functional hypotheses generated from gene lists (see Abstract, page 3; Introduction, lines 59-70 and 81-84 on pages 4-5; and Discussion, lines 411-431 on page 21).

We have also implemented systematic benchmarking against conventional gene set enrichment analysis, including quantitative comparisons and negative controls, to explicitly characterize how and when our method provides distinct advantages (see below).

The two examples of hypothesis testing are anecdotal rather than systematic and do not compare the results to an enrichment approach, see below. There is also a lack of systematic quantitative comparison of how results differ from those of traditional enrichment analysis over a test corpus. The critical point is to provide evidence characterizing how the technique yields different results across a broad range of data. Furthermore, the insights provided to a researcher by their method should be clearly compared with those from enrichment analysis.

In the STING and visugromab example, the authors first proposed the novel hypothesis that STING mediates visugromab-induced immune sensitization and that similarity between visugromab-induced genes and a GPT-4o-curated description of the STING pathway would support this hypothesis. But what is the difference between asking the LLM to generate a text description and asking it to curate a gene list? The authors could have asked GPT-4o to generate a 'curated' gene set for the STING pathway, add to the reference corpus, and run the enrichment analysis?

In the vortioxetine example, the authors first developed the novel hypothesis that vortioxetine acts via calcium channels in glioblastoma, then curated the MOA description embeddings using GPT-

4o. Similarly, the authors could have asked the LLM to curate the gene list related to the MOA and run the enrichment analysis. Then compare their approach with the enrichment analysis.

Note that the purpose of hypothesis testing is to falsify unlikely conditions. The author's current hypothesis testing scenarios lack negative controls. For example:

The authors did not compare the similarity of the STING description to comparable GPT-4o-generated descriptions of other immune pathways. GDF-15 may have multiple modes of action; can the STING pathway be meaningfully differentiated from alternatives? If the authors can show the DEG embeddings are much closer to STING than other pathways, the hypothesis is more likely to be true.

Their MOA analysis found calcium/ion channel regulation as the top "hit", but are there negative controls in the analysis? What MOAs should not be associated with vortioxetine? Are the distances of gene embeddings against negative controls larger than the expected positive controls? Please consider including these results in the analysis.

Response: We thank the reviewer for these important comments and have combined them into a comprehensive response. We agree that the **two drug case studies** were not sufficiently systematic as primary demonstrations. In this revision, we have substantially restructured the Results to replace them with a logically ordered and comprehensive evaluation framework. Specifically, we first establish gene-gene and gene-function performance using curated ontology and pathway annotations as controlled references. We then conduct systematic contamination analyses to quantify discrimination between biological signal and noise, which is critical for applications to real-world high-throughput datasets. Finally, we apply the framework to 50 experimentally-defined NeST (Nested Systems in Tumors) protein assembly gene sets, which have been used in recent LLM benchmarking studies (PMIDs: 39609565 and 40721871), to evaluate *de novo* LLM-inferred functional hypotheses in a standardized and experimentally grounded setting. Two representative NeST systems were subsequently examined to derive mechanistic insights and assess negative controls. This progression provides broad, quantitative validation across diverse datasets rather than relying on selected illustrative examples.

We appreciate the reviewer's suggestion to **benchmark against enrichment analysis**. Accordingly, we implemented gene set overrepresentation analysis (ORA) in conjunction with LLM functional inference. For each LLM-inferred functional hypothesis, we generated corresponding gene sets using GPT-4o and performed conventional ORA. We then systematically compared prioritization and ranking among our embedding-based statistical framework, ORA, and LLM-inferred confidence scores. Across 50 NeST systems, GO-BP rankings derived from our embedding-based framework showed substantially higher agreement with the semantic reference ranking than ORA (median Spearman correlation 0.43 vs. 0.18; one-tailed Wilcoxon signed-rank test $P = 1.2 \times 10^{-14}$; Fig. 7a). For *de novo* LLM-inferred hypotheses (36 NeST systems with sufficient predictions), embedding-based prioritization achieved significantly higher concordance with the reference ranking than both ORA ($P = 9.3 \times 10^{-3}$; Fig. 7b) and confidence scores ($P = 0.021$). Moreover, hypotheses classified as significant by our framework were significantly more aligned with the reference ranking than non-significant predictions ($P = 2.4 \times 10^{-4}$), whereas ORA did not effectively distinguish coherent hypotheses ($P = 0.085$). These results are detailed in the

revised Results section, "*Evaluation on LLM-inferred functions from real-world experimental gene lists*" (page 17; Fig. 7a-b).

Regarding **negative controls and falsification**, we agree that hypothesis testing must distinguish true biological signal from plausible alternatives. We therefore incorporated systematic contamination experiments, progressively diluting curated gene sets with random genes to evaluate robustness under controlled signal degradation. Although LLM-generated functions are inherently plausible by construction, our statistical framework showed a marked decline in significance with increasing contamination, whereas LLM confidence scores were substantially less responsive (Fig. 6b). In addition, examination of two representative NeST systems, one in which all LLM-inferred functions were biologically coherent and another containing both strongly supported and weakly supported hypotheses, demonstrates that our framework differentiates competing mechanistic hypotheses rather than uniformly supporting all plausible terms (Fig. 7c-d). These results are detailed in the revised Results sections "*Statistical evaluation of LLM-inferred de novo gene-set function hypotheses under contamination*" (page 15) and "*Representative case studies from NeST systems*" (page 18). Together, these analyses show that embedding-based statistical testing provides stronger discrimination between biologically meaningful hypotheses and noise than enrichment analysis or heuristic LLM confidence scores alone.

Notably, the promise of the method is that it can make use of non-curated information. But in the current paper, they focus on the use of curated information, similar to enrichment analysis, with RAG left as a "promising approach".

Response: We thank the reviewer for this insightful comment. In this revision, we have fully integrated RAG as the primary source of gene descriptions rather than treating it as a secondary or future extension. Specifically, we generated four context-aware RAG descriptions per gene using relevant sentences retrieved from PubMed, capturing functional roles, pathway associations, perturbation responses, and disease relevance of the gene. This flexible and context-aware strategy enables dynamic, literature-grounded representations that extend beyond static curated summaries. As shown in Fig. 2a and Supplementary Fig. 1, RAG-derived embeddings exhibit strong concordance with curated NCBI descriptions at the same-gene level, indicating comparable semantic grounding. Across gene-gene and gene-function benchmarking analyses, RAG-based embeddings performed superior or comparably to curated NCBI descriptions while offering greater contextual flexibility. Accordingly, the primary analyses presented in Figs. 2-7 are based exclusively on RAG-derived embeddings, while results based on NCBI descriptions are provided in Supplementary Figs. 1 and 6 for comparison. Together, these revisions demonstrate that the framework substantively leverages literature-based information rather than relying primarily on curated databases.

Finally, in the discussion, pg. 19 line 396-397: "Notably, our framework produced promising findings such as the visugromab-STING link and alternative signaling of vortioxetine…" is too strong without addressing the comments raised above. It reads like this study developed and validated the hypotheses, but the authors developed novel hypotheses outside their framework.

Response: We thank the reviewer for this comment. Both drug case studies have been removed from the revised manuscript to avoid reliance on non-systematic examples and potential overstatement.

Other issues:

Thanks to the authors for updating the abstract, including more details of key findings and an overview of the study. The first two sentences, however, do not yet identify the key gap and how LLMs can help.

Response: We thank the reviewer for this helpful suggestion. We have revised the opening sentences of the Abstract to clearly define the key research gap and to explicitly explain how this study demonstrates that LLM-derived embeddings can address this limitation.

Fig. 2a needs a legend divider to explicitly show that the top three are the same genes across databases and the bottom three are different genes in the same database.

Response: We thank the reviewer for this helpful suggestion and have revised Fig. 2a to include a clear legend divider explicitly distinguishing same-gene comparisons across databases from different-gene comparisons within the same database.

Page 7 line 123-125 authors stated "Functionally related clusters emerged in close proximity…", but there is no data or result showing the distances between clusters. The t-SNE analysis only preserves local relationships, not the global distances, so this statement was not supported. Suggest providing supporting evidence or removing the claim in the result.

Response: We have revised the Results text to remove the unsupported claim and clarified in the Fig. 2 legend that t-SNE preserves local structure but not global inter-cluster distances, to avoid misinterpretation.

Intra-cluster gene-gene cosine similarity was first mentioned in line 139 on pg.7. Without context, it is hard to interpret what 0.75 means in relation to other clusters (i.e., does that mean it is a really tight cluster or around medium). Suggest providing a distribution plot of all intra-cluster cosine similarities to support the number; otherwise, this number here solely does not provide much information to understand the cluster (it can be moved to the ablation analysis paragraph as a parentheses, i.e., dropped X % from 0.75 to X).

Response: Following this helpful suggestion, we have added Supplementary Fig. 2 showing the distribution of intra-cluster gene-gene cosine similarities across all clusters to provide context for the reported values. In addition, we revised the main text to report ablation-associated changes as percentage differences (Fig. 2d) to improve interpretability and comparability across clusters.

Line 219-220 on pg. 11 said the CGP description has limited functional coherence as it emphasized perturbation and experimental conditions. Is it really a limit? It is important to identify genes that behave similarly under certain conditions, as context matters. The result should be addressed differently.

Response: We thank the reviewer for this thoughtful comment. We have revised the corresponding text in the main manuscript to clarify that the lower coherence observed for CGP sets reflects their inherently context-dependent and multifactorial nature rather than a limitation of the embedding framework (Results: lines 199-209, page 11; Discussion: lines 456-466, page 23). We now emphasize that perturbation-driven signatures capture condition-specific biological responses and highlight the need for flexible, context-aware representations, which motivates and supports the use of our RAG-based framework for dynamically modeling such functions.

Fig. 4d is not referenced in the text.

Response: We thank the reviewer for the careful review and have now referenced this figure (now labeled as Fig. 4c) in the main text.

Line 301 on pg.15: Are there any citations that support the potential new MOA? Something in a different context? Skeptical if it is a purely new concept proposed without any external evidence.

Response: The drug case studies and associated MOA analysis have been removed from the revised manuscript.

Thanks to the authors for making changes and providing the full range of scores. The statistical score reported in the manuscript is promising. However, when I tested GSAI using the same model (gpt-4o-2024-08-06) on 100 random gene sets, it returned 94 zero scores. Therefore, a nontrivial number of zeros is expected in the score distribution. To help interpret and reproduce these results, the authors should consider rerunning the analysis and double-checking the result, or provide the data and code to enable us to reproduce the analysis.

Response: We thank the reviewer for this careful evaluation. We adapted the recommended GPT-4o-based GSAI prompt for "Biological Process Analysis" (https://idekerlab.ucsd.edu/gsai/), rather than "Process Naming and Analysis." The former takes an input gene list and generates individual biological process names, each accompanied by an LLM-assigned confidence score and a narrative explanation highlighting genes or proteins considered most relevant to the inferred process. These outputs provide both functional annotation and supporting rationale, which align directly with our goal of statistically evaluating *de novo* gene-set function hypotheses generated from input gene lists. In contrast, "Process Naming and Analysis" focuses more on identifying groups of proteins and emphasizing their interplay, including synergistic or antagonistic relationships and their integration within the broader system.

Our implementation retains the numeric confidence scoring described in the original publication and standardizes the output format to enable quantitative comparison and reproducibility. The exact prompt used for GSAI implementation is now fully provided in the Methods and detailed in the Supplementary Methods and Supplementary Fig. 8 to ensure transparency. In addition, all code, generated results, and embedding data are available in the accompanying zip file (see Code Availability), enabling independent replication of the full analysis pipeline. For completeness, we provide the text version of the prompt below for the reviewer's reference:

<task>
Analyze the following set of proteins that have been identified as potentially acting together in a particular biological context. Do their known roles and relationships suggest that there a specific process (or processes) that may be occurring? Do you see any strong relationships to specific disease processes or other phenotypes? It's OK if you don't see a significant pattern. Don't try to find one if it isn't there. In that case, just briefly describe the roles and interactions.

Be concise and factual. Only speculate about novel protein functions or interactions when you think there is very strong evidence. Be very clear when you are making a conjecture, a speculation. Use language such as "it might be the case that (speculation) based on (supporting reasoning)".

Be specific: Avoid overly general statements such as "the proteins are involved in various cellular processes".
</task>

<process>
Identify groups of proteins with similar functions or which you think interact in this context. For each group, briefly discuss their interplay, synergistic, or antagonistic effects, and functional integration within the system, instead of listing facts about individual proteins. Output no more than five groups. Do not mention any specific protein or gene names in the output.

After completing your analysis, please also assign a self-assessment confidence score to the process you selected. This score should range from 0.00 to 1.00. A score of 0.00 indicates the lowest confidence, while 1.00 reflects the highest confidence. This score helps gauge how accurately the chosen process name and the generated process description represent the functions and activities within the system of interacting proteins. When determining your score, consider the proportion of genes in the protein system that participate in the identified biological process. For instance, if you select "Ribosome biogenesis" as the process name but only a few genes in the system contribute to this process, the score should be lower compared to a scenario where a majority of the genes are involved in "Ribosome biogenesis".
</process>

<output>
For each group, make sure to follow this output format: "(LLM self-assessed confidence:) Process name, Process description". Avoid using any special symbols like the asterisk or the pound sign, or any highlighting. Don't generate a summary paragraph at the end.
</output>

<proteins>
{gene1, gene2, …)}
</proteins>

Comments on the code:

The code executes successfully.

It needs more detailed documentation for the required input and the optional input. For example, what are the background genes? If the researcher wanted to learn about a list of DEGs, would they be required to provide another list of background genes?

Response: We thank the reviewer for testing the code and for the constructive suggestions. We have expanded the documentation of the R package, particularly clarifying required and optional inputs, including the definition and role of background genes.

Reviewer #3 (Remarks to the Author):

We appreciate the extensive revisions and the substantial effort invested in expanding the analyses in this version. The manuscript is now more complete from a technical perspective. However, the major conceptual concerns raised in the previous round remain unresolved.

Response: We appreciate the reviewer's effort in re-evaluating our manuscript and for the insightful comment on the conceptual framing. In this revision, we have clarified and narrowed the manuscript's central contribution to directly address this concern: our goal is not to claim that embeddings replace curated annotation or enrichment, but rather to provide a missing statistical validation layer for LLM-inferred, gene-set-free functional hypotheses generated from gene lists. We now explicitly articulate this conceptual gap in the Abstract, Introduction, and Discussion, and demonstrate it empirically through (i) controlled benchmarking of gene-gene and gene-function relationships across focused and diffuse annotation categories, (ii) systematic contamination analyses that quantify discrimination between biological signal and noise, and (iii) evaluation on experimentally defined NeST protein systems, including direct comparisons with ORA and LLM confidence scores. Importantly, all primary analyses, including benchmarking and real-world applications, are now based on RAG-derived gene descriptions, ensuring that the framework substantively leverages literature-grounded representations rather than curated summaries. Together, these revisions establish that the framework addresses the conceptual problem of testing LLM-generated hypotheses with calibrated statistical evidence, rather than producing qualitative narratives alone. Detailed responses to each specific concern are provided below.

1. A central issue is that the framework continues to rely heavily on curated, highly structured gene summaries. Although RAG-generated summaries and LLM-generated descriptions were included in the revision, they do not materially improve performance or address the core dependence on curated text. As a consequence, the method still primarily reflects the information structure already encoded in these curated descriptions.

2. The input data still share substantial lexical and semantic overlap with the GO and pathway annotations used for benchmarking. This lack of independence between input and evaluation raises concerns about information leakage and makes it difficult to determine whether the reported performance reflects genuine semantic capability of LLM embeddings rather than the inherent structure of curated annotations.

Response: We thank the reviewer for highlighting these important issues and have combined them into a comprehensive response. We would first like to clarify that in the previous revision, we did not explicitly benchmark RAG-derived descriptions against curated NCBI summaries, but only reported their similarity to NCBI descriptions. Following the reviewer's suggestion, we have now performed a systematic comparison in the revised manuscript. Importantly, all primary analyses (Figs. 2-7) are now based exclusively on RAG descriptions derived from PubMed, while results using NCBI summaries are provided separately in Supplementary Figs. 1 and 6 for direct comparison. In this study, we generated four complementary RAG contexts per gene, including function, pathway, perturbation, and disease, enabling context-aware and flexible representations tailored to different biological evaluation scenarios. This context-specific design represents an

additional strength of incorporating RAG into our framework.

RAG descriptions are substantially longer and more context-rich than curated summaries (mean 170-181 words vs. 62 words for NCBI; Supplementary Table 2). Using these literature-grounded representations, top-performing models identified 96-99% of focused GO-BP and CP functions in gene-gene benchmarking (Fig. 3b) and achieved 96-98% sensitivity in gene-function analysis (Fig. 4b), with performance comparable to or exceeding that obtained using NCBI-based embeddings (Supplementary Fig. 6). Notably, RAG-based embeddings showed improved gene-function specificity compared with NCBI-based embeddings, with OpenAI-TE3 achieving 79.9% versus 73.7% under the 5% specificity criterion (Fig. 5f; corresponding NCBI results were reported in the previous revision). Performance also remained strong for GO-BP and CP terms newly added after the RAG literature cutoff (Figs. 3e, 4e, and 5d), supporting generalizability beyond the specific source text and reducing concerns about direct lexical overlap between input descriptions and evaluation annotations.

In addition, a substantial subset of embedding-derived clusters (18.7-30.8%, depending on the overlap criteria) showed no significant overlap with any GO-BP terms (lines 153-162, page 9). Ablation analyses further demonstrated highly distributed semantic contributions rather than dependence on a small number of keywords, as removal of the most frequent terms from all gene descriptions within a cluster resulted in only an average 0.02% decrease in intra-cluster cosine similarity (lines 139-152, pages 8-9; Fig. 2d). Together, these results indicate that the framework does not primarily recapitulate curated information structure or rely on simple lexical overlap, but instead leverages literature-grounded, context-aware representations for statistical evaluation of gene-function relationships.

3. The conceptual novelty over traditional ontology- or enrichment-based approaches remains limited. Although the embedding framework provides some incremental insights, these gains are modest—for example, only 7 of 103 clusters (6.8%) do not overlap with any GO term, and the MOA case study primarily highlights broad pathways that conventional DEG or network analyses would also recover. These observations suggest that the embedding representations, while useful, do not fundamentally alter the types of biological insights that can be obtained using existing methods.

Response: We thank the reviewer for this thoughtful comment. As clarified above, our goal is not to replace ontology- or enrichment-based approaches or to claim wholesale discovery of previously unknown biological themes. Rather, the conceptual contribution of this work lies in providing a calibrated statistical validation layer for LLM-inferred, gene-set-free functional hypotheses. This need is increasingly important as LLM-based functional inference methods are now being applied in practice, as demonstrated by recent studies (PMIDs: 39609565 and 40721871). The key advance of our framework is therefore methodological and inferential, not simply descriptive, enabling quantitative testing of language-derived functional hypotheses rather than relying on qualitative confidence scores alone.

Regarding the cluster analysis, the interpretation of novelty depends on the overlap criteria. As described above, with the RAG-based gene embeddings, 18.7-30.8% of embedding-derived

clusters showed no significant overlap with GO-BP under commonly used enrichment and Jaccard thresholds. These results demonstrate that RAG-derived descriptions, together with the embedding space, capture distributed and semantically coherent biological organization beyond direct ontology mapping. The ablation results and the lack of dependence on a small number of keywords further support this interpretation.

We also agree that the previous drug case studies and associated MOA analysis did not provide a sufficiently systematic demonstration. For this reason, in the revised manuscript we have replaced them with two more rigorous and conceptually aligned evaluations: (i) controlled contamination analyses that quantify discrimination between biological signal and noise, and (ii) benchmarking on 50 experimentally defined NeST protein systems used in recent LLM studies. In this setting, we directly compared embedding-based statistical prioritization with ORA and LLM confidence scores. Across NeST systems, GO-BP rankings derived from our embedding-based framework showed substantially higher agreement with the semantic reference ranking than ORA (median Spearman correlation 0.43 vs. 0.18; one-tailed Wilcoxon signed-rank test $P = 1.2 \times 10^{-14}$; Fig. 7a). For *de novo* LLM-inferred hypotheses (36 NeST systems with sufficient predictions), embedding-based prioritization achieved significantly higher concordance with the reference ranking than both ORA ($P = 9.3 \times 10^{-3}$; Fig. 7b) and confidence scores ($P = 0.021$). Moreover, hypotheses classified as significant by our framework were significantly more aligned with the reference ranking than non-significant predictions ($P = 2.4 \times 10^{-4}$), whereas ORA did not effectively distinguish coherent hypotheses ($P = 0.085$). These results are detailed in the revised Results section, "*Evaluation on LLM-inferred functions from real-world experimental gene lists*" (page 17; Fig. 7a-b).

Importantly, in the revised manuscript we carefully selected two NeST case studies to represent distinct biological scenarios: a highly coherent DNA repair module and a more heterogeneous regulatory module. In the ATM-dependent DNA repair system, our framework recovered multiple mechanistically coordinated DNA damage response hypotheses and demonstrated consistent gene-function connectivity beyond LLM confidence ranking alone, with improved prioritization relative to conventional ORA. In the ubiquitin-p53 regulatory system, the method quantitatively differentiated central ubiquitin-mediated growth control mechanisms from broader stress- and immune-related descriptions that were plausible but weakly supported by the gene composition, whereas ORA classified several of these broader terms as significant without clearly prioritizing the most mechanistically central pathway. This capacity to discriminate among competing, semantically related hypotheses and to prioritize the most statistically supported interpretation illustrates how embedding-based statistical testing can complement and, in certain contexts, refine conventional enrichment analysis, particularly for experimentally defined systems not directly represented in curated ontologies. The results are detailed in the section "*Representative case studies from NeST systems*" (page 18).

Thus, while embedding-based representations may recover pathways that enrichment analysis can also identify, the key advance of this framework lies in converting free-text LLM outputs into statistically testable hypotheses and enabling quantitative discrimination among competing functional interpretations. Rather than redefining biological themes, the framework provides a calibrated inferential layer that evaluates whether an LLM-generated function is truly supported by the gene set. This capability complements traditional enrichment approaches and strengthens the rigor of emerging gene-set-free LLM inference.

Overall, while the technical revisions improve the manuscript, the central issues remain, and the conceptual novelty of the proposed framework is still insufficient for Nature Communications.

Response: We thank the reviewer for the candid assessment. As detailed above, and following the reviewers' constructive suggestions, we have substantially revised the manuscript to clarify its conceptual contribution and strengthen the empirical evaluation. We respectfully believe the revised manuscript now presents a clearer and more rigorously supported methodological advance.

Reviewer #4 (Remarks to the Author):

I co-reviewed this manuscript with one of the reviewers who provided the listed reports. This is part of the Nature Communications initiative to facilitate training in peer review and to provide appropriate recognition for Early Career Researchers who co-review manuscripts.

Reviewer #4 (Remarks on code availability):

Given the concerns that we raised, it did not seem meaningful to review the code.

Response: We thank the Reviewer for co-reviewing the manuscript and for their thoughtful feedback. We understand the decision not to review the code given the broader concerns raised. For completeness, we note that another reviewer has tested the code and confirmed that it executes successfully. We remain open to any additional feedback should the Reviewer wish to provide further comments.

Reviewer #5 (Remarks to the Author):

I co-reviewed this manuscript with one of the reviewers who provided the listed reports. This is part of the Nature Communications initiative to facilitate training in peer review and to provide appropriate recognition for Early Career Researchers who co-review manuscripts.

Response: We thank the Reviewer for co-reviewing the manuscript and providing their constructive feedback.

Manuscript #: NCOMMS-25-46979B-Z
Revised Title: An embedding-based framework enables statistical testing of gene-set function hypotheses inferred by large language models

Reviewer #2 (Remarks to the Author):

Comments:

This revision provides a substantial improvement over the previous version. We appreciate the author's serious effort in addressing the critiques from the last review. The narrative is now clearer, the foundation is stronger, and the conceptual contribution is better positioned. We detail below what has been addressed well, followed by the remaining points.
One concern in the previous round was the lack of a quantitative comparison with gene set enrichment. The authors have now rigorously implemented this across 50 NeST protein systems, showing that GO-BP rankings from the embedding framework achieve substantially higher concordance with the semantic reference ranking than those from ORA.
Another critical concern the authors addressed was the use of anecdotal examples in hypothesis testing. This version has removed the two examples and shifted the theme from hypothesis testing to a more systematic evaluation of the statistical output from their method. This change has made their method more practical and the narrative clearer.
In addition, the contamination analysis has addressed the concern raised about the lack of negative controls.
The RAG pipeline has been expanded to include four context-specific query types: function, pathway, perturbation, and disease. This addresses the concern that RAG was previously treated as a secondary or future direction.

Response: We thank the reviewer for reviewing our manuscript again and for the positive feedback. We hope our revisions and clarifications have satisfactorily addressed the reviewer's suggestions.

Major concerns:

The authors attribute lower performance on GWAS and CGP gene sets to the heterogeneous nature of complex disease biology, and leave it at that. This framing treats heterogeneity as a caveat rather than recognizing it as the most scientifically interesting challenge for this framework. The idea of using LLM-based approaches is that LLMs could go beyond what curated databases already offer. The context-specific RAG descriptions with disease-specific retrieval suggest the authors are already moving in this direction. The Discussion should explicitly state this, framing context-dependent gene function as a forward-looking opportunity rather than a limitation.

Response: We appreciate and fully agree with the reviewer's insightful comment. We revised the Discussion section (pages 26-27) to more explicitly frame context-dependent and multifactorial perturbation and disease biology as an important opportunity for LLM- and embedding-based approaches, rather than primarily as a challenge for the framework. We additionally clarify that approaches leveraging RAG-derived, context-specific biological knowledge have the potential to

enable increasingly context-aware and mechanistically informed interpretation of complex molecular phenotypes across diverse biological and disease contexts.

A clear schematic should be included in Figure 7 that details the approach the authors used for function inference and comparisons.

Response: We thank the reviewer for this helpful suggestion. To improve methodological transparency and facilitate result interpretation of the comparison workflows used in the NeST analyses, we added schematic flowcharts in Fig. 7a,b illustrating the design of the evaluation pipelines.

Revised Fig. 7a,b:

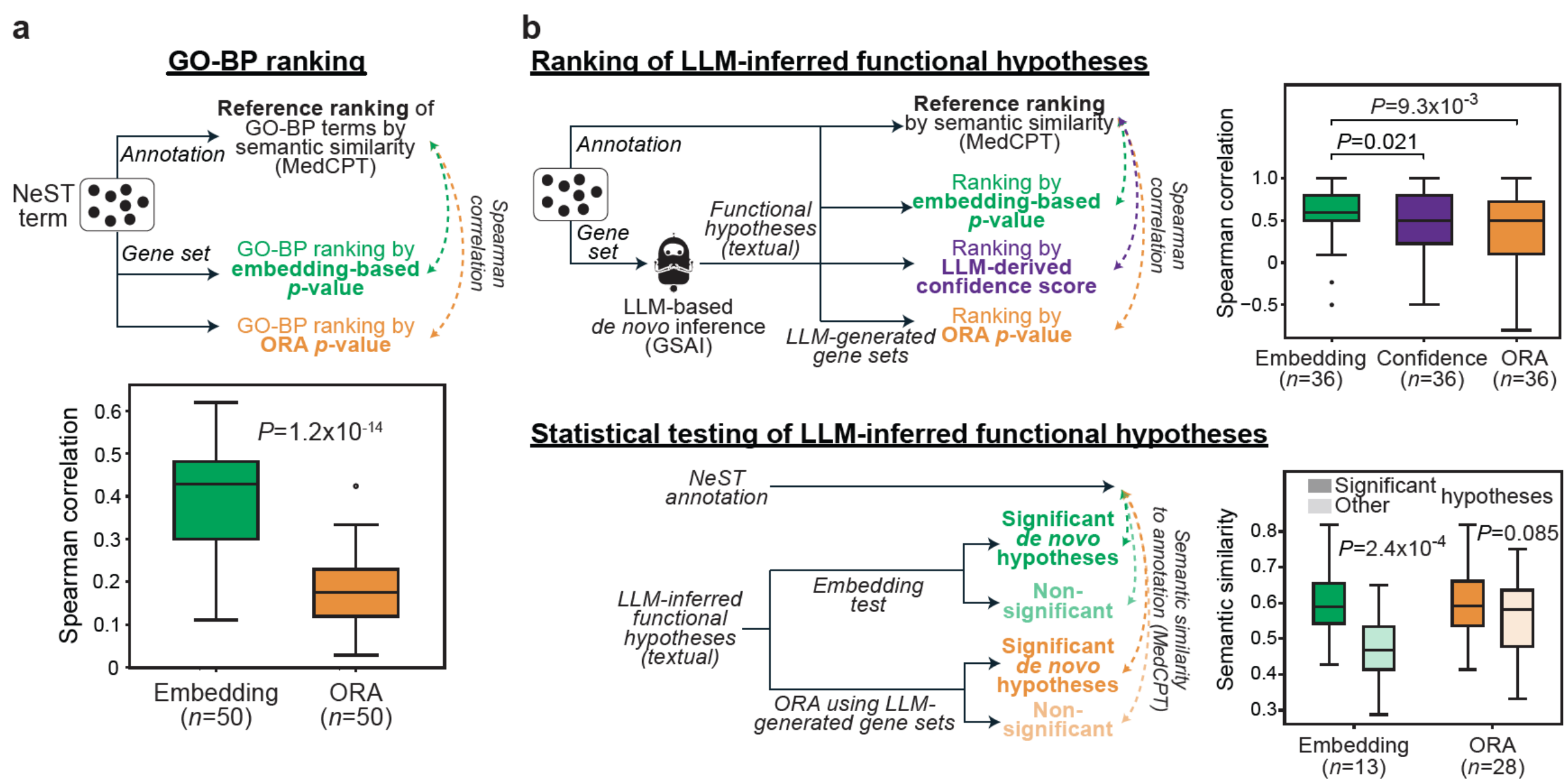

The semantic reference ranking in Figure 7 is constructed using MedCPT embeddings, while MedCPT is simultaneously one of the seven evaluated models. The authors should briefly justify this design choice and clarify that it does not affect the central OpenAI-TE3 vs. ORA comparison.

Response: We thank the reviewer for this important suggestion. In the revised Results (see below; pages 17-18) and Methods section (page 37), we now explicitly clarify that the semantic reference ranking for NeST terms was constructed using the MedCPT Query Encoder, following a recent study (Wang *et al*. *Nature Methods* 2025; PMID: 40721871). Importantly, the MedCPT model included in our comparative evaluation (Figs. 2-5) was a different model designed for text embedding, namely the MedCPT Article Encoder. These are distinct encoder models trained for different tasks, and therefore the use of the Query Encoder to construct the semantic reference ranking for NeST terms does not affect the central comparison between OpenAI-TE3 and ORA or the comparative evaluation of embedding models presented in the manuscript.

Results (pages 17-18)

"To define a reference ranking, we calculated MedCPT-based semantic similarity between GO-BP term names and the corresponding NeST annotation using the MedCPT Query Encoder model, following a recent study6 (Methods). This Query Encoder is distinct from the MedCPT model evaluated in previous sections (the Article Encoder)."

Methods (page 37)

"To construct a semantic reference ranking, we computed cosine similarity between embeddings generated by the MedCPT Query Encoder embeddings (https://huggingface.co/ncbi/MedCPT-Query-Encoder) for GO-BP term names and the corresponding NeST annotation. The choice of the MedCPT Query Encoder followed a recent study[6] and is distinct from the MedCPT Article Encoder evaluated as one of the embedding models elsewhere in this study."

The ORA used in the NeST evaluation is performed on GPT-4o-generated gene sets rather than the standard enrichment workflow using curated database gene sets. This should be labeled clearly. And please clarify why standard enrichment approaches were not used to find enriched terms for the NeST gene sets.

Response: We appreciate this constructive suggestion. In the revised manuscript, we now clarify that the ORA for LLM-inferred functions was performed using GPT-4o-generated gene sets rather than conventional curated annotation gene sets, because the LLM-inferred functions are *de novo* textual hypotheses that may not directly correspond to existing annotations. Corresponding clarifications have been added to the Results section (see below; page 18) and the flowchart in Fig. 7b.

"*Unlike the previous evaluation, which directly assessed enrichment of predefined GO-BP terms from the NeST gene lists, this analysis evaluated free-text inferred functional concepts generated by an LLM (Fig. 7b). … For comparison, we implemented ORA using GPT-4o-generated gene sets corresponding to each inferred function, because these de novo textual hypotheses may not directly correspond to existing curated gene sets or ontology terms, thereby enabling direct comparison under a matched inference setting (Fig. 7b; Methods).*"

Minor concerns:

The de novo functional inference (Fig. 7b) showed a sample size of 36 instead of the 50 gene sets from the original pool. Is the missing gene set due to a lack of functional enrichment/GSAI function proposal? Or other reason, please clarify.

Response: We thank the reviewer for noticing this discrepancy. The reduced sample size in Fig. 7b reflects the filtering criteria used for the Spearman correlation analysis. Specifically, NeST systems with fewer than three inferred functional hypotheses from GSAI were excluded because rank correlation analysis was not informative in these cases. We have clarified this criterion in the Fig. 7b legend.

Reviewer #3 (Remarks to the Author):

The manuscript has been substantially improved at the technical level, with expanded benchmarking and clearer positioning as a validation framework. However, the manuscript does not demonstrate that the method provides a necessary or decision-altering capability within typical functional genomics workflows (such as Gene Ontology). In particular, the current analyses primarily benchmark different LLM models under their own framework with existing annotations (GO) rather than demonstrating a distinct capability beyond established approaches. As such, the contribution appears to be an incremental refinement of existing approaches rather than a conceptual or practical advance. Therefore, I still feel that the manuscript does not meet the standard for novelty in Nature Communications.

Response: We thank the reviewer for the thoughtful and constructive feedback. We appreciate the reviewer's concern regarding whether the framework provides a necessary and decision-altering capability within realistic functional genomics workflows beyond benchmarking semantic similarity or comparing LLMs against existing annotations. Following the reviewer's feedback and the editor's guidance, we substantially expanded both the conceptual framing and practical demonstrations of the framework in the revised manuscript to directly address this point in two ways.

First, we more clearly articulated the NeST protein assembly analyses through two detailed case studies with side-by-side comparisons of embedding-based statistical testing, conventional overrepresentation analysis (ORA), and LLM-derived confidence scoring within experimentally derived protein systems ("*Representative case studies from NeST systems*," starting on page 19; Fig. 7; Supplementary Table 7). These analyses highlight situations in which embedding-based statistical testing alters functional prioritization and mechanistic interpretation relative to existing approaches. Specifically, the framework distinguished strongly supported mechanistic hypotheses from broader but weakly differentiated alternatives generated by *de novo* LLM-based inference, thereby providing quantitative prioritization beyond qualitative confidence scoring alone.

Second, and more importantly, we added a new Results section entitled "*DEGEmbedR implementation for de novo functional annotation of differentially expressed genes (DEGs)*" (pages 21-23; new Fig. 8). This addition was specifically designed to demonstrate practical integration of our open-source R package, DEGEmbedR, into routine functional genomics workflows. DEGEmbedR implements embedding-based statistical testing for both predefined annotations and *de novo* LLM-inferred functional hypotheses, enabling quantitative prioritization within standard DEG interpretation pipelines. As a representative use case, we applied DEGEmbedR to a LINCS/L1000 drug perturbation-derived DEG signature from 5'-N-ethylcarboxamidoadenosine (NECA)-treated MCF7 cells, which was previously reported to lack significant functional annotation by conventional ORA. In this setting, conventional enrichment analysis yielded no statistically significant annotations, whereas LLM *de novo* inference generated multiple biologically plausible but weakly differentiated hypotheses without quantitative prioritization. In contrast, embedding-based statistical testing prioritized a smaller subset of mechanistically coherent hypotheses centered on extracellular matrix interaction, cell adhesion, and inflammatory signaling, while down-weighting broader and less specific processes related to general cell cycle regulation and metabolism, thereby altering downstream mechanistic

interpretation and hypothesis prioritization. To further assess biological consistency, we incorporated orthogonal validation using large-scale pharmacogenomic drug sensitivity analyses across pan-cancer cell lines, which identified extracellular matrix- and cell-cell interaction-related genes as strong predictors of NECA sensitivity, together with supporting evidence from the literature.

We revised the Abstract and Introduction (page 6) accordingly and added a new Discussion paragraph (pages 25-26) further clarifying the conceptual and practical advances of the framework. Specifically, the revised manuscript now emphasizes that the primary contribution of the framework is not simply benchmarking LLM models against existing annotations, but rather providing a quantitative prioritization and statistical testing framework for *de novo* functional hypotheses generated by LLMs, particularly in settings where predefined annotation-based enrichment approaches provide limited guidance. Through the expanded NeST analyses and the new DEGEmbedR implementation with a drug perturbation DEG case study, the revised manuscript further demonstrates that embedding-based statistical testing can alter functional prioritization and mechanistic interpretation in realistic functional genomics workflows. To our knowledge, this study is the first to introduce a statistically grounded framework that systematically determines whether LLM-inferred gene-set functions are quantitatively supported by the input genes, thereby enabling statistically guided integration of generative LLM inference into practical functional genomics workflows.

We hope these substantial revisions have satisfactorily addressed the reviewer’s concerns and clarified the novelty, practical utility, and broader significance of the study.

Reviewer #4 (Remarks to the Author):

I co-reviewed this manuscript with one of the reviewers who provided the listed reports. This is part of the Nature Communications initiative to facilitate training in peer review and to provide appropriate recognition for Early Career Researchers who co-review manuscripts.

Response: We thank the reviewer for co-reviewing the manuscript and for their thoughtful feedback.

Reviewer #5 (Remarks to the Author):

I co-reviewed this manuscript with one of the reviewers who provided the listed reports. This is part of the Nature Communications initiative to facilitate training in peer review and to provide appropriate recognition for Early Career Researchers who co-review manuscripts.

Response: We thank the reviewer for co-reviewing the manuscript and providing their constructive feedback.
